# Genome resequencing reveals the genetic basis of population evolution, local adaptation, and rewiring of the rhizome metabolome in *Atractylodes lancea*

**DOI:** 10.1093/hr/uhae167

**Published:** 2024-06-21

**Authors:** Chengcai Zhang, Sheng Wang, Jiahui Sun, Xiangkong Li, Hongyang Wang, Xiuzhi Guo, Yuefeng Wang, Daiquan Jiang, Chaogeng Lyu, Chuanzhi Kang, Yan Zhang, Zengxu Xiang, Qingjun Yuan, Binbin Yan, Ming Qin, Luqi Huang, Lanping Guo

**Affiliations:** State Key Laboratory for Quality Ensurance and Sustainable Use of Dao-di Herbs, National Resource Center for Chinese Materia Medica, China Academy of Chinese Medical Sciences, Beijing 100700, China; Key Laboratory of Biology and Cultivation of Herb Medicine, Ministry of Agriculture and Rural Affairs, Beijing 100700, China; Dexing Research and Training Center of Chinese Medical Sciences, Dexing 334220, China; State Key Laboratory for Quality Ensurance and Sustainable Use of Dao-di Herbs, National Resource Center for Chinese Materia Medica, China Academy of Chinese Medical Sciences, Beijing 100700, China; Key Laboratory of Biology and Cultivation of Herb Medicine, Ministry of Agriculture and Rural Affairs, Beijing 100700, China; Dexing Research and Training Center of Chinese Medical Sciences, Dexing 334220, China; State Key Laboratory for Quality Ensurance and Sustainable Use of Dao-di Herbs, National Resource Center for Chinese Materia Medica, China Academy of Chinese Medical Sciences, Beijing 100700, China; Key Laboratory of Biology and Cultivation of Herb Medicine, Ministry of Agriculture and Rural Affairs, Beijing 100700, China; Novogene Bioinformatics Institute, Beijing, China; State Key Laboratory for Quality Ensurance and Sustainable Use of Dao-di Herbs, National Resource Center for Chinese Materia Medica, China Academy of Chinese Medical Sciences, Beijing 100700, China; Key Laboratory of Biology and Cultivation of Herb Medicine, Ministry of Agriculture and Rural Affairs, Beijing 100700, China; State Key Laboratory for Quality Ensurance and Sustainable Use of Dao-di Herbs, National Resource Center for Chinese Materia Medica, China Academy of Chinese Medical Sciences, Beijing 100700, China; Key Laboratory of Biology and Cultivation of Herb Medicine, Ministry of Agriculture and Rural Affairs, Beijing 100700, China; State Key Laboratory for Quality Ensurance and Sustainable Use of Dao-di Herbs, National Resource Center for Chinese Materia Medica, China Academy of Chinese Medical Sciences, Beijing 100700, China; Key Laboratory of Biology and Cultivation of Herb Medicine, Ministry of Agriculture and Rural Affairs, Beijing 100700, China; State Key Laboratory for Quality Ensurance and Sustainable Use of Dao-di Herbs, National Resource Center for Chinese Materia Medica, China Academy of Chinese Medical Sciences, Beijing 100700, China; Key Laboratory of Biology and Cultivation of Herb Medicine, Ministry of Agriculture and Rural Affairs, Beijing 100700, China; State Key Laboratory for Quality Ensurance and Sustainable Use of Dao-di Herbs, National Resource Center for Chinese Materia Medica, China Academy of Chinese Medical Sciences, Beijing 100700, China; Key Laboratory of Biology and Cultivation of Herb Medicine, Ministry of Agriculture and Rural Affairs, Beijing 100700, China; State Key Laboratory for Quality Ensurance and Sustainable Use of Dao-di Herbs, National Resource Center for Chinese Materia Medica, China Academy of Chinese Medical Sciences, Beijing 100700, China; Key Laboratory of Biology and Cultivation of Herb Medicine, Ministry of Agriculture and Rural Affairs, Beijing 100700, China; State Key Laboratory for Quality Ensurance and Sustainable Use of Dao-di Herbs, National Resource Center for Chinese Materia Medica, China Academy of Chinese Medical Sciences, Beijing 100700, China; Key Laboratory of Biology and Cultivation of Herb Medicine, Ministry of Agriculture and Rural Affairs, Beijing 100700, China; College of Horticulture of Nanjing Agricultural University, Nanjing 210095, China; State Key Laboratory for Quality Ensurance and Sustainable Use of Dao-di Herbs, National Resource Center for Chinese Materia Medica, China Academy of Chinese Medical Sciences, Beijing 100700, China; Key Laboratory of Biology and Cultivation of Herb Medicine, Ministry of Agriculture and Rural Affairs, Beijing 100700, China; State Key Laboratory for Quality Ensurance and Sustainable Use of Dao-di Herbs, National Resource Center for Chinese Materia Medica, China Academy of Chinese Medical Sciences, Beijing 100700, China; Key Laboratory of Biology and Cultivation of Herb Medicine, Ministry of Agriculture and Rural Affairs, Beijing 100700, China; Dexing Research and Training Center of Chinese Medical Sciences, Dexing 334220, China; Dexing Research and Training Center of Chinese Medical Sciences, Dexing 334220, China; State Key Laboratory for Quality Ensurance and Sustainable Use of Dao-di Herbs, National Resource Center for Chinese Materia Medica, China Academy of Chinese Medical Sciences, Beijing 100700, China; Key Laboratory of Biology and Cultivation of Herb Medicine, Ministry of Agriculture and Rural Affairs, Beijing 100700, China; State Key Laboratory for Quality Ensurance and Sustainable Use of Dao-di Herbs, National Resource Center for Chinese Materia Medica, China Academy of Chinese Medical Sciences, Beijing 100700, China; Key Laboratory of Biology and Cultivation of Herb Medicine, Ministry of Agriculture and Rural Affairs, Beijing 100700, China

## Abstract

The formation of high-quality Chinese medicinal materials is a micro-evolutionary process of multiple genes involving quantitative inheritance under environmental stress. *Atractylodes lancea* is a traditionally used medicinal plant in China that is broadly distributed and possesses a considerable amount of essential oils. However, to date, limited research has been conducted to characterize the genetics and metabolites of *A. lancea* shaped by natural variation. Hence, we assembled a high-quality genome of *A. lancea*, featuring a contig N50 of 1.18 Mb. We further integrated population resequencing of *A. lancea* and conducted analyses to characterize its genetic diversity, population evolution, and rewiring of volatile metabolites. The natural variation effect exerted significant pressure on *A. lancea* from different geographic locations, resulting in genetic differentiation among three groups. Correlation analysis of metabolites in *A. lancea* revealed significant natural variations of terpenoids, heterocyclic compounds, ketones, and esters. We also found that 427 metabolites displayed noteworthy divergence due to directional selection. Additionally, our genome-wide association studies on the metabolome for medicinal quality traits identified several candidate genes, such as *AlZFP706* and *AlAAHY1*, exhibiting significant correlations with atractylodin and hinesol levels, respectively. Overall, this study provides an intricate genomic resource for *A. lancea*, thereby expanding our understanding of the effect of natural variation on metabolites and facilitating the genetic improvement of its medicinal properties.

## Introduction

Differential selection pressures imposed by spatial heterogeneity in the environment may contribute to the local adaptation of a species throughout its geographic range [1]. Relevant research results indicate that for naturally distributed plants most of the selection pressure comes from environmental factors such as precipitation, light, soil chemistry, and pH [2]. These factors can promote adaptive genetic differentiation of species based on spatial changes in selection processes [3]. Adaptive divergence has an impact on gene expression, metabolism, and physiology and determines the phenotypic changes of species, thereby increasing their adaptability to local environments. Medicinal plants with higher genetic diversity arise as a result of long-term adaptive microevolution, thereby driving a stronger local adaptation to new ecological niches, which is more pronounced in widely distributed source area species. [4]. Some studies found that the chemical composition changes of widely distributed species exhibit significant continuity properties. The composition of volatile oils in Chinese peppermint (Bohe), *Chrysanthemum* (Juhua) and *Atractylodes* (Cangzhu) may shift from a continuous to a discontinuous pattern in response to significant changes in the climate of their distribution regions, particularly influenced by dominant factors affecting their chemical composition [5, 6]. Remarkable differences are noted in the composition of volatile oil of the rhizome of *Atractylodes lancea* from the Dao-di area (called Dao-di herbs) and those from other regions. The Dao-di area refers to the Maoshan region of Jiangsu province, which is more drought-prone and potassium-deficient than other regions [6]. The variations in the composition of Dao-di herbs are closely related to their environmental adaptability, which suggests that these plants can adapt well to complex external environments. Therefore, one of the abiding rules of evolutionary genetics is to develop a better understanding of genetic variants to control adaptive differences among natural populations by identifying the genomic and geographic extent of adaptation [1].

*Atractylodes* DC. is an endemic genus within the Asteraceae family, found extensively across East Asia with five species, namely, *A. lancea*, *A. carlinoides*, *A. coreana*, *A. macrocephala*, and *A. japonica*. The two traditional Chinese herbal remedies Cangzhu and Baizhu are derived from *Atractylodes* species. With the exception of *A*. *carlinoides*, all species within the genus have found applications as herbal medicines. Their dried rhizomes have mainly been used for digestive disorders and rheumatic diseases. Genome sequencing analysis has been conducted on various medicinal plants, including *Morinda officinalis*, *Forsythia suspensa* (weeping forsythia), *Coptis chinensis*, and *Eriobotrya japonica* [7, 8]. The species *A. lancea* (2*n* = 2*x* = 24) presents a cross-pollinated plant with extremely high heterozygosity and variability. This genetic profile positions it as a well-defined model for investigating population differentiation and local adaptation. Throughout their lifespan, plants engage in constant exchange of material and energy with their surrounding environment [9]. Numerous factors, including climate, soil, terrain, biology, and human activities, influence the distribution of plant resources [10]. As a result, the environment plays an essential role in determining plant distribution. In addition, there is an obvious relationship between plant secondary metabolism and the environment. On the one hand, plant secondary metabolites (PSMs) provide protective mechanisms against environmental stresses, such as ultraviolet radiation, moisture, and temperature. On the other hand, external stimuli, including light, water content, temperature, CO_2_ concentration, and humidity, can also influence plant secondary metabolism. The presence of PSMs enables plants to effectively adapt to environmental changes and thrive better in a new ecological environment. Environmental factors can significantly influence PSMs. These metabolites represent outcomes of long-term evolutionary adaptation to plants’ ecological environment, serving as the material foundation for protecting plants from unfavorable conditions [11]. Currently, metabolite-based genome-wide association studies (mGWAS) have become a prevalent approach for investigating environmental adaptability and mechanisms of adaptation in plants [12]. The successful application of mGWAS in various model plants and agriculturally significant species has established novel connections between genes and metabolites [13–15]. Nonetheless, research on the adaptive microevolution of PSMs and genetic basis of medicinal plants is generally lacking.

In the current investigation, we constructed a high-quality, chromosome-scale genome for *A. lancea*, employing it as a benchmark for a population genomic exploration involving 251 individuals gathered from 22 populations spanning the species’ distribution. Subsequently, we examined the genetic diversity of the species, as well as its population structure. Furthermore, we undertook an extensive metabolic profiling endeavor, followed by a subsequent mGWAS involving 130 accessions within an *A. lancea* population. The findings obtained shed light on the genetic variation linked to the population evolution and local adaptation of *A. lancea*, offering valuable insights for the verification and traceability of the geographic origin of this species.

## Results

### Genome assembly and annotation

The estimated size of the *A. lancea* genome was 4162.72 Mb, with a heterozygosity rate of 1.65% and a repeat rate of 82.78%, as determined through *k*-mer analysis (Supplementary Data Table S1, Supplementary Data Fig. S1). Several genomic libraries were constructed. Multiple genomic libraries were meticulously constructed, yielding an impressive dataset, including roughly 589.90 Gb of Illumina data with short-fragment sequencing (~141.71×), 580.84 Gb of PacBio data (~139.53×), and 639.98 Gb of clean Hi-C reads (Supplementary Data Table S2). These invaluable resources were leveraged in the assembly process, culminating in the generation of a draft genome of 4009.19 Mb, with scaffold N50 of 289.33 Mb and contig N50 of 1.18 Mb (Table 1, Supplementary Data Table S3). Notably, these figures closely align with the estimates derived from the *k*-mer analysis (Supplementary Data Table S1). Based on the Hi-C data, roughly 90.90% scaffolds were anchored to 12 pseudochromosomes (Supplementary Data Fig. S2, Table 1, Supplementary Data Table S4).

**Table 1:** Final results of assembly and annotation of the *A. lancea* genome

**Genome assembly**	**Statistic**
Evaluating genome size (Mb)	4162.72
Genome size (Mb)	4009.19
Contig N50 (Mb)	1.18
Pseudochromosomes	12
Gap number	69
GC content (%)	38.43
Repetitive sequences (%)	71.61
BUSCO completeness (%)	89.90
Transposable elements (%)	69.59
Protein-coding genes	48 492

**Figure 1:**
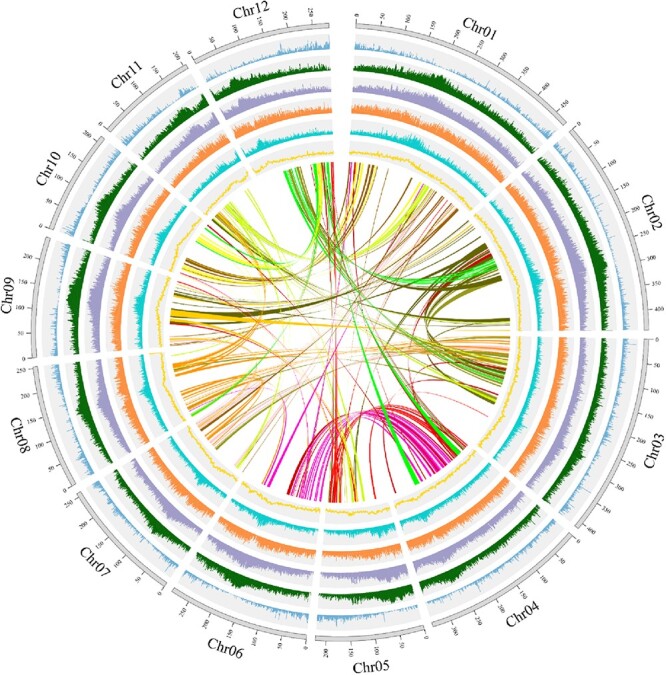
Circos diagram of various elements on *A. lancea* chromosomes. The outermost circle (grey) represents 12 pseudomolecule chromosomes. The bar charts of circles 2–8 indicate gene density, distributions of TEs, LTR density, *Copia* density, *Gypsy* density, GC distribution, and intraspecific collinearity, respectively. All sections are drawn based on window size = 1 Mb and chromosome units = 1 Mb.

The remapping rate reached 97.71% when aligning the reads obtained from Illumina to our assembled genome. We assessed our genome assembly completeness utilizing the Conserved Core Eukaryotic Gene Mapping Approach (CEGMA) and Benchmarking Universal Single-Copy Orthologs (BUSCO) assessments of genome completeness (Supplementary Data Table S5). The results revealed a total of 235 (94.76%) core eukaryotic genes (CEGs) and 1294 (89.90%) complete gene models (Supplementary Data Tables S6 and S7). These findings support the completeness and outstanding quality of our genome assembly. A total of 2870.47 Mb (71.61%) of the assembly was identified as repetitive elements and annotated accordingly (Supplementary Data Table S8). Among these repetitive elements, 69.59% were transposable elements (TEs), with 63.61% classified as long terminal repeat (LTR) retrotransposons, making up the largest proportion of repetitive elements. Additionally, most LTRs were found to be *Gypsy* elements and *Copia* repeats (Supplementary Data Table S9, Fig. 1, Supplementary Data Fig. S4).

Using different databases, we identified 50 658 protein-coding genes (PCGs). Each gene displayed 4.54 exons on average, and the mean coding domain sequence (CDS) length was 1004.59 bp in *A. lancea* (Supplementary Data Table S10). Through homology in at least one of the queried databases, 48 492 genes (95.70%) were functionally annotated (Table 1). A total of 39 370 (77.70%) genes contained Pfam domains, and 33 026 (65.20%) genes were successfully assigned Gene Ontology (GO) terms (Supplementary Data Table S11). Furthermore, the annotation also included non-coding RNAs, encompassing 3414 microRNAs (miRNAs), 452 rRNAs, 3132 tRNAs, and 3635 small nuclear RNAs (snRNAs), with average lengths of 121.74, 188.49, 71.91, and 110.62 bp, respectively (Supplementary Data Table S12).

### Comparative genomic and evolutionary analysis of *A. lancea*

**Figure 2:**
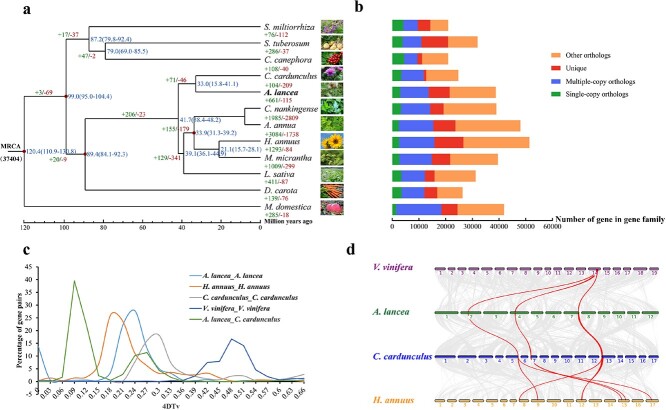
Comparison of gene families and evolution of the *A. lancea* genome. **a** Phylogenetic relationships between 12 species. Divergence time is shown in blue. Expanded gene families are shown in green and contracted families in red. **b** Distribution of genes. Single-copy orthologs, multiple-copy orthologs, unique, and other orthologs are shown in green, blue, red, and yellow, respectively. **c** Proportional distribution of *K*_s_ for paralogous gene pairs. Vertical and horizontal axes correspond to the proportion and *K*_s_ value of gene pairs, respectively. **d** Distribution of 4DTv.

To investigate the genome evolution and divergence of *A. lancea*, this study employed OrthoMCL to compare its sequence similarity with 11 other species (*Artemisia annua*, *Coffea canephora*, *Chrysanthemum nankingense*, *Cynara cardunculus*, *Helianthus annuus*, *Lactuca sativa*, *Malus domestica*, *Mikania micrantha*, *Daucus carota*, *Salvia miltiorrhiza*, and *Solanum tuberosum*) (Supplementary Data Table S13). We identified in total 37 426 gene families, which encompassed 50 658 genes, within the *A. lancea* genome (Supplementary Data Table S13). Additionally, we pinpointed 191 common single-copy orthologous gene families. Protein sequences from these 191 gene families were utilized to establish the phylogenetic relationship between *A. lancea* and the other species (Fig. 2b). *Atractylodes lancea*, *C. cardunculus*, *H. annuus*, *Artemisia annua*, *C. nankingense*, *M. micrantha*, and *L. sativa* were clustered in the same branch. The phylogenetic relationship of other species was as previously reported. *Atractylodes lancea* and *C. cardunculus* formed a subclade, and diverged from each other at ~33.0 million years ago (Mya; 15.8–41.1) (Fig. 2a). We utilized the CAFÉ software to identify expanded or contracted gene families across the 12 species. In the most recent common ancestor (MRCA), 37 404 gene families were estimated. A comparison of the MRCA of *A. lancea* and *C. cardunculus* revealed 661 expanded and 115 contracted gene families in *A. lancea* (Fig. 2a). In addition, a phylogenetic analysis of 18 species, including *Vitis vinifera* and *Arabidopsis thaliana*, was also constructed (Supplementary Data Fig. S3). The 661 expanded gene families underwent functional pathway analysis using the KEGG database, revealing 20 KEGG pathways showing significant enrichment with *P*_adj_ <0.05 (Supplementary Data Table S14). The enrichment of sesquiterpenoid and triterpenoid biosynthesis (map00909) revealed the genomic foundation underlying the high sesquiterpenoid content in *A. lancea*, with a total of 37 expanded genes contributing to sesquiterpenoid biosynthesis. Among the gene families identified in *A. lancea* and the 11 other species, 2275 genes appeared unique to *A. lancea*. We also found that a few *A. lancea*-specific genes were also enriched in sesquiterpenoid, triterpenoid, and terpenoid backbone biosynthesis pathways (Supplementary Data Table S15). Meanwhile, enrichment analyses based on GO terms of the *A. lancea*-specific genes indicated that they are particularly enriched in these terms: cysteine-type peptidase activity (GO:0008234), proteolysis (GO:0006508), and peptidase activity, acting on l-amino acid peptides (GO:0070011) (Supplementary Data Table S16).

**Figure 3:**
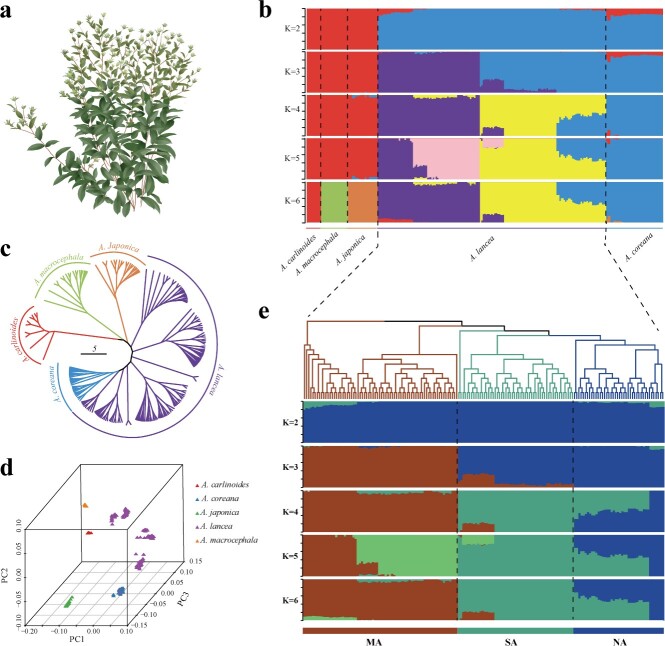
Phylogenetic relationships and population structure of wild *Atractylodes* species. **a** Plant morphology of *A. lancea*. **b** Population structure of wild *Atractylodes* species individuals based on ADMIXTURE analysis with *k* = 2–6. **c** Neighbor-joining (NJ) phylogenetic tree of wild *Atractylodes* species samples using whole-genome SNP data based on genetic distances. **d** PCA of wild *Atractylodes* species. **e** Population structure analysis of 161 accessions of *A. lancea*. The 161 accessions included 72 MA accessions, 54 SA accessions and 35 NA accessions.

The synteny among *A. lancea* chromosomes indicated the possibility of WGD events. To assess these potential WGD events of *A. lancea*, the 4-fold degenerate site (4DTv) and *K*_s_ results of duplicated gene pairs were estimated based on the syntenic blocks detected among *A. lancea* and another three plant genomes (Fig. 2c, Supplementary Data Fig. S5). A genetic distance peak at 0.24 was observed among paired genes located in duplicated collinear blocks in the *A. lancea* genome, suggesting a recent WGD event in *A. lancea*. The distribution of *C. cardunculus* 4DTv sites showed a peak at 0.3, indicating a later WGD event in *C. cardunculus*, consistent with its phylogenetic relationship. Based on the *K*_s_ of the duplicated gene pairs, a peak was present at the *K*_s_ value of 1.5, suggesting a probable WGD event in *A. lancea*. The peak at around 0 in *A. lancea* might have been caused by tandem repeats. According to the *K*_s_ distribution, the divergence peak (dotted line in Supplementary Data Fig. S5) of *A. lancea* and *C. cardunculus* was closer to the *y*-axis than the double peak (solid line in Supplementary Data Fig. S5), indicating that *A. lancea* and *C. cardunculus* shared a common WGD event, and then underwent species divergence (Supplementary Data Fig. S5). Additionally, we confirmed that the *A. lancea* genome was related to those of *V. vinifera*, *C. cardunculus*, and *H. annuus*, and the collinearity analysis indicated that each *V. vinifera* region had up to three *A. lancea* regions, while each *A. lancea* region had up to two *H. annuus* regions (Fig. 2d). The synteny analyses provided further evidence that *A. lancea* and *C. cardunculus* did share the *γ*-event that occurred within eudicots, as shown by the 1:3 syntenic relationship between *V. vinifera* and *A. lancea.*

### Basic population genetic characteristics of *Atractylodes* species

To enhance our understanding of genomic variation within the *Atractylodes* species, we resequenced 251 *Atractylodes* accessions from 20 cities in China, including 161 accessions from eight provinces across major *A. lancea*-growing areas, 21 accessions of *A. japonica*, 10 accessions of *A. carlinoides*, 40 accessions of *A. coreana*, and 19 accessions of *A. macrocephala.* (Supplementary Data Fig. S6, Supplementary Data Table S1). Clean data totaling 12.36 Tb were generated, with all the accessions subsequently aligned to the *A. lancea* genome. This process yielded an average alignment ratio of 92.97% and an average depth of 12.97-fold. This study identified 2 705 925 high-quality SNPs. Among them, 91 225 annotated SNPs (3.37%) were in the coding regions, and 52 281 (1.93%) were identified as non-synonymous SNPs.

To unveil the genetic underpinnings of *Atractylodes* populations, we employed three methods for inferring population structure: principal component analysis (PCA), a phylogenetic tree using neighbor-joining [16], and an SNP-based analysis from 251 accessions. The findings consistently revealed differences in genetic structures among the five investigated *Atractylodes* species and different *A. lancea* accessions (Fig. 3b–d, Supplementary Data Table S2). Furthermore, we found that *A. lancea* accessions from different distribution areas also exhibited three different subgroups, and the three groups exhibited distinct geographic distributions: wild accessions in the Maoshan-Dabie Mountains group (MA) mostly from five regions (AHHS, HBLT, AHLA, JSJR, and JSNJ); the North Yanshan Mountain group (NA) mainly from northwest China, including Hebei and Inner Mongolia provinces (HBLH, HBCD, and NMGZLT); and the Qinling-Taihang Mountains group (SA) from SXXY, HBYX, HNSX, and SXWZ (Fig. 3e). In addition, we calculated *k* values ranging from 2 to 8 and their corresponding error values of cross-validation (Supplementary Data Fig. S7, Supplementary Data Table S17). At a *k* value of 6, the population genetic divergence aligned with the results obtained from the neighbor-joining tree and PCA. The population results revealed significant genetic differences among the five species, with *A. coreana* and *A. lancea* being more closely genetically related, thus providing the first genomic evidence for the diversity of the *Atractylodes* species population. To further elucidate the processes of local adaptation and natural variation of Atractylodes species, we conducted a detailed genetic diversity analysis based on SNP markers. Pixy is a tool designed for calculation within and between population genomics statistics such *d*_xy_, π, and *F*_ST_. In this study, we analyzed genome-wide diversity of five *Atractylodes* species, using all sites (variable and invariant sites), and the results indicated that nucleotide diversity (θπ) was lower in the *A. lancea* (θπ = 5.70E−03), *A. japonica* (θπ = 4.77E−03), *A. macrocephala* (θπ = 4.35E−03), and *A. carlinoides* (θπ = 2.39E−03) populations than in *A. coreana* (θπ = 6.19E−03) (Supplementary Data Table S18, Supplementary Data Fig. S8). Compared with other groups, *d*_xy_ (7.63E−03) was the highest in comparison between *A. lancea* and *A. macrocephala*, while *F*_ST_ (0.47) was the highest in the group of *A. carlinoides* and *A. macrocephala* (Supplementary Data Table S19). Subsequently, the TreeMix analysis revealed substantial gene flow between *A. lancea* and *A. coreana*, whereas there was no observable gene flow between *A. lancea* and *A. macrocephala* (Supplementary Data Fig. S9). Differences in genetic diversity between *A. lancea* and *A. coreana* were not significant, which is consistent with the tree structure.

We further analyzed the genetic structure of *A. lancea* (Fig. 3a) from various regions, supported by model-based clustering analysis (Fig. 3e). As mentioned above, 161 accessions from different distribution areas were divided into three *A. lancea-*ecotypes: NA, SA, and MA. Notably, we found that the MA group contains samples from the Dao-di areas (JSJR and JSNJ), which indicates that the genetic structure of *A. lancea* in the population from the Dabie Mountains is close to that from the Maoshan area (Jiangsu province, China).

### Metabolome alteration to natural variation of A. lancea from different distribution areas

To evaluate natural variations of the metabolome in *A. lancea* rhizomes (ALRs), we collected rhizome samples in China from 130 *A. lancea* accessions based on their genetic structure (Supplementary Data Table S3). These accessions included 45 SA accessions, 34 NA accessions and 51 MA accessions (Supplementary Data Table S4). Next, we quantified the relative content of 1427 metabolites (Supplementary Data Table S4) in rhizomes of *A. lancea* using gas chromatography–tandem mass spectrometry (GC–MS/MS). Among 130 accessions, 62.93% of all metabolites exhibited observed coefficients of variation exceeding 1 (Supplementary Data Fig. S10, Supplementary Data Table S5). Almost all detected metabolites followed a normal distribution, indicating that they are controlled by several loci (Supplementary Data Fig. S11). Furthermore, the variability in metabolite content was greater in the MA group compared with the NA and SA groups, aligning with the observed levels of genetic diversity across the three groups.

**Figure 4:**
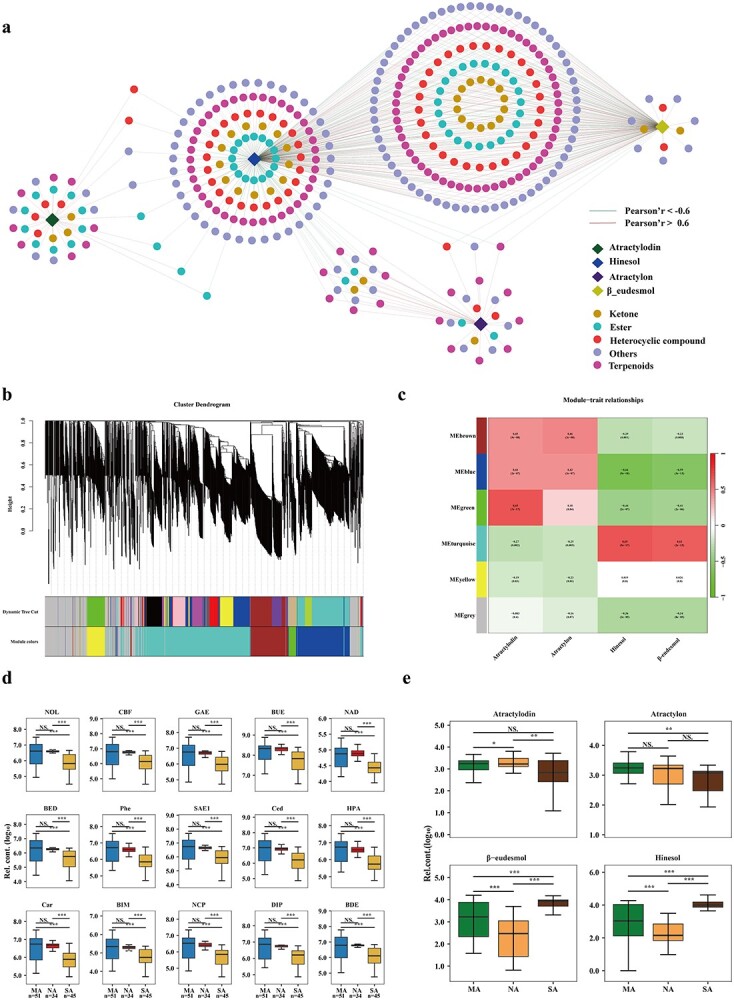
**a** Interaction network of the four essential oil components and 1427 metabolites based on Pearson correlation. Edges connecting two parts represent the weight (transformed MR score) for the association. Metabolites and the four essential oil components (β-eudesmol, hinesol, atractylodin, and atractylon) are colored according to their compound names. Network maps were drawn using the edge-weighted spring-embedded layout in Cytoscape (https://cytoscape.org). **b** Clustering dendrogram of metabolites, with dissimilarity based on topological overlap, together with assigned module colors. **c** Heat map of four essential oil components. Metabolite expression patterns in a matrix shows module–trait relationships. The expression patterns of six modules are shown by the heat map, with a color scale of expression levels from high (red) to low (green). The correlation coefficient is presented for each cell, followed by values of −log(*P*), where *P* is Fisher’s exact test *P*-value. **d** Box plot of 15 important metabolites in *A. lancea* with different variations among the MA, NA, and SA groups. **e** Box plot of atractylodin, β-eudesmol, hinesol, and atractylon in *A. lancea* with different variations among the MA, NA, and SA groups. Metabolic data were log_10_-transformed. Data in (**d**) and (**e**) are presented as mean ± standard deviation. ^*^P < 0.05; ^**^P < 0.01; ^***^P < 0.001 (Student’s *t*-test).

Subsequently, we identified the principal active constituents in *A. lancea*, including three sesquiterpenoids—β-eudesmol, hinesol, and atractylon—along with a polyacetylene compound, atractylodin. Our investigation then centered on quantifying the aforementioned essential oils from samples collected in various distribution regions (Fig. 4e, Supplementary Data Table S6). Utilizing Spearman’s rank correlation, the associations between the contents of metabolites were assessed (Supplementary Data Fig. S12, Supplementary Data Table S7). Terpenoids and heterocyclic compounds, identified as the primary bioactive compounds in *A. lancea* [17], were significantly correlated with four essential oil components (Fig. 4a). Previous investigations conducted by our research team indicated that the four bioactive compounds of *A. lancea* under examination in this study may serve as chemical indicators for verifying and tracing the geographic provenance of plant specimens. In this study, after removing duplicates, a total of 408 metabolites were found to have significant correlation with the four essential oil components (|*r*| > 0.6). Seventy-eight compounds exhibited positive correlations with atractylodin and atractylon in the network diagram, whereas 122 compounds displayed significant negative correlations with β-eudesmol and hinesol (Fig. 4a, Supplementary Data Table S8). Recent research has indicated that heterocyclic metabolites, terpenoids, ketones, and esters contribute to the medicinal constituents found in ALRs (Zhang C *et al*., 2023). Thus, it can be inferred that both terpenoids and heterocyclic metabolites represent the primary characteristic compounds selected for rewiring during local adaptation.

Weighted Gene Co-expression Network Analysis (WGCNA) was further employed to establish a metabolite coexpression network, identify significant modules, and identify key metabolites. In constructing the WGCNA network, we initially determined the soft thresholding power β, which is used to exponentiate the coexpression similarity for adjacency calculation. The pickSoftThreshold function was used to construct the network and analyze its topology. Next, we set value 12 as the soft thresholding power β. This choice was informed by the achievement of a scale independence value of 0.85 and a relatively high-average connectivity (Supplementary Data Fig. S13). Ultimately, six modules of the four highly correlated *A. lancea* essential oils were eventually constructed (Fig. 4b and c). The most abundant metabolite modules included the turquoise module (No. 4, 545 metabolites), blue module (No. 2, 250 metabolites), and brown module (No. 1, 7177 metabolites). Metabolites in the turquoise module were largely correlated with β-eudesmol and hinesol, whereas those in the blue and brown modules were closely associated with atractylodin and atractylon, respectively (Fig. 4c). The blue and brown modules contained 427 metabolites that are significantly positively correlated with atractylodin and atractylon as being subjected to independent adaptation of the *A. lancea* metabolome (Supplementary Data Table S9). There were 174 differentially abundant metabolites in NA (*P* < 0.05 and average NA/MA ratio >1) and 319 differentially abundant metabolites in SA (*P* < 0.05 and average SA/MA ratio <1) compared with MA, of which 56 metabolites (MA > NA > SA) exhibited similar dynamic trends (Supplementary Data Fig. S14, Supplementary Data Table S10). Fifteen metabolites were identified as hub metabolites using a threshold of GS over 0.2 and MM over 0.7 (Fig. 4d, Supplementary Data Tables S11 and S20). Eventually, 15 compounds accumulated in MA and NA, including γ-elemene (GAE), cedrol (Ced), and carotol (Car) (Fig. 4d). Moreover, atractylodin and atractylon accumulated to higher levels in the MA and NA groups than in SA, while both hinesol and β-eudesmol levels decreased in NA and MA (Fig. 4e), suggesting that metabolome alterations in different distribution areas are probably associated with local adaptation. These metabolites specific to each subspecies likely indicate the differentiation among the *A. lancea* germplasm subspecies, particularly among the three major ones.

### Genetic basis of metabolite diversity in *A. lancea* rhizomes

**Figure 5:**
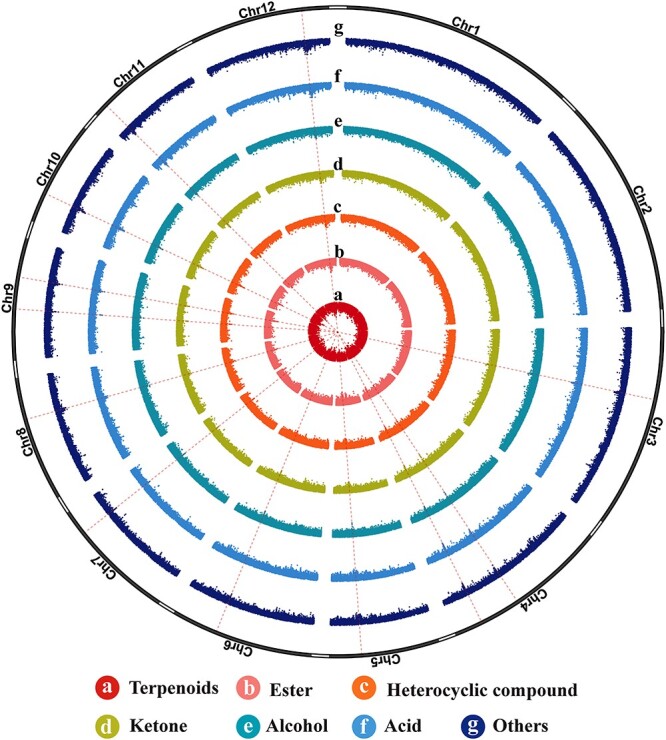
Genomic distribution of significant SNPs associated with 427 metabolites. The circles from the center outwards represent terpenoids (**a**), esters (**b**), heterocyclic compounds (**c**), ketones (**d**), alcohols (**e**), and other compounds (**f**). The *P*-values of all significant SNPs associated with metabolites are listed in Supplementary Data Table S12.

To unveil the genetic underpinnings of the detected variants in *A. lancea* metabolic traits, we performed an mGWAS on 130 accessions of *A. lancea* using a linear mixed model (LMM) via GEMMA, which accounts for genome-wide genetic correlation patterns to mitigate false positives (Fig. 5). Manhattan plots displaying significant SNPs associated with various metabolite categories, including terpenoids, esters, heterocyclic compounds, ketones, alcohols, and others, are illustrated in Fig. 5. In addition, we found that the significant SNPs associated with these compounds co-localized on chromosomes 3–12, suggesting that these SNPs were important for the medicinal quality of *A. lancea*. Following the outlined filtering procedures, a total of 511 candidate genes were pinpointed in connection with 690 lead SNPs, potentially attributing them to the observed variations in metabolic traits (Supplementary Data Table S12). A total of 9493 significant SNPs associated with 427 key *A. lancea* metabolites were detected, 5425 candidate genes were also identified (Supplementary Data Tables S12 and S13), and 122 genes were selected in all four types of metabolites (Supplementary Data Table S14, Supplementary Data Fig. S15). Based on population analysis of MA vs NA and MA vs SA (*F*_ST_ and π), as well as GWAS analysis with four types of metabolite, a total of 14 genes intersected with them, and 4 genes appeared in all four metabolic phenotypes (Supplementary Data Fig. S15, Supplementary Data Table S15). Considering the incomplete elucidation of synthetic pathways for these metabolites, the regulatory mechanisms of these genes are expected to be of considerable interest.

It was well known that atractylodin, atractylon, β-eudesmol, and hinesol are the principal active substances in *A. lancea*. To ensure data accuracy, we determined the levels of these four active substances in 130 varieties using an absolute quantitative method. Similarly, we performed mGWAS on 130 *A. lancea* accessions employing the LMM facilitated by GEMMA (Supplementary Data Fig. S16). In total, 2315 significant SNPs were identified in association (Supplementary Data Table S16). The results yield valuable insights into the genetic underpinnings of metabolomic variations and offer functional elucidations of the underlying pathways.

### Selective signatures associated with local adaptation in *A. lancea* from different distribution areas

**Figure 6:**
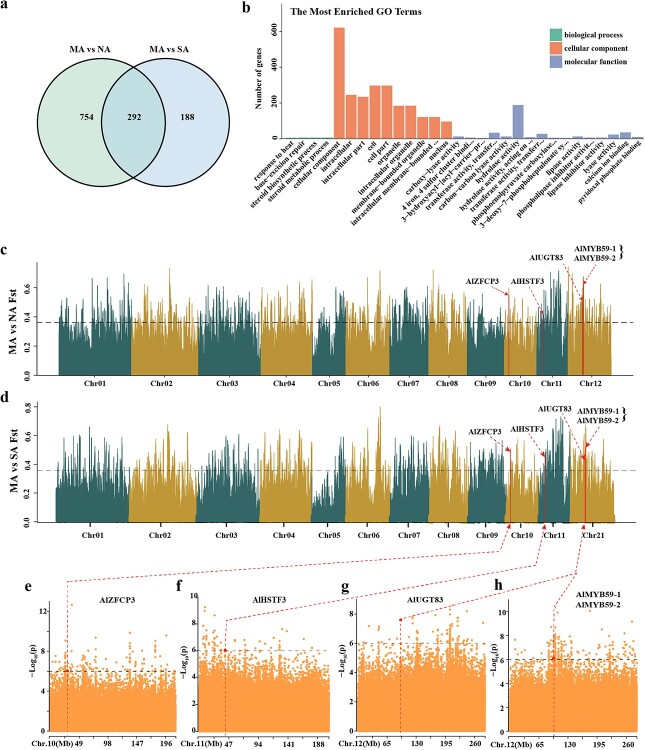
Population features and divergence of *A. lancea*. **a** Comparison of selected genes among three different groups. **b** Most enriched GO terms of 292 commonly selected genes. **c**, **d** Highly divergent genomic regions among three different groups (MA, NA, and SA). The horizontal dashed line indicates the top 5% of *F*_ST_. Red vertical lines indicate the genes that concern high-divergence regions. **e**–**h** Local Manhattan plots of GWAS signals overlapping with high-divergence genomic regions for four key environmental adaptability genes.

To capture potential genes under divergent selection associated with local adaptation, we examined the genome-wide *F*_ST_ values and nucleotide diversity π in *A. lancea* with different variations among the MA, NA, and SA groups based on the SNP array data of 130 samples from three *A. lancea* groups. The accessions employed in this investigation span diverse environmental conditions, potentially contributing to the observed population structure. We calculated the population differentiation (*F*_ST_) between MA and NA and discovered a selected region spanning 92.72 Mb, which included 1046 genes (*F*_ST_ ≥ 0.36, log_2_(θπ_NA_/θπ_MA_) ≥ 0.44), ranking within the top 5% of the *F*_ST_ distribution (Fig. 6c, Supplementary Data Table S19). In the combination of MA and SA, the selected region of MA is 45.44 Mb, containing 480 genes (*F*_ST_ ≥ 0.35, log_2_(θπ_SA_/θπ_MA_) ≥ 0.58) (Supplementary Data Table S20, Supplementary Data Fig. S19), whereas the selected region of SA is 88.86 Mb, containing 750 genes (*F*_ST_ ≥ 0.35, log2(θπ_MA_/θπ_SA_) ≥ 0.57) (Fig. 6d, Supplementary Data Table S21). In two combinations, MA vs NA and MA vs SA, the intersection of selected areas in MA is 25.32 Mb, containing 292 genes (Fig. 6a, Supplementary Data Table S22). In our study, the 292 shared selected genes were further classified into three functional subcategories (corrected *P* values <0.05) in the GO classification. Furthermore, among the three groups, the 292 selected genes were further classified into three functional subcategories (corrected *P* values <0.05) in the GO categorization (Fig. 6b, Supplementary Data Table S23), and 1046 selected genes in MA vs NA and 480 genes in MA vs SA were also subjected to GO enrichment analysis (Supplementary Data Figs S17 and S18).

We hypothesize that a portion of the high-*F*_ST_ regions contribute to the differentiation observed between the two groups. Within the high-*F*_ST_ regions, three genes encoding transcription factors (TFs) were identified as undergoing positive selection between the two groups. These include evm. Model. ctg4454.9 and evm. Model. ctg4454.12 (*AlMYB59-1* and *AlMYB59-2*) encoding MYB TFs, and evm. Model. ctg4147.56 (*AlHSTF3*) encoding a TF involved in heat stress (Fig. 6f and h). In addition, another two genes, evm. Model. ctg4451.49 (*AlUGT83*) and evm. Model. ctg3908.11 (*AlZFCP3*), encoded a UDP-glycosyltransferase and a CCCH zinc finger protein, respectively (Fig. 6e and g, Supplementary Data Table S19). MYB plays pivotal roles in various aspects of plant biology, including growth regulation, secondary metabolism, and transcriptional regulation under biotic/abiotic stresses [18, 19]. The CCCH zinc finger protein regulates drought response in the root of *Populus ussuriensis* and is known to play a role in salinity, drought, and cold stresses [40, Zhang H *et al*., 2019]. Strong selective signals [*F*_ST_ ≥ 0.35 were highlighted in five genes (*AlMYB59-1*, *AlMYB59-2*, *AlUGT83*, *AlUGT83*, and *AlZFCP3*)] (Fig. 6c and d) known to be important regulatory genes for stress responses [20], indicating potential roles for these genes in local adaptation in *A. lancea* across various distribution areas.

### Genetic variants linked to medicinal quality traits in *A. lancea*

**Figure 7:**
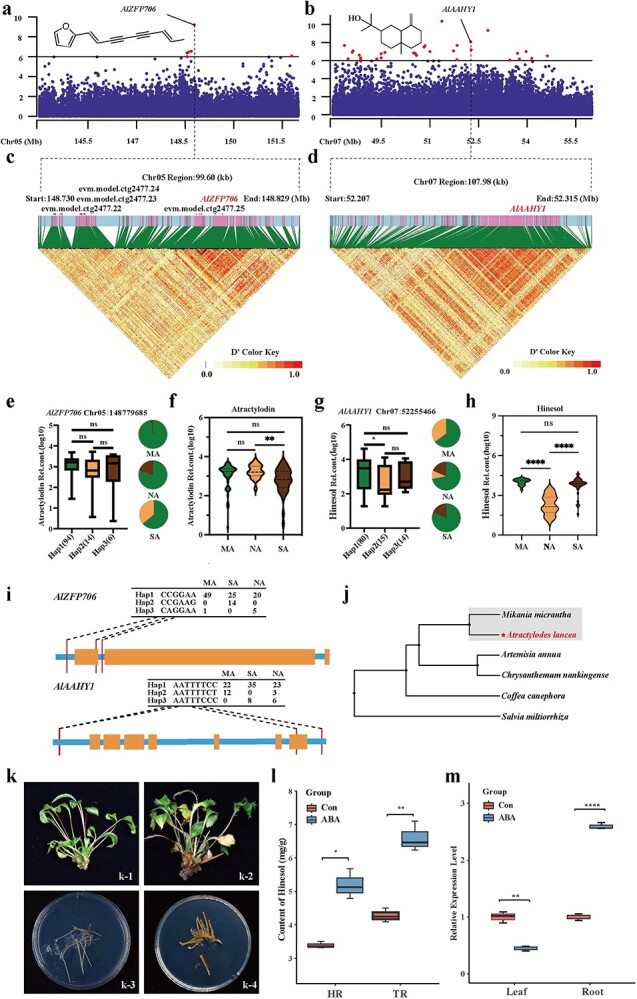
Identification of genes associated with medicinal quality traits of *A. lancea*. **a** Manhattan plot displaying GWAS results of atractylodin content. Metabolite content was genetically associated with the sweep harboring *AlZFP706*. The dashed line indicates the threshold −log_10_*P* = 6. **b** Manhattan plot displaying GWAS results of hinesol content. Metabolite content was genetically associated with the sweep harboring *AlAAHY1*. The dashed line indicates the threshold −log_10_*P* = 6. **c** Representation of pairwise *r*^2^ value (a measure of LD) among all polymorphic sites in a region of ~99.6 kb around *AlZFP706*. **d** Representation of pairwise *D*′ value (a measure of LD) among all polymorphic sites in a region of ~107.98 kb around *AlAAHY1*. **e** Frequencies of the high/low-level trait-related allele in the three groups (MA, NA, and SA). Box plot shows relative contents of atractylodin, plotted as a function of genotypes at SNP Chr05:148779685. Atractylodin contents were log_10_-transformed, nHap. **f** Violin plot of *A. lancea* atractylodin contents in different variations among the MA, NA, and SA groups. **g** Frequencies of the high/low-level trait-related allele in the three groups (MA, NA, and SA). Box plot shows relative contents of hinesol plotted as a function of genotypes at SNP Chr07:52255466. Hinesol contents were log_10_-transformed, nHap. **h** Violin plot of *A. lancea* hinesol contents in different variations among the MA, NA, and SA groups. **i** Gene structure diagram of *AlZFP706* and *AlAAHY1*. **j** Phylogenetic tree of *AlZFP706*. **k** (1-4) Growth status of *A. lancea* tissue cultured seedlings without added ABA for 12 days; Growth status of *A. lancea* tissue cultured seedlings treated with 100 μM ABA for 12 days; Growth status of hairy roots of A. lancea without added ABA after 12 days; Growth status of hairy roots of *A. lancea* with addition of 100 μM ABA after 12 days. **l** Hinesol content in hairy roots (HR) and tissue-cultured seedling roots (TR) of *A. lancea* after 30 days of growth*.* Red bar chart indicates no addition of ABA and blue bar chart represents addition of 100 μM ABA. **m** Relative expression levels of *AlAAHY1* gene in *A. lancea* tissue-cultured seedling (roots and leaves) treated with 100 μM ABA and non-treated samples.

As mentioned above, the population structure of different *A. lancea* germplasms has been extensively studied. Nonetheless, the impact of these variations on the four essential oil components remains poorly understood. To discover genetic loci associated with *A. lancea* medicinal quality traits, GWAS was performed for β-eudesmol, hinesol, atractylodin, and atractylon contents (Fig. 7). Through GWAS analysis, we associated multiple signals with β-eudesmol, hinesol, atractylodin, and atractylon (Supplementary Data Table S25). Among these genes, we screened 10 genes that are significantly correlated with the local adaptation of *A. lancea*, including one gene for β-eudesmol, namely *AlUGF3OG6* (Supplementary Data Fig. S22); four genes for hinesol, namely *AlBZFP32* (Supplementary Data Fig. S23), *AlAAHY1* (Fig. 7b), *AlNDKCM* (Supplementary Data Fig. S24), and *AlPCKSB4* (Supplementary Data Fig. S25); four genes for atractylodin, namely *AlZFP706* (Fig. 7a), *AlCTA12* (Supplementary Data Fig. S26), *AlPSPTA25520* (Supplementary Data Fig. S27), and *AlGTF25* (Supplementary Data Fig. S28); and one gene for atractylon, namely *AlPVPSP13* (Supplementary Data Fig. S29). We then observed the location and structural information of *AlZFP706* and *AlAAHY1* and found that the SNP sites were both located upstream of the genes (Supplementary Data Fig. S32). The functions of their orthologs in *Arabidopsis* [21–23] indicate their essential roles in growth and development of plants under both favorable conditions and stresses.

Atractylodin has been found to have antitumor, antibacterial, antiviral, and antiarrhythmic effects, especially on the gastrointestinal tract and nervous system, making it a key component of ALRs (Zhang *et al*., 2023). In the metabolomics data, we observed a decrease in atractylodin levels of SA compared with NA, indicating negative selection in the SA group during natural variations of the metabolites in ALRs (Fig. 7f, Supplementary Data Table S26). The GWAS for atractylodin identified a total of 78 significant genomic regions comprising 149 genes (Supplementary Data Fig. S20, Supplementary Data Table S27). A gene on chromosome 5 (evm. Model. ctg2477.26), encoding zinc finger protein 706, namely *AlZFP706*, was pinpointed in the selective sweep between the NA and SA groups (Fig. 7a, Supplementary Data Table S24). Three haplotypes of *AlZFP706* were found in 114 varieties, hap3 appeared in MA and SA varieties, and hap2 appeared only in SA varieties (Fig. 7e, f, and i). Among them, SNP 148779685 (on chromosome 5), located in the 17 075-bp upstream evm. Model. ctg2477.26, overlapped with a domestication sweep between the NA and SA groups (Supplementary Data Figs S20 and S32, Supplementary Data Tables S2 and S26). To delve into the evolutionary history of the *AlZFP706* gene identified in *A. lancea*, we conducted amplification of its homologous genes from diverse species, encompassing *M. micrantha*, *A. annua*, *C. nankingense*, *C. canephora*, and *S. miltiorrhiza* (Fig. 7j). The results indicate that *AlZFP706* in *A. lancea* is more closely related to *M. micrantha*.

Hinesol and β-eudesmol may be potential candidates for cancer treatment [24]. According to the metabolomics analysis, hinesol decreased from the NA group to the MA group (Fig. 7h, Supplementary Data Table S29), suggesting negative selection for hinesol during *A. lancea* natural variation in the NA group. The GWAS analysis targeting hinesol content revealed 158 candidate genes across 64 genome regions showing significant associations (Supplementary Data Fig. S21, Supplementary Data Table S28). An abscisic acid 8′-hydroxylase gene (*AlAAHY1*) encoded on chromosome 7 (evm. Model. ctg3071.8) was also highlighted in the selective sweep between the NA and SA groups (Supplementary Data Fig. S32, Supplementary Data Table S25). For *AlAAHY1*, three haplotypes were found in 109 varieties, with hap2 occurring in MA and NA varieties and hap3 in NA and SA varieties; correspondingly, accessions harboring hap2 produced lower hinesol content. It suggested that different haplotypes of *AlAAHY1* gene may be related to hinesol content (Fig. 7g–i). Collectively, *AlAAHY1* likely plays a key role in modulating hinesol content.

To further characterize the 10 selected candidate genes significantly correlated with the medicinal quality traits of *A. lancea*, we conducted expression pattern analysis on these 10 genes. qPCR results verified the expression of all 10 genes in tissue-cultured seedlings of *A. lancea*. Additionally, they exhibited differential expression in the roots and leaves of tissue-cultured seedlings. The most significantly expressed genes were *AlAAHY1*, *AlCTA12*, *AlGTF25*, *AlNDKCM*, *AlPSPTA25520*, *AlUGF3OG6*, and *AlZFP706*. *AlBZFP32*, *AlPCKSB4*, and *AlPVPSP13* did not show differential expression in roots and leaves (Supplementary Data Fig. S30). Among these 10 genes, we found one gene (*AlAAHY1*) annotated as abscisic acid 8′-hydroxylase. To validate the function of *AlAAHY1*, we administered 100 μM of abscisic acid (ABA) to the culture medium of tissue-cultured seedlings (Supplementary Data Fig. S31a) and hairy roots (Supplementary Data Fig. S31b) of *A. lancea*, and measured the changes in hinesol levels associated with *AlAAHY1*, indirectly validating the function of *AlAAHY1* gene. After 12 days of culture with the addition of ABA, the leaves of the experimental group (tissue-cultured seedlings of *A. lancea*) turned yellow and withered, and browning appeared at the base of the stem (Fig. 7k-2), while the control group (without added ABA) presented with dark green leaves and growth in good condition (Fig. 7k-1). Compared with the control group (Fig. 7k-3), browning was more evident in the hairy roots of *A. lancea* treated with 100 μM of ABA (Fig. 7k-4). Further, by measuring the hinesol levels of tissue-cultured seedlings and hairy roots of *A. lancea* treated with ABA, we found that the hinesol levels in both the hairy roots and the tissue-cultured seedlings’ roots significantly increased compared with the control group (Fig. 7l). Under ABA treatment, *AlAAHY1* was downregulated in leaves but induced in roots, suggesting its potential role in the ABA-mediated stress response (Fig. 7m). Our experimental results indicate that *AlAAHY1* might contribute to the accumulation of hinesol, further enhancing the stress resistance of *A. lancea*.

## Discussion

Few population evolution and local adaptation studies of *A. lancea* have been reported to date due to the lack of genomic resources. As a result, exploring the mechanism behind the accumulation of secondary metabolites and effectively regulating their biosynthesis in *A. lancea* presents a significant challenge. In the current research, we analyzed high-quality genome assemblies for studying *A. lancea* and resequenced 251 individuals of *Atractylodes* species. To the best of our knowledge, this is the first study to report genetic variation and environmental adaptation in *Atractylodes* species based on whole genome sequencing of *A. lancea* and their related species. These genomic data enhance our comprehension of the local adaptation and evolutionary history of *Atractylodes*.

A robust and comprehensive reference genome is fundamental for various population genetics investigations and experimental studies. Utilizing PacBio sequencing for genome assembly coupled with error correction using Illumina data significantly enhances the continuity and completeness of the assembly [25]. Our approach to genome assembly for *A. lancea* resulted in a highly resolved assembly with the longest contig length of 8.27 Mb and an N50 of 1.18 Mb. Recent breakthroughs in sequencing technology have enabled gap-free genome assembly from telomere to telomere (T2T). This advancement significantly improves the continuity and completeness of the T2T genome [26, 27]. Notably, genome sequencing efforts have been undertaken for over 100 medicinal plants [28, 29]. Among them, *Taxus* has the largest genome size, reaching 10.23 Gb [30], followed by *Ginkgo biloba* (9.87 Gb) [31], *Gnetum montanum* (4.07 Gb) [32], and *Panax ginseng* (3.43 Gb) [33]. The genome size of *A. lancea* that we assembled is 4.009 Gb, which is one of the larger genomes among medicinal plants. In addition, the *A. lancea* genome we obtained has a relatively high heterozygosity of 1.65%, which is close to that of the *Clematis montana* genome (1.95%) [34]. A high-heterozygosity genome can confer many advantages on this plant. First, it can increase the adaptability and survival ability of individuals. Different gene alleles can provide different functions and characteristics, which can have effects in different environments and thus make individuals more adaptable to diverse conditions. Second, a high-heterozygosity genome can enhance the stability and resistance of genes. Different gene alleles can compensate for each other, thereby reducing the risk of gene mutations and deletions. At the same time, the presence of different gene alleles can also increase the resistance of genes, making individuals healthier and stronger. For example, the 2.27% heterozygosity of ‘Feizixiao’ resulted in high heterosis at the genetic level, which can be used for the breeding of excellent varieties of lychee [35].

We constructed the phylogenetic tree of *A. lancea* and its relatives based on genomic data and found that *A. carlinoides* and *A. macrocephala* separated first, then *A. japonica*, *A. coreana*, and *A. lancea* complex successively split off in that order, which is consistent with previous phylogenetic analyses within the genus *Atractylodes* [36] Based on morphological and genetic data, *Atractylodes* represents a small genus consisting of five species, which is consistent with the classification result reported in the *Flora Republicae Popularis Sinicae* [37]. In the current study, we show that *A. lancea* complex and *A. coreana* have a relatively close genetic relationship. Based on genomic and metabolomic distinctions, *A. lancea* complex was clustered into three subpopulations: NA, SA, and MA (Zhang *et al*., 2023). The significant intraspecific variability observed may be attributed to genetic and metabolic differences, aligning with the description in the *Flora of China* of *A. lancea* as a ‘highly variable and polymorphic species’. These findings suggested that metabolic phenotypes were closely related to the geographic variability of *A. lancea*, suggesting that metabolic profiling serves as a valuable tool for exploring plant evolution and local adaptation [38]. Kleessen *et al*. also demonstrated for *Arabidopsis* accessions that both genotypic variability and phenotypic traits related to flowering and metabolism robustly correlate with the geographic origin of the plants [39]. This study revealed both contracted and expanded gene families in *A. lancea*, with the expanded families primarily resulting from tandem duplications. These expanded families show significant enrichment in pathways associated with sesquiterpenoid and triterpenoid biosynthesis.

From the numerous genome variations of *A. lancea* accessions, which include wild samples from various distribution areas, we identified genomic regions that have undergone 92.72-Mb selective sweeps. In these regions, we identified 1046 candidate genes associated with distinct natural variations in both MA and NA groups. Additionally, we also observed that there is a clear geographic and genetic division between the MA and SA groups. The selected region in the MA group spans 45.44 Mb and contains 480 genes. Additionally, the analysis demonstrated a joint selection of 292 genes in both comparative groups, suggesting their key roles in the natural variations of *A. lancea*. Through selective sweep analysis, we further explored how environmental factors impact the quality of *A. lancea* across various distribution areas, we further explored how environmental factors impact the quality. This investigation revealed enriched functional categories among the selected genes included multi-organism processes, response to temperature stimulus, response to heat, and carboxy-lyase activity. Notably, a gene encoding a cytochrome P450 enzyme, previously reported to be involved in plant evolution and metabolic diversification, was identified as related to local adaptation [40]. Another gene, *AlMYB59*, which encodes a transcription factor and is known to regulate K^+^/NO_3_^−^ transport under potassium deficiency in *Arabidopsis*, was found on chromosome 12 [41]. Additionally, a gene encoding a heat stress TF (*AlHSTF3*), which regulates transcriptional memory after heat stress, was identified on chromosome 11 [42]. Adaptive plasticity in stress responses determine the survival of plants. The TF HEAT SHOCK FACTOR (HSF) plays a significant role in transcriptional heat responses and plant heat stress memory. Our previous studies have reported that Dao-di *A. lancea* faces challenges in nutrient availabilities. Experiments in a greenhouse demonstrated that heat and low-K stress can give rise to plants that produce essential oil compounds resembling those from Dao-di herbs. *AlMYB59* and *AlHSTF3* in *A. lancea* may contribute to its enhanced environmental adaptability and geographic distribution. It is worth mentioning that MYB TFs form the most extensively studied TF families in plants. Their annotation in plants has provided valuable insights into their functions. Therefore, the presence of *AlMYB59* and *AlHSTF3* in *A. lancea* likely contributes to its adaptability to different environments and broad geographic distribution.

Plant metabolites regulate diverse aspects of plant physiology, such as growth, nutrient allocation, cellular replenishment, and environmental adaptation [43]. Thus, the metabolome serves as a link between its genotype and phenotype [44]. Relevant research has revealed that metabolic variations in a species are more frequent than initially anticipated [45], and this genetic diversity is responsible for the natural variations in metabolites [46]. Here, we found significant variability in the metabolites of *A. lancea*, with coefficients of variation (CVs) exceeding 0.6 for four essential oils. Integrating metabolomics with other omics approaches has proven to be beneficial in identifying genes of interest [22]. Given its chemical diversity, *A. lancea* represents an optimal model organism to study the genetic underpinnings of metabolite accumulation and its subsequent regulation.

*Atractylodes lancea* shows natural variation in medicinal quality traits, primarily determined by four essential oils: atractylodin, atractylon, β-eudesmol, and hinesol. In this study, GWAS was conducted using a collection of 130 wild accessions with various variant types. Through the analysis of epigenetic modifications and gene mutations related to environmental stress, we found that environmental factors significantly influence the formation of Dao-di herbs, and the continuity of spatiotemporal factors leads to continuous genetic and phenotypic changes. Remarkably, the geographic distribution and phenotypic variations of *A. lancea* showed marked differences in medicinal quality traits, such as β-eudesmol and atractylodin contents. Through our GWAS analysis, we identified 10 genes related to quality traits, including the UDP-glucose flavonoid 3-*O*-glucosyltransferase gene (*AlUGF3OG6*) related to β-eudesmol and the zinc finger protein gene (*AlZFP706*) associated with atractylodin. Previous studies on Tartary buckwheat domestication revealed that emodin content is related to the transcription levels of UGT genes [15]. Similarly, Peng *et al*. [47] demonstrated that differentially evolved glucosyltransferases play a crucial role in determining natural variation associated with flavone accumulation and the resulting UV tolerance in rice. In our study, we hypothesized that the *AlUGF3OG6* gene associated with β-eudesmol content may be influenced by epigenetic modifications, specifically glycosylation. Additionally, our GWAS analysis identified a potential relationship between *AlZFP706* and changes in atractylodin content. The zinc finger proteins (ZFPs) play crucial roles in environmental stress responses, such as drought stress responses. ZFPs can be categorized into four classes via conserved cysteine (C) and histidine (H) residues, namely C6, C2C2, C2H2, and C3HC4. *Arabidopsis thaliana AtPLATZ1* and *AtPLATZ5* have been previously reported to regulate stress tolerance. Studies have shown that germination and cotyledon development are delayed by overexpressing soybean *GmPLATZ1* in *Arabidopsis* under osmotic stress and ABA treatment [48]. The zinc finger TF ZAT5 induced several miRNAs and protein-coding transcripts, thus influencing drought tolerance in apple [49]. The *ZAT* gene family belongs to the plant-specific *C2H2* gene family. Previous research has suggested the diverse functions of *ZATs *in drought stress, oxidative stress, salt stress, and cold stress in *Arabidopsis* [50]. Importantly, we found that the *AlAAHY1* gene is significantly correlated with the content of hinesol. We also indirectly confirmed the function of this gene using tissue-cultured seedlings and hairy roots of *A. lancea*. The results showed that hinesol content increased significantly in both hairy roots and tissue-cultured seedlings of *A. lancea* under ABA treatment, suggesting a pivotal role of hinesol in plant protection under adverse conditions. In plant protection, the *AlAAHY1* gene participates in the regulation of the changes in hinesol content. Previous studies have indicated that the ABA 8-hydroxylase protein might be an important functional protein in potato tuber dormancy release and germination under heat stress [51]. A recent work indicates that the expression of the ABA 8′-hydroxylase gene is regulated by variations in proximal DNA sequences and its transcript level is negatively correlated with plant drought resistance in *Zea* [52]. Therefore, we hypothesize that the *AlAAHY1* gene is very important in regulating the content of hinesol in *A. lancea* and responses to high-temperature and drought stress.

Various external stimuli, such as light, climate, and soil conditions, have continuous effects on plant growth and development, leading to specific gene expression responses in plants. TFs serve as important control switches for regulating gene expression and can influence numerous biological processes at the transcriptional level in plants. Medicinal plants, as they adapt to different growth environments over time, develop unique properties. Recently, significant progress has been made in studying transcriptional regulation in various medicinal plants, especially with the emergence of new cultivation models in traditional Chinese medicine, such as the ecological agriculture of Chinese Materia Medica and the simulated cultivation of medicinal plants. These studies have provided insight into the mechanisms by which medicinal plants adapt to environmental stress and how such stress leads to the generation of medicinal material quality. The synthesis of bioactive components and transcriptional regulation are often complex processes and involve multiple layers in medicinal plants. As a result, TF families have received much attention and have been extensively studied. Numerous reports have highlighted the importance of the MYB, AP2/ERF, bHLH, WRKY, and bZIP TF families. These TFs are crucial components of signaling networks that regulate various biological processes in plants. In the present study, we analyzed the selective sweep according to different distribution areas of *A. lancea*. This analysis allowed us to identify several TFs significantly associated with the content of atractylodin, a compound closely related to the natural variation and high-quality formation of *A. lancea*. Among the identified TFs, some include heat stress TFs *TGA7* and *MYB39*, transcription initiation factor *TFIID*, trihelix TF *ASIL2*, transcription repressor *KAN1*, and a *GATA* TF. These TFs are important in the microevolutionary study of *A. lancea* quality formation under environmental stress. However, further functional validation of these genes is necessary to deepen our understanding of traits associated with quality formation.

## Materials and methods

### Genome assembly

#### DNA extraction and library preparation

*Atractylodes lancea* was originally collected from the Tangshan town, Nanjing City, China (32.06°N, 119.02°E, 121.6 m asl). DNA was extracted from *A. lancea* young leaves for Illumina paired-end sequencing. Genomic DNA extraction was conducted utilizing a DNasecure Plant Kit (Tiangen Biotech, Beijing, China). With an insert size of 350 bp, sequencing libraries were prepared according to the protocols provided by Illumina Inc. (San Diego, CA, USA), and then sequenced using the HiSeq X platform (Illumina Inc.).

Additionally, 10 μg sheared DNA was used to construct a library with an insert size of 20 kb. The SMRTbell™ template was used to measure DNA expression and perform end repair and hairpin adapter ligation. Finally, 20-kb PacBio single-molecule real-time (SMRT) DNA sequencing was performed at Pacific Biosciences, Inc., Menlo Park, CA, USA.

The Hi-C library was prepared using DNA from the same plant samples as mentioned above. After processing the collected leaf samples, the leaf cells were lysed and chromatin was digested with Hind endonuclease. The 5′ end of the DNA was obtained using labeled nucleotides and then ligating the blunt ends with T4 DNA ligase. After protease digestion, the obtained pure DNA was cleaved and the linker was ligated to the DNA [53]. We extracted the biotin-labeled fragments for subsequent PCR enrichment, followed by sequencing using the Illumina HiSeq X.

#### Estimation of genome size

Sequencing data underwent *k*-mer frequency analysis to identify genomic features [54]. Based on the *k*-mer (*k* = 17) result, the genome size of the sampled plants was evaluated using the optimized Lander–Waterman method. We estimated the genome size using the following formula: (*N* × (*L* − *k* + 1) − *B*)/*D* = *G*, where *N* is the total number of sequence reads, *L* is the average length of sequence reads, *K* is the *K*-mer length (17 bp) [55], *B* is the total number of low-frequency *K*-mers (frequency ≤ 1 in this analysis), *G* is the genome size, and *D* is the overall depth, estimated via the *K*-mer distribution. The heterozygosity is obtained by determining characteristics based on the quantity distribution of each *k*-polymer (*k* = 17).

#### Genome assembly

The FALCON method was employed for *de novo* genome assembly [56]. To generate enough corrected reads, the longest coverage of subreads was first selected as seed reads to correct sequence errors. The corrected reads were aligned and assembled into genomic contigs. After the initial assembly, we generated primary contigs (*p*-contigs) using FALCON-Unzip coupled with a modification using Quiver [57]. Sequence errors were correcting using the Pilon method. We compared the Hi-C data with assembled scaffolds. Furthermore, we clustered the scaffolds using the BWA-MEM algorithm and LACHESIS, to provide support for subsequent analysis.

The CEGMA pipeline was applied to assess assembly completeness [58]. When evaluating the genome, the BUSCO method was selected [59], and 1440 single-copy orthologous genes were identified. After obtaining the reads of the short insertion size paired-end library that met the requirements, the Hi-C sequencing data was mapped into genome assembly using the BWA-MEM algorithm method, and the assembly effect was evaluated based on the obtained results. The sequencing depth distribution at each location was determined based on the SAM tools method, and integrity of assembly was evaluated using the obtained results.

### Genome characterization and annotation

#### Annotation of repetitive sequences

By combined *de novo*-based and homology-based approaches, TEs in the *A. lancea* genome were annotated. We used RepeatModeler [60] and RepeatScout, as well as LTR_FINDER [61], to obtain a library for *de novo* repeats. Additionally, we employed the homology-based approach using RepeatProteinMask in the TE protein database and RepeatMasker 3.3.0 in the Repbase TE library [62].

#### Identification of protein-coding genes

The PCGs in the *A. lancea* plant genome were studied using a combination of different prediction methods, mainly including *de novo* methods for prediction, transcriptome, and searching based on homology, to enhance the accuracy of the results obtained. We downloaded homologous proteins of eight plant genomes (*Arabidopsis thaliana*, *Artemisia annua*, *Cynara cardunculus*, *Chrysanthemum nankingense*, *Lactuca sativa*, *Helianthus annuus*, *Mikania micrantha*, and *Taraxacum kok-saghyz*) from the NCBI website. Then, we employed TBLASTN to compare and analyze protein sequences [63]. The comparison was conducted using a combination of BLAST integrated in the Solar software with the threshold E-value set at 1e−5 [64]. Using GeneWise, we predicted gene structures in each BLAST hit (Homo set) [65]. During transcriptional analysis, reads from RNA sequencing were mapped using TopHat and Cufflinks 2.1.1 [66, 67], and assembled using the Trinity assembly and analysis pipeline. The result was used to construct relevant pseudo-unigenes, which underwent mapping and prediction using PASA [68]. This collection of genes was referred to as the PASA-T set, which served as the training set for the following *ab initio* models: Augustus (2.5.5), SNAP, GENSCAN (1.0), GeneID, and GlimmerHMM (3.0.1) [69–72]. To generate a non-redundant gene structure set, gene model evidence from various sources was integrated using EVidenceModeler (EVM) [73]. The different sources included the Homo set, Cufflinks set, PASA-T set, and *ab initio* programs.

#### Functional annotation

PCGs underwent functional annotation via BLASTP with E-value set at 1e−05 in NR databases and Swiss-Prot [74]. We further annotated protein domains using HMMER (V3.1) and InterProScan (V4.8) to search in the Pfam (V27.0) and InterPro (V32.0) databases, respectively [75–78]. After searching for InterPro entries, the database of GO terms for each gene was constructed. In addition, the BLAST tool was used to configure KEGG pathways (E-value <1e−05).

#### Annotation of non-coding RNAs

Non-coding RNA (ncRNA) annotation involved identifying tRNA genes using the tRNAscan-SE package [79]. Prediction of rRNA fragments was conducted using BLASTN (E-value <1e−10). Alignment against the Rfam database using the INFERNAL tool was employed to predict miRNAs and snRNAs [80, 81].

### Genome evolution

#### Phylogenetic analysis

The protein sequences underwent filtering to ensure accurate analysis: (i) in cases where multiple transcripts existed within a single gene, only the longest transcript was retained for further examination, and (ii) proteins below 30 amino acids in length were excluded. Alignment between proteins across 12 species was performed, employing a threshold E-value <1e−5. Subsequently, the protein sequences were clustered into paralogs and orthologs using the program OrthoMCL with an inflation value of 1.5. Following clustering, protein sequences were aligned from 191 single-copy genes, and the outcomes were amalgamated into a super-alignment matrix [82]. We constructed the phylogenetic tree encompassing the 12 species via maximum likelihood with 100 bootstrap replicates using RAxML [83]. Finally, the MCMCTree program was used for divergence time [84]. With specific parameters, the MCMCTree was configured: a burn-in at 10000, and sample size and frequency at 100 000 and 2, respectively. The divergence calibration time between *S. tuberosum* and *M. domestica* was 111.0–131.0 Mya, between *D. carota* and *S. tuberosum* it was 95.0–106.0 Mya, between *D. carota* and *H. annuus* it was 77.3–91.7 Mya, and between *A. annua* and *H. annuus* it was 31.6–36.9 Mya, according to the TimeTree database.

#### Analysis of gene families expansion and contraction

In this study, alterations in gene family sizes across ancestral and individual species were assessed through the utilization of CAFÉ software [85]. Changes in gene families were evaluated employing a random birth and death model, and the results of each lineage were compared. Then, the probability graph model (PGM) was applied to estimate the likelihood of transformation for each gene family at different nodes. Conditional likelihood was used to conduct statistical tests and the relevant *P*-values in every lineage were determined by substituting data to perform calculations. Gene families demonstrating significant expansion or contraction were identified based on a predefined threshold of conditional *P*-value <0.05.

#### Positively selected genes

To determine positively selected genes in *A. lancea* compared with the four related species, we aligned the single-copy genes via MUSCLE [86]. With *A. lancea* as the foreground branch, we then used the likelihood ratio tests (LRTs) of PAML to detect positively selected genes. *P*-values were determined based on χ^2^ and adjusted by using the false discovery rate (FDR).

#### Genome synteny and whole-genome duplication

Sequences from *A. lancea*, *C. cardunculus*, *H. annuus*, *L. sativa*, and *V. vinifera* were subjected to self-BLASTp searches (E-value <1e−5) to determine syntenic blocks [87]. The resulting data were then analyzed using MCScanX (−a, −e:1e−5, −u:1, −s:5) to delineate syntenic blocks, considering blocks with more than five genes. Then, the *K*_s_ values for syntenic segments were determined and their distribution was visualized.

### Population analysis

#### Library preparation and sequencing

Each sample utilized exactly 1.5 μg of genomic DNA for the DNA sample preparation process. Following the guidelines provided by Illumina Inc., the Truseq Nano DNA HT sample preparation kit was used to create a sequencing library, with unique index codes assigned to each sample. Initially, the genomic DNA underwent ultrasonic shearing to attain a fragment size of ~350 bp. Subsequent steps involved blunting the DNA fragment ends, A-tailing, and ligating them to adapters suitable for Illumina. This was followed by PCR amplification of the ligated fragments. Purification of the PCR products was carried out using AMPure XP from Beckman Coulter Inc., USA. The library size distribution was analyzed using an Agilent 2100 Bioanalyzer manufactured by Agilent Technologies Inc., USA.

The libraries, once prepared, underwent sequencing on the Illumina HiSeq X Ten platform. Next, the fastq format raw reads were subjected to a comprehensive quality control regimen to ensure data integrity. This process involved the removal of low-quality reads, identified as those containing ≥10% unidentified nucleotides, with >10 adaptor-aligned nucleotides, allowing for a maximum of 10% mismatches, and with >50% bases exhibiting a Phred quality score <5. Additionally, putative PCR duplicates were eliminated.

#### SNP calling and functional annotation

After filtering for quality, the remaining paired-end reads underwent alignment using the BWA-MEM algorithm 0.7.8, employing the command mem -t 4 -k 32 –M. To mitigate potential mismatches, putative PCR duplicates were eliminated using SAM tools. Following alignment, SNPs were called using GATK (Poplin *et al*., 2018). Only SNPs meeting stringent criteria with coverage depth >8, minor allele frequency >0.05, and missing data <0.1 were kept for following analyses.

SNP was annotated utilizing ANNOVAR, utilizing the *A. lancea* genome as reference. Annotation results categorized SNPs into various regions, including exons, introns, sites within 2 bp of a splicing junction, 1 kb up- and downstream of the transcription start site, as well as intergenic regions. Those residing in exons present as synonymous and non-synonymous groups based on whether they modify the encoded amino acid and alter the structure and function of the protein. They also include mutations that cause cessation or reduction of growth.

#### Phylogenetic tree and population structure

To explore genomic perspectives of phylogenetic relationships, we created a neighbor-joining tree utilizing the raxmlHPC-PTHREADS command within the RAxML (v 8.0.19) software, with 100 bootstrap iterations for robustness assessment [16]. ADMIXTURE v1.23 software was used to determine population genetic structure via the implemented expectation maximization algorithm. In addition, PCA, implemented in the GCTA software, was performed on these structures.

#### Selective sweep

VCFtools software was used to determine the fixation index (*F*_ST_) and genetic diversity using a 50-kb window and 10-kb increment. Windows with *F*_ST_ values and log_2_(θπ ratio) in the top 5% served as potential outliers indicative of strong selective sweeps. Finally, Pixy software was also used to evaluate nucleotide diversity and genetic divergence (*F*_ST_, π, and *d*_xy_) among *Atractylodes* species using a sliding window method (100-kb window) [88]. Furthermore, Pixy software was utilized to analyze nucleotide diversity and genetic divergence (*F*_ST_ and *d*_xy_) among *Atractylodes* species using a sliding window method (100-kb window), and all sites (variant and invariant sites) VCF as the input file [88].

#### Metabolite profiling and metabolomics

The ALR samples were collected, weighed, and promptly stored in liquid nitrogen. Just before analysis, the samples were thawed and ground. Subsequently, ~500 mg of material was carefully added to a 20-ml solid-phase microextraction (SPME) headspace vial. To prevent enzyme-catalyzed reactions, the solution was saturated with NaCl. Crimp-top caps were employed specifically to close the SPME vials with TFE-silicone headspace septa. Subsequently, each vial underwent a 5-min incubation at 60°C. Following this, the headspace of the sample was subjected to SPME analysis by exposing it to a 120-μm DVB fiber tip for 15 min with a temperature of 100°C. All consumables were provided by Agilent Technologies Inc.

Upon sample collection, the metabolites were desorbed from the fiber coating at 250°C for a duration of 300 s within the injection port of an Agilent 8890 GC system, produced by Agilent Technologies Inc. Following this, the metabolites were identified and quantitatively analyzed using the Agilent 8890 GC together with another GC–MS also from Agilent. This system featured a DB-5MS capillary column, with helium as the carrier gas at a flow rate of 1.2 ml/min. The heating protocol consisted of an initial period at 40°C for 210 s, followed by a 6-min gradual increase to 100°C, then increasing at 7°C per min until 180°C, and than a further 4-min gradual increase to 280°C, which was maintained for 5 min. The mass spectrometer operated in selected ion monitoring (SIM) mode for both the identification and quantification of analytes. The mass spectra were obtained utilizing electron impact (EI) ionization mode at an energy level of 70 eV. Temperatures were set to maintain the ion source, quadrupole mass detector, and transfer line at 230, 150, and 280°C, respectively.

#### Quantitative analysis of essential oils in *A. lancea* rhizomes

Initially, the ALR samples were subjected to grinding and subsequent filtration through a mesh size of 60 (Jun *et al*., 2018). Five hundred micrograms of material was then introduced into a 50-ml centrifuge tube, and 20 ml of hexane was added to facilitate extraction. Following this, ultrasonication was conducted at a frequency of 40 kHz for a duration of 30 min. Subsequently, the supernatant was removed by centrifugation at 3000 rpm for 10 min at 25°C. The residue underwent another round of extraction using an additional 20 ml of hexane. The resulting extracted solutions were then combined and adjusted to 50 ml with hexane before undergoing filtration.

For the GC analysis, a 1-μl aliquot from each sample was injected into a Thermo Trace 1300 GC system, integrating a DB-5MS column with a particle size of 0.25 μm. Injection in split-flow mode achieved a split ratio of 50:1. The carrier gas (helium) flow rate was 1 ml/min. The column was heated initially to 120°C for 2 min, followed by a 24-min gradual increase to 240°C, with a final hold time of 5 min. GC–MS analysis was performed in EI mode with energy level 70 eV, scanning a mass range from *m*/*z* 40 to 500 to capture spectral data.

#### Identification of key metabolites

Analysis of differentially expressed metabolites using the WGCNA R software package enabled the construction of a coexpression network [89]. Initially, sample clustering was conducted to identify any potential outliers. Subsequently, the metabolite coexpression network was established through WGCNA, with the determination of the soft threshold power and calculation of adjacency. Hierarchical clustering and dynamic tree cutting methods were then applied for module detection. Furthermore, the relationship between metabolite significance and module membership was assessed to associate the modules with the four essential oil traits of *A. lancea*. Relevant information regarding module metabolites was extracted for further examination. Using the ggplot2 R package, visual representations of the variations in core hub metabolites were generated.

#### Genome-wide association study analysis

In this study, a comprehensive genome-wide association study (GWAS) was undertaken utilizing data pertaining to 1 952 561 common SNPs with missing rate ≤0.1, minor allele frequency ≥0.05, and depth ≥6. The association was analyzed employing GEMMA. We performed multivariate linear model (MLM) analysis as follows: *y* = *X*α + Sβ + Kμ + *e*. Here, *y* represents the phenotype, *X* denotes the genotype, and S and K represent the structure matrix and relative kinship matrix, respectively. Additionally, *X*α and Sβ represent fixed effects, while Kμ and *e* stand for random effects. For population structure correction, the first three principal components were utilized to generate the S matrix, and this coefficient matrix was employed to determine the K matrix. GEMMA software was employed to conduct the above-mentioned analyses [90].

#### Expression of 10 key candidate genes

Three biological replicates of 100 mg *A. lancea* tissue-culture seedling (roots and leaves) was utilized to obtain total RNA using the RNAprep Plant Kit from Ark Biotechnology, RZ316-02, China. Ten key candidate genes associated with natural variations of *A. lancea* medicinal quality traits were applied to analyze gene expression patterns. The primer sequences of the 10 genes and 1 internal reference gene (β-actin) are shown in Supplementary Data Table S30. Among these, the relative transcript levels of *AlAAHY1* in *A. lancea* tissue-culture seedlings (roots and leaves) treated with 100 μM ABA and non-treated samples were also studied. Relative gene expression levels in different samples were detected through fluorescent quantitative PCR. The RT–qPCR procedure was 95°C for 2 min and 40 cycles of 55°C for 30–34 s. Each sample was used in triple technical replicates. The relative transcript levels were determined via the 2^−ΔΔCT^ approach. Primers applied in the present study are shown in Supplementary Data Table S30.

#### Functional validation of the *AlAAHY1* gene associated with hinesol accumulation in *A. lancea*

*Atractylodes lancea* hairy roots and tissue-culture seedlings were treated with 100 μM of ABA (batch number PH109X; Beijing Bioengineering Co., Ltd.). The chemical content of the tissue-culture seedling roots and hairy roots was measured after treating the test samples (*A. lancea* tissue culture seedlings and hairy roots) with 100 μM of ABA for 30 days, with six biological repeats for each group of samples. The *A. lancea* tissue-culture seedlings and hairy roots were cultured on a specific medium, namely basal (MS)-agar medium with 30 g l^−1^ sucrose. The hinesol reference compound was purchased from Shanghai Yuanye Company (Shanghai, China), and the chemical content measurement method used was described in ‘Quantitative analysis of *A. lancea* rhizomes essential oils’ of Materials and methods.. The method of inducing *A. lancea* hairy roots was that previously proposed (Zhang *et al*., 2023), and the materials for the tissue culture seedlings and hairy roots experiment were all preserved by our research group from an earlier period.

## Supplementary Material

Web_Material_uhae167

## Data Availability

The original contributions presented in this study are included in the article and its supplementary material. Further inquiries can be directed to the corresponding authors.
