# Supplementary material for: Genome resequencing reveals the genetic basis of population evolution, local adaptation, and rewiring of the rhizome metabolome in Atractylodes lancea
Source: Hortic Res. 2024 Jun 21;11(8):uhae167. doi: 10.1093/hr/uhae167 (PMC11300843; doi:10.1093/hr/uhae167)
Supplement: Web_Material_uhae167 [file web_material_uhae167.zip › Supplementary table.docx]

**Supplemental Table1. 17 K-mer statistics**

| K-mer | K-mer number | K-mer Depth | Genome Side (Mbp) | Revised Genome Size(Mb) | Heterozygous Ratio (%) | Repeat (%) |
| --- | --- | --- | --- | --- | --- | --- |
| 17 | 451, 351, 881, 180 | 108 | 4, 179.18 | 4, 162.72 | 1.65 | 82.78 |

**Supplemental Table2. The sequencing data of *A. lancea***

| Pair-end libraries | Inser size | Total data (G) | Read length (bp) | Sequence coverage (X) |
| --- | --- | --- | --- | --- |
| Illumina reads | 350 bp | 589.90 | 150 | 141.71 |
| PacBio reads | － | 580.84 | － | 139.53 |
| Hic | － | 639.98 | － |  |
| Total | － | 1170.74 | － | 281.24 |

**Supplemental Table3. The *de novo* assembly results of *A. lancea***

| Characteristic Length number | | | | |
| --- | --- | --- | --- | --- |
| Contig** (bp) Scaffold (bp) Contig** Scaffold | | | | |
| Total | 4, 008, 738, 633 | 4, 009, 192, 933 | 6, 220 | 1, 677 |
| Max | 8, 269,134 | 478, 768, 905 | － | － |
| Number＞＝2000 | － | － | 6, 220 | 1, 677 |
| N50 | 1, 183, 812 | 289, 329, 680 | 1, 008 | 6 |
| N60 | 946, 395 | 257, 869, 696 | 1, 387 | 7 |
| N70 | 734, 118 | 246, 359, 527 | 1, 866 | 9 |
| N80 | 527, 647 | 213, 670, 615 | 2, 507 | 10 |
| N90 | 323, 391 | 191, 911, 171 | 3, 467 | 12 |

** contig after scaffolding

**Supplemental Table 4. The length of psedo-chrmosomes in genome of *A. lancea***

| Chrsomosome ID | Scaffolds/contigs | Length |
| --- | --- | --- |
| Chr0 | 729 | 478, 768, 905 |
| Chr1 | 595 | 441, 263, 290 |
| Chr2 | 571 | 413, 507, 591 |
| Chr3 | 411 | 338, 758, 869 |
| Chr4 | 245 | 221, 694, 797 |
| Chr5 | 374 | 289, 329, 680 |
| Chr6 | 303 | 257, 869, 696 |
| Chr7 | 296 | 253, 371, 345 |
| Chr8 | 268 | 246, 359, 527 |
| Chr9 | 231 | 213, 670, 615 |
| Chr10 | 228 | 206, 075, 337 |
| Chr11 | 304 | 283, 686, 006 |

**Supplemental Table5. Coverage statistics of genome of *A. lancea***

| Characteristic |  | Percentage (%) |
| --- | --- | --- |
| Read | Mapping rate (%) | 97.71 |
|  | Average sequencing depth | 118.54 |
|  | Coverage (%) | 99.65 |
| Genome | Coverage at least 4× (%) | 99.37 |
|  | Coverage at least 10× (%) | 99.00 |
|  | Coverage at least 20× (%) | 98.40 |

**Supplemental Table6. CEGMA evaluation results**

| Complete Complete+partial  #Prots %Completeness #Prots %Completeness | | | |
| --- | --- | --- | --- |
| 227 | 91.53 | 235 | 94.76 |

Complete: core gene that were completely assembled with identity greater than 70%.

Complete+partial: core genes that were assembled but not complete.

#Prots: the number of core genes.

%Completeness: percentage of assembled core genes to core genes.

**Supplemental Table7.** **BUSCO assessment of genome of *A. lancea***

| Species | BUSCO notation assessment results |
| --- | --- |
| *Atractylodes lancea* | C:89.9%[S:34.1%,D:55.8%],F:2.2%,M:7.9%,n:1440 |

#C: Complete Single-Copy BUSCOs; S: Complete and single-cppy BUSCOs; D: Complete Duplicated BUSCOs; F: Fragmented BUSCOs; M: Missing BUSCOs; n: Total BUSCO groups searched.

**Supplemental Table8.** **The statistics of repeat sequences in genome of *A. lancea***

| Type | Repeat Size (bp) | % of genome |
| --- | --- | --- |
| TRF | 206, 461, 077 | 5.15 |
| RepeatMasker | 2, 641, 555, 497 | 65.89 |
| RepeatProteinMask | 867, 138, 330 | 21.63 |
| Total | 2, 870, 472, 839 | 71.61 |

**Supplemental Table9.** **The statistics of tandem repeat elements in genome of *A. lancea***

| Type | Repbase+De novo Length(bp) | % in Genome | TE proteins Length (bp) | % in Genome | Combined TEs Length (bp) | %in Genome |
| --- | --- | --- | --- | --- | --- | --- |
| DNA | 150, 726, 473 | 3.76 | 62, 252, 070 | 1.55 | 199, 250, 875 | 4.97 |
| LINE | 18, 655, 456 | 0.47 | 39, 223, 165 | 0.98 | 53, 347, 455 | 1.33 |
| SINE | 1, 268, 129 | 0.03 | 0 | 0 | 1, 268, 129 | 0.031 |
| LTR | 2, 469, 843, 259 | 61.61 | 766, 453, 420 | 19.12 | 2, 549, 990, 086 | 63.61 |
| Simple repeat | 5, 680, 675 | 0.14 | 0 | 0 | 5, 680, 675 | 0.14 |
| Unknown | 18, 785, 648 | 0.47 | 9, 396 | 0.00023 | 18, 795, 044 | 0.47 |
| Total | 2, 641, 555, 497 | 65.89 | 867, 138, 330 | 21.63 | 2, 789, 748, 437 | 69.59 |

**Supplemental Table10.** **The statistics of gene structure in genome of *A. lancea***

| Methods | Gene set | Number | CDS+intron length (bp) | Average CDS length (bp) | Average exon length (bp) | Average intron length (bp) | Average exons per gene |
| --- | --- | --- | --- | --- | --- | --- | --- |
|  | Augustus | 127, 718 | 3, 146.82 | 768.98 | 235.01 | 1, 046.56 | 3.27 |
|  | GlimmerHMM | 252, 958 | 142, 97.59 | 449.82 | 155.61 | 7, 324.33 | 2.89 |
| *De novo* | SNAP | 249, 237 | 10, 243.12 | 538.57 | 161.27 | 4147.93 | 3.34 |
|  | Genscan | 187, 232 | 13, 512.34 | 819.44 | 171.92 | 3, 369.95 | 4.77 |
|  | Geneid | 294, 206 | 3, 679.18 | 497.98 | 165.62 | 1, 585.26 | 3.01 |
|  | Arabidopsis thaliana | 72, 034 | 2, 241.25 | 807.81 | 314.72 | 914.93 | 2.57 |
|  | Chrysanthemum  nankingense | 108, 273 | 1, 879.71 | 588.04 | 265.58 | 1, 063.82 | 2.21 |
|  | Cynara cardunculus | 230, 706 | 1, 952.6 | 963.54 | 463.97 | 918.57 | 2.08 |
|  | Lactuca sativa | 220, 354 | 1, 845.36 | 969.04 | 474.72 | 841.6 | 2.04 |
|  | Taraxacum kok saghyz | 241, 832 | 1, 366.92 | 730.76 | 422.66 | 872.73 | 1.73 |
|  | Helianthus annuus | 172, 437 | 2, 524.32 | 1, 196.64 | 526.2 | 1, 042.04 | 2.27 |
|  | Artemisia annua | 125, 657 | 4, 041.36 | 1, 392.21 | 593.91 | 1, 970.87 | 2.34 |
|  | Mikania micrantha | 98, 650 | 4, 772 | 1, 429.39 | 597.05 | 2, 397.69 | 2.39 |
| RNA-seq | Cufflinks | 82, 730 | 9, 704.69 | 1, 893.56 | 328.18 | 1, 637.57 | 5.77 |
|  | PASA | 38, 225 | 4, 515.89 | 997.95 | 243.11 | 1, 133.01 | 4.1 |
| EVM  PASA-updata  Final set | | 135, 901 | 3, 415.5 | 747 | 227.96 | 1, 171.99 | 3.28 |
|  |  | 135, 777 | 3, 413.18 | 747.04 | 228.39 | 1, 174.02 | 3.27 |
|  |  | 50, 658 | 5, 102.46 | 1, 004.59 | 221.26 | 1, 157.47 | 4.54 |

**Supplemental Table 11.** **The statistics of annotated gene in genome of *A. lancea***

| characters | Annotated Number | Annotated Percent(%) |
| --- | --- | --- |
| NR | 43, 636 | 86.1 |
| Swiss-Prot | 33, 868 | 66.9 |
| KEGG | 32, 310 | 63.8 |
| All | 45, 509 | 89.8 |
| InterProPfam | 39, 370 | 77.7 |
| GO | 33, 026 | 65.2 |
| Annotated | 48, 492 | 95.7 |
| Total | 50, 658 | － |

**Supplemental Table12.** **The statistics of non-coding RNA in genome *A. lancea***

| Type |  | Copy(w*) | Average length(bp) | Total length(bp) | %of genome |
| --- | --- | --- | --- | --- | --- |
| miRNA |  | 3, 441 | 121.7404824 | 418, 909 | 0.010450 |
| tRNA |  | 3, 121 | 71.90964434 | 224, 430 | 0.005599 |
|  | rRNA | 452 | 188.4911504 | 85, 198 | 0.002125 |
|  | 18S | 102 | 448.3823529 | 45, 735 | 0.001141 |
| rRNA | 28S | 95 | 129.9473684 | 12, 345 | 0.000308 |
|  | 5.8S | 25 | 140.32 | 3, 508 | 0.000088 |
|  | 5S | 230 | 102.6521739 | 23, 610 | 0.000589 |
|  | snRNA | 3, 635 | 110.6236589 | 402, 117 | 0.010031 |
| snRNA | CD-box | 3, 092 | 105.3851876 | 325, 851 | 0.008129 |
|  | HACA-box | 80 | 116.3625 | 9, 309 | 0.000232 |
|  | splicing | 462 | 144.487013 | 66, 753 | 0.001665 |

**Supplemental Table13.** **The genes used for gene family clustering in each specise**

| Species | Genes |
| --- | --- |
| *Artemisia annua* | 62, 285 |
| *Atractylodes lancea* | 50, 658 |
| *Coffea canephora* | 25, 574 |
| *Cynara cardunculus* | 26, 155 |
| *Chrysanthemum nankingense* | 56, 870 |
| *Daucus carota* | 32, 118 |
| *Helianthus annuus* | 58, 163 |
| *Lactuca sativa* | 34, 736 |
| *Malus domestica* | 47, 745 |
| *Mikania micrantha* | 46, 351 |
| *Salvia miltiorrhiza* | 27, 985 |
| *Solanum tuberosum* | 38, 953 |

**Supplemental Table14.** **The KEGG enrichment result of expanded gene families in *A. lancea***

| Map ID | Map Title | Pvalue | Adjusted Pv | Gene_Number |
| --- | --- | --- | --- | --- |
| map01040 | Biosynthesis of unsaturated fatty acids | 2.54E^-41^ | 2.24E^-39^ | 67 |
| map01212 | Fatty acid metabolism | 3.07E^-36^ | 1.35E^-34^ | 84 |
| map03430 | Mismatch repair | 1.25E^-26^ | 3.66E^-25^ | 53 |
| map03030 | DNA replication | 8.05E^-22^ | 1.77E^-20^ | 53 |
| map00909 | Sesquiterpenoid and triterpenoid biosynthesis | 2.10E^-21^ | 3.69E^-20^ | 37 |
| map03440 | Homologous recombination | 1.03E^-19^ | 1.51E^-18^ | 53 |
| map04144 | Endocytosis | 3.23E^-17^ | 4.06E^-16^ | 74 |
| map03420 | Nucleotide excision repair | 2.16E^-15^ | 2.38E^-14^ | 52 |
| map04141 | Protein processing in endoplasmic reticulum | 2.47E^-14^ | 2.42E^-13^ | 102 |
| map03040 | Spliceosome | 3.49E^-11^ | 3.07E^-10^ | 85 |
| map00908 | Zeatin biosynthesis | 8.87E^-08^ | 7.09E^-7^ | 20 |
| map00100 | Steroid biosynthesis | 7.78E^-7^ | 5.70E^-6^ | 23 |
| map00620 | Pyruvate metabolism | 4.98E^-5^ | 0.000337245 | 35 |
| map00230 | Purine metabolism | 7.36E^-5^ | 0.000462578 | 55 |
| map00240 | Pyrimidine metabolism | 0.000132994 | 0.000780233 | 43 |
| map03020 | RNA polymerase | 0.000165877 | 0.000912325 | 22 |
| map00071 | Fatty acid degradation | 0.000241881 | 0.001252087 | 20 |
| map00061 | Fatty acid biosynthesis | 0.003617289 | 0.017684524 | 17 |
| map00592 | alpha-Linolenic acid metabolism | 0.007883497 | 0.03651304 | 14 |
| map00052 | Galactose metabolism | 0.009022974 | 0.039701087 | 20 |

**P*＜0.05 by hypergeometric test and FDR adjustments.

**Supplemental Table15.** **The KEGG enrichment result of *A. lancea*-specific genes**

| Term | Datebase | AdjustedPv |
| --- | --- | --- |
| Homologous recombination | KEGG PATHWAY | 7.88E^-17^ |
| Mismatch repair | KEGG PATHWAY | 6.52E^-15^ |
| DNA replication | KEGG PATHWAY | 4.90E^-14^ |
| Endocytosis | KEGG PATHWAY | 1.06E^-8^ |
| Biosynthesis of amino acids | KEGG PATHWAY | 3.14E^-8^ |
| Selenocompound metabolism | KEGG PATHWAY | 3.90E^-7^ |
| Nucleotide excision repair | KEGG PATHWAY | 1.12E^-6^ |
| Phagosome | KEGG PATHWAY | 7.53E^-5^ |
| Protein processing in endoplasmic reticulum | KEGG PATHWAY | 8.02E^-5^ |
| Sesquiterpenoid and triterpenoid biosynthesis | KEGG PATHWAY | 0.620906829 |
| Terpenoid backbone biosynthesis | KEGG PATHWAY | 0.793119472 |

**P*＜0.05 by hypergeometric test and FDR adjustments.

**Supplemental Table16.** **The GO enrichment result of *A. lancea*-specific genes**

| GO_ID | GO_Term | GO_Class | AdjustedPv |
| --- | --- | --- | --- |
| GO:0008234 | cysteine-type peptidase activity | MF | 4.79E^-41^ |
| GO:0006508 | proteolysis | BP | 6.14E^-23^ |
| GO:0070011 | peptidase activity, acting on L-amino acid peptides | MF | 6.31E^-23^ |
| GO:0008233 | peptidase activity | MF | 9.88E^-22^ |
| GO:0016787 | hydrolase activity | MF | 2.06E^-6^ |
| GO:0009067 | aspartate family amino acid biosynthetic process | BP | 5.70E^-6^ |
| GO:0022836 | gated channel activity | MF | 0.00013635 |
| GO:0022839 | ion gated channel activity | MF | 0.00013635 |
| GO:0019538 | protein metabolic process | BP | 0.0015671 |
| GO:0016615 | malate dehydrogenase activity | MF | 0.0027171 |
| GO:0004470 | malic enzyme activity | MF | 0.0028336 |
| GO:0016619 | malate dehydrogenase (oxaloacetate-decarboxylating) activity | MF | 0.0028336 |
| GO:0003871 | 5-methyltetrahydropteroyltriglutamate-homocysteine S-methyltransferase activity | MF | 0.0028336 |
| GO:0042085 | 5-methyltetrahydropteroyltri-L-glutamate-dependent methyltransferase activity | MF | 0.0028336 |
| GO:0043648 | dicarboxylic acid metabolic process | BP | 0.0036047 |
| GO:0004970 | ionotropic glutamate receptor activity | MF | 0.0036279 |
| GO:0008066 | glutamate receptor activity | MF | 0.0036279 |
| GO:0008839 | 4-hydroxy-tetrahydrodipicolinate reductase | MF | 0.0052234 |
| GO:0008172 | S-methyltransferase activity | MF | 0.0052234 |
| GO:0005216 | ion channel activity | MF | 0.0052234 |
| GO:0022838 | substrate-specific channel activity | MF | 0.0052234 |
| GO:0005230 | extracellular ligand-gated ion channel activity | MF | 0.0057109 |
| GO:0005231 | excitatory extracellular ligand-gated ion channel activity | MF | 0.0057109 |
| GO:0009086 | methionine biosynthetic process | BP | 0.0060146 |
| GO:0000097 | sulfur amino acid biosynthetic process | BP | 0.0060146 |
| GO:0006555 | methionine metabolic process | BP | 0.0068473 |
| GO:0005261 | cation channel activity | MF | 0.0080785 |
| GO:0003854 | 3-beta-hydroxy-delta5-steroid dehydrogenase activity | MF | 0.0080785 |
| GO:0016229 | steroid dehydrogenase activity | MF | 0.0080785 |
| GO:0033764 | steroid dehydrogenase activity, acting on the CH-OH group of donors, NAD or NADP as acceptor | MF | 0.0080785 |
| GO:0015267 | channel activity | MF | 0.0091174 |
| GO:0022803 | passive transmembrane transporter activity | MF | 0.0091174 |
| GO:0022843 | voltage-gated cation channel activity | MF | 0.010057 |
| GO:0005234 | extracellular-glutamate-gated ion channel activity | MF | 0.01035 |

**P*＜0.05 by hypergeometric test and FDR adjustments.

**Supplemental Table17.** **Change rate of cross-validation (CV) error value of admixture in K-values ranged from 2 to 8**

| k | value |
| --- | --- |
| 7 | 0.29614 |
| 2 | 0.33579 |
| 3 | 0.31661 |
| 4 | 0.30466 |
| 5 | 0.3039 |
| 6 | 0.29117 |
| 8 | 0.28771 |

**Supplemental Table18. Statistics of genetic differences among *Atractylodes* species**

| Population | θπ (Pixy) |
| --- | --- |
| *A. coreana* | 6.19E-03 |
| *A. lancea* | 5.70E-03 |
| *A. japonica* | 4.77E-03 |
| *A. macrocephala* | 4.35E-03 |
| *A. carlinoides* | 2.39E-03 |

**Supplemental Table19. Statistics of genetic differences between population groups within *Atractylodes* species**

| Group A vs Group B | | Dxy (Pixy) | | | Fst (Pixy) | |
| --- | --- | --- | --- | --- | --- | --- |
| *A. carlinoides* | *A. macrocephala* | | 5.21E-03 | 0.47 | |  |
| *A. coreana* | *A. carlinoides* | | 5.46E-03 | 0.41 | |  |
| *A. coreana* | *A. macrocephala* | | 7.54E-03 | 0.40 | |  |
| *A. japonica* | *A. carlinoides* | | 5.23E-03 | 0.46 | |  |
| *A. japonica* | *A. coreana* | | 7.19E-03 | 0.30 | |  |
| *A. japonica* | *A. macrocephala* | | 7.00E-03 | 0.42 | |  |
| *A. lancea* | *A. carlinoides* | | 5.35E-03 | 0.43 | |  |
| *A. lancea* | *A. coreana* | | 7.32E-03 | 0.27 | |  |
| *A. lancea* | *A. japonica* | | 7.34E-03 | 0.38 | |  |
| *A. lancea* | *A. macrocephala* | | 7.63E-03 | 0.45 | |  |

**Supplemental Table20.** **Hub metabolites of the blue and brown modules**

| **Compounds** | **Class** | **Atractylon** | **Atractylodin** | **Hinesol** | **β-eudesmol** | **MMbrown** | **MMblue** |
| --- | --- | --- | --- | --- | --- | --- | --- |
| 1H-Pyrazolo[3,4-d]pyrimidin-4-amine | Heterocyclic compound | 0.577498136 | 0.54015498 | -0.401526164 | -0.30681437 | 0.701533723 | 0.845913909 |
| Carotol | Terpenoids | 0.518632418 | 0.479993531 | -0.583167029 | -0.496215801 | 0.722646324 | 0.845153444 |
| Naphthalene, 1,8-dimethyl- | Aromatics | 0.482644636 | 0.473865253 | -0.594679397 | -0.492301117 | 0.803054348 | 0.838558004 |
| Phenol, 4-(1,1,3,3-tetramethylbutyl)- | Phenol | 0.475692241 | 0.491783009 | -0.581430868 | -0.486678708 | 0.714057772 | 0.834209179 |
| Cedrol | Terpenoids | 0.469719311 | 0.42630973 | -0.595344221 | -0.500674701 | 0.719157987 | 0.811951863 |
| .gamma.-Elemene | Terpenoids | 0.464877031 | 0.444416343 | -0.592277096 | -0.498622166 | 0.708058216 | 0.809121047 |
| 1,9-Nonanedithiol | Sulfur compounds | 0.464820415 | 0.429061402 | -0.606435177 | -0.511393732 | 0.725008012 | 0.80837165 |
| N-Cyanomethyl-2-cyanimino-pyrrolidine | Heterocyclic compound | 0.456567195 | 0.431421963 | -0.621954594 | -0.529542615 | 0.804784158 | 0.803930536 |
| Diethyl Phthalate | Ester | 0.456211584 | 0.468594581 | -0.564098951 | -0.474154062 | 0.775610293 | 0.800184021 |
| Biuret, 1-[(dimethylamino)methylene]- | Nitrogen compounds | 0.452553007 | 0.451909431 | -0.610698615 | -0.521999033 | 0.706454108 | 0.789188319 |
| Butanoic acid, 2-methyl-, 3,7-dimethyl-2,6-octadienyl ester, (E)- | Ester | 0.450155047 | 0.436302425 | -0.600905906 | -0.510847207 | 0.749444056 | 0.784476482 |
| cis-.beta.-Farnesene | Terpenoids | 0.44469172 | 0.43765557 | -0.62731193 | -0.538858405 | 0.743594983 | 0.78429196 |
| Salvial-4(14)-en-1-one | Terpenoids | 0.439491113 | 0.430999104 | -0.615079468 | -0.524731726 | 0.734997776 | 0.784208977 |
| BenzAldehyde, 3,4-dihydroxy- | Aldehyde | 0.439437246 | 0.381735875 | -0.628626738 | -0.530754657 | 0.791394994 | 0.745950261 |
| Butanoic acid, 3-methyl-, 1-ethenyl-1,5-dimethyl-4-hexenyl ester | Ester | 0.411272749 | 0.457524237 | -0.622492517 | -0.538482182 | 0.910788225 | 0.71158387 |

Note: Hub metabolites were defined as having a metabolite significance over 0.2 and a module membership over 0.7.

**Supplemental Table21.** **Abbreviation statistics for the genome section of the article**

| Abbreviation | Full name |
| --- | --- |
| 4DTv | the fourfold degenerate site |
| B | bracts |
| BUSCO | benchmarking Universal Single-Copy Orthologs |
| CDS | coding sequence |
| CDSs | coding sequences |
| CEGMA | core eukaryotic gene mapping approach |
| CGs  CS | core eukaryotic genes  harvest stage |
| DAMs | differentially accumulated metabolites |
| DAMs | differentially accumulated metabolites |
| DEGs | differentially expressed genes |
| EVM | evidencemodeler |
| F | flowers |
| FDR | false discovery rate |
| FPP | famesyl pyrophosphate |
| GO | Gene Ontology |
| Hi-C | high-throughput chromosome conformation capture |
| HS-SPME | head space solid-phase microextraction |
| IPP | isopentenyl diphosphate |
| KEGG  KH | kyoto Encyclopedia of Genes and Genomes  flowering stage |
| L | leaves |
| LTR | long retrotransposon terminal repeats |
| miRNA | micro-RNA |
| MRCA  MEP  MVA | most recent common ancestor  2-C-methyl-D-erythritol 4-phosphate pathway  Mevalonate pathway |
| ncRNA | noncoding RNA |
| NR | non-redundant Protein Sequence Datebase |
| PCA | principal component analysis |
| PCGs | protein-coding genes |
| *p*-contigs | primary contigs |
| PCR | polymerase chain reaction |
| PE | paired-end |
| R  SZ | roots  vegetative growth |
| RH | rhizomes |
| S | stems |
| SMRT | single real-time |
| snRNA | small nuclear RNA |
| TEs | transposable elements |
| TPS | terpene synthase |
| VIP | variable importance of the Projection |
| WGD | whole-genome duplication |
