# Supplementary figures and images for: Genome resequencing reveals the genetic basis of population evolution, local adaptation, and rewiring of the rhizome metabolome in Atractylodes lancea
Source: Hortic Res. 2024 Jun 21;11(8):uhae167. doi: 10.1093/hr/uhae167 (PMC11300843; doi:10.1093/hr/uhae167)

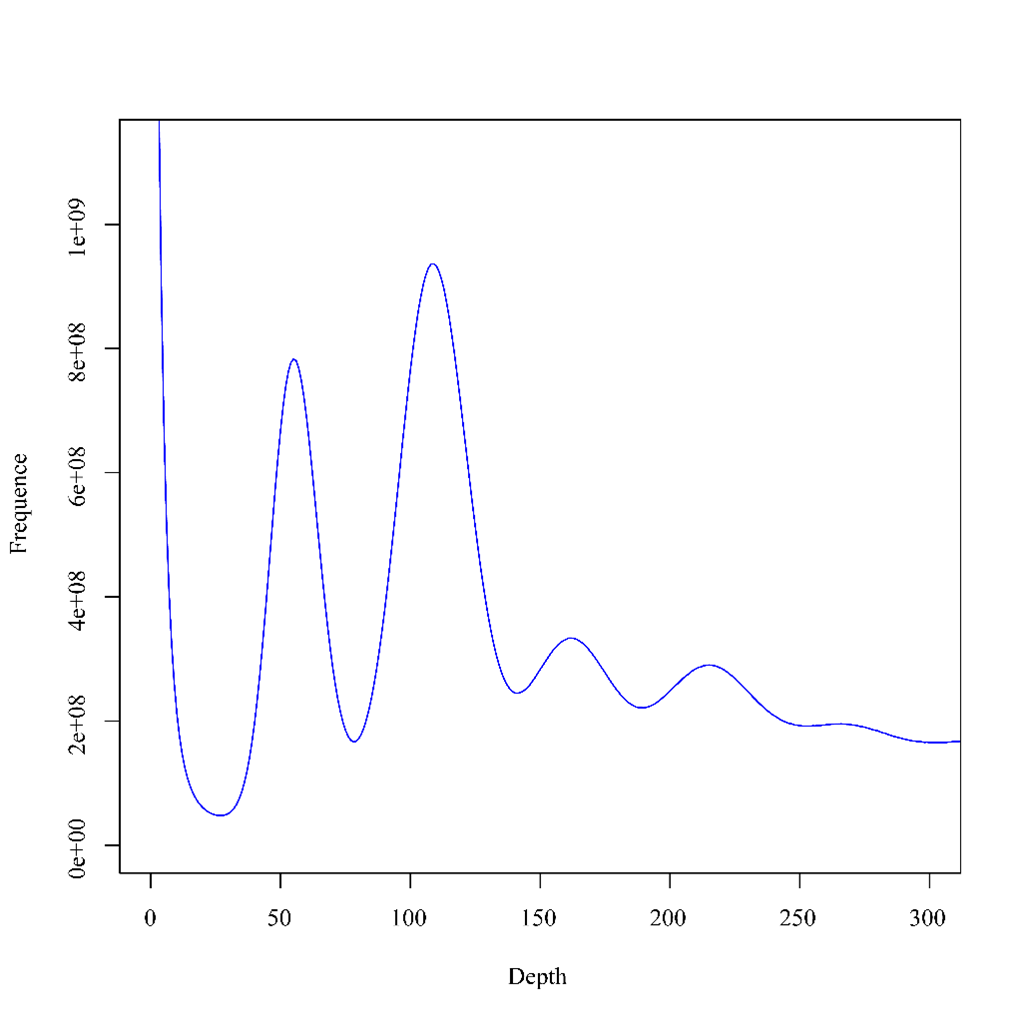

Supplement: Web_Material_uhae167 [file web_material_uhae167.zip › Figure S1.tif]

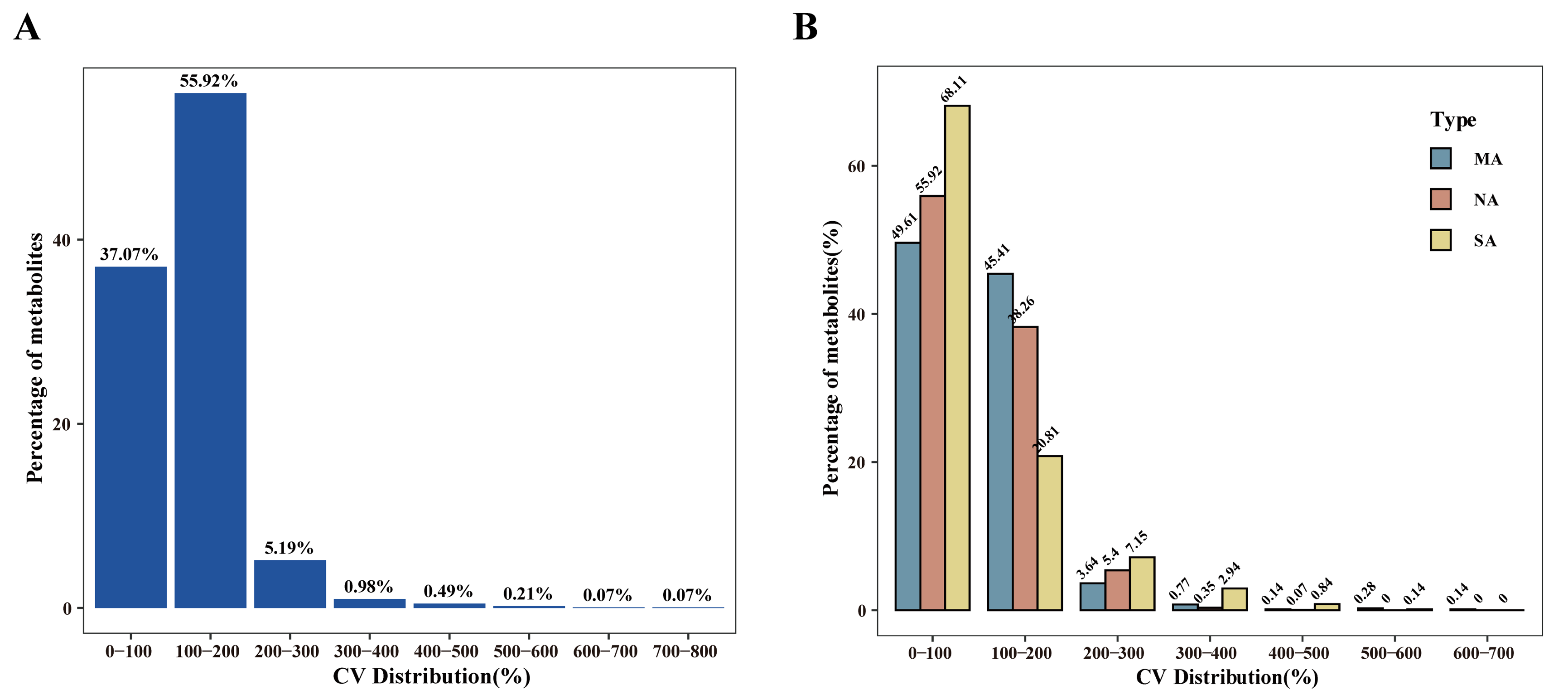

Supplement: Web_Material_uhae167 [file web_material_uhae167.zip › Figure S10.tif]

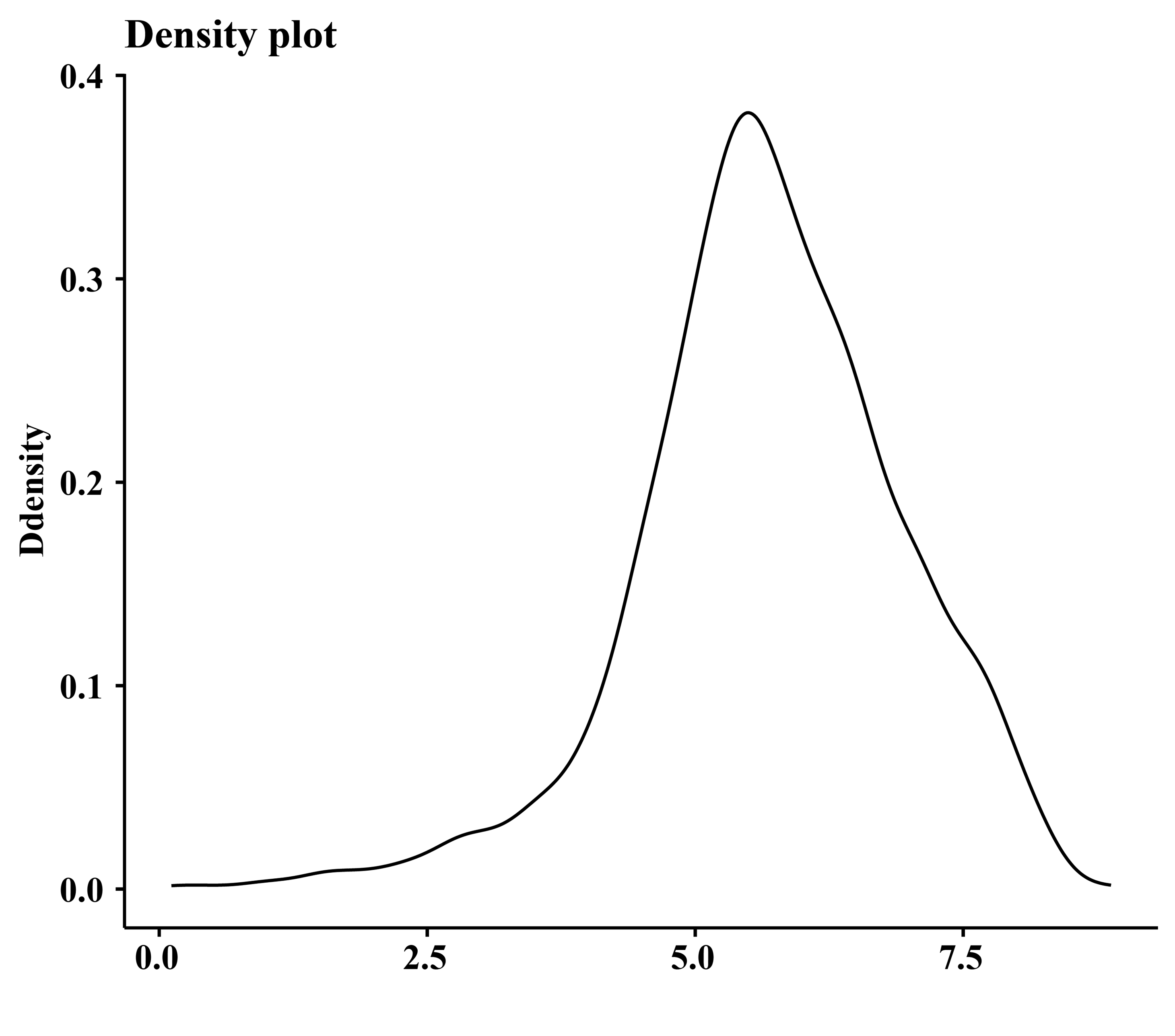

Supplement: Web_Material_uhae167 [file web_material_uhae167.zip › Figure S11.tif]

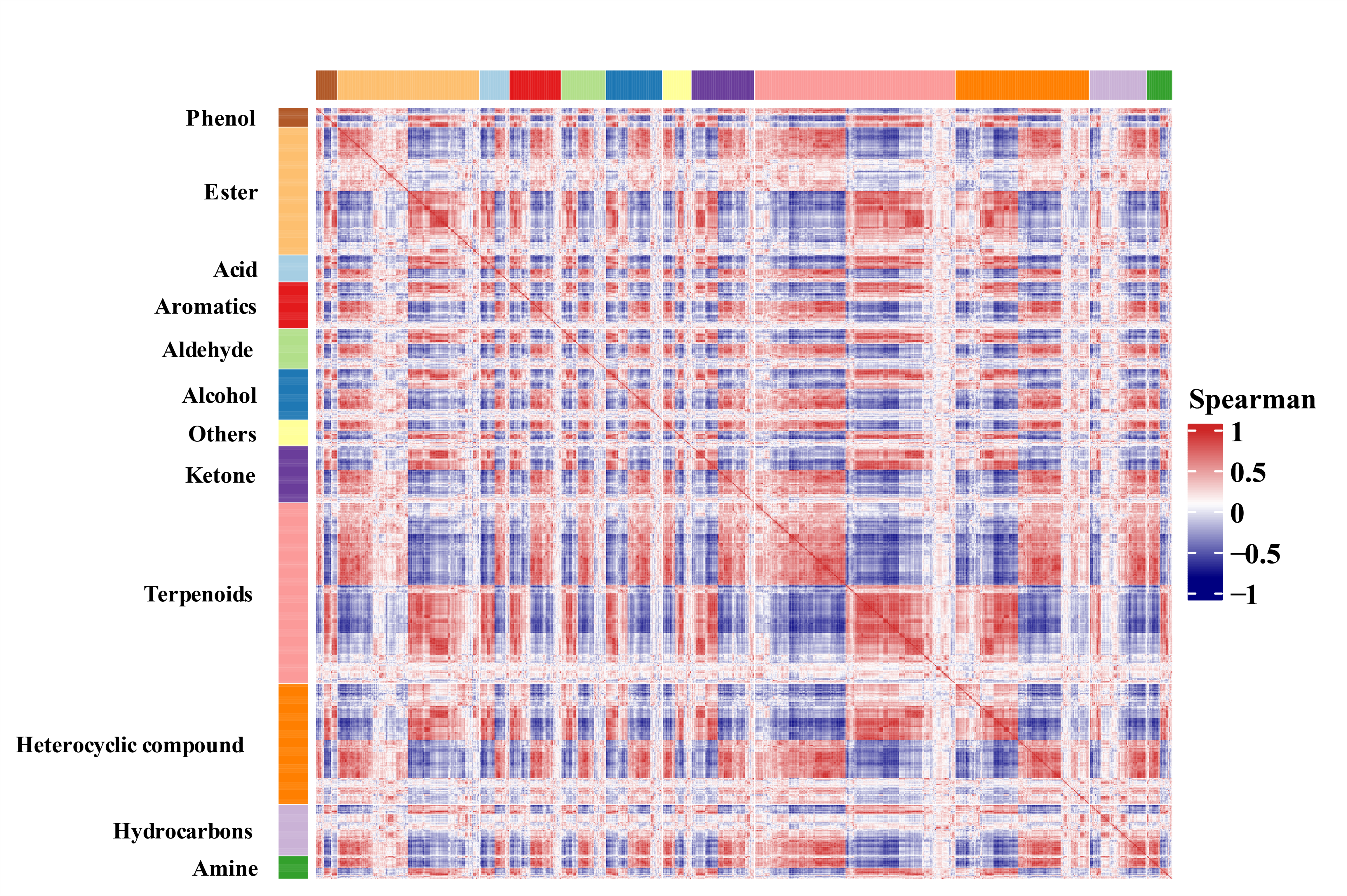

Supplement: Web_Material_uhae167 [file web_material_uhae167.zip › Figure S12.tif]

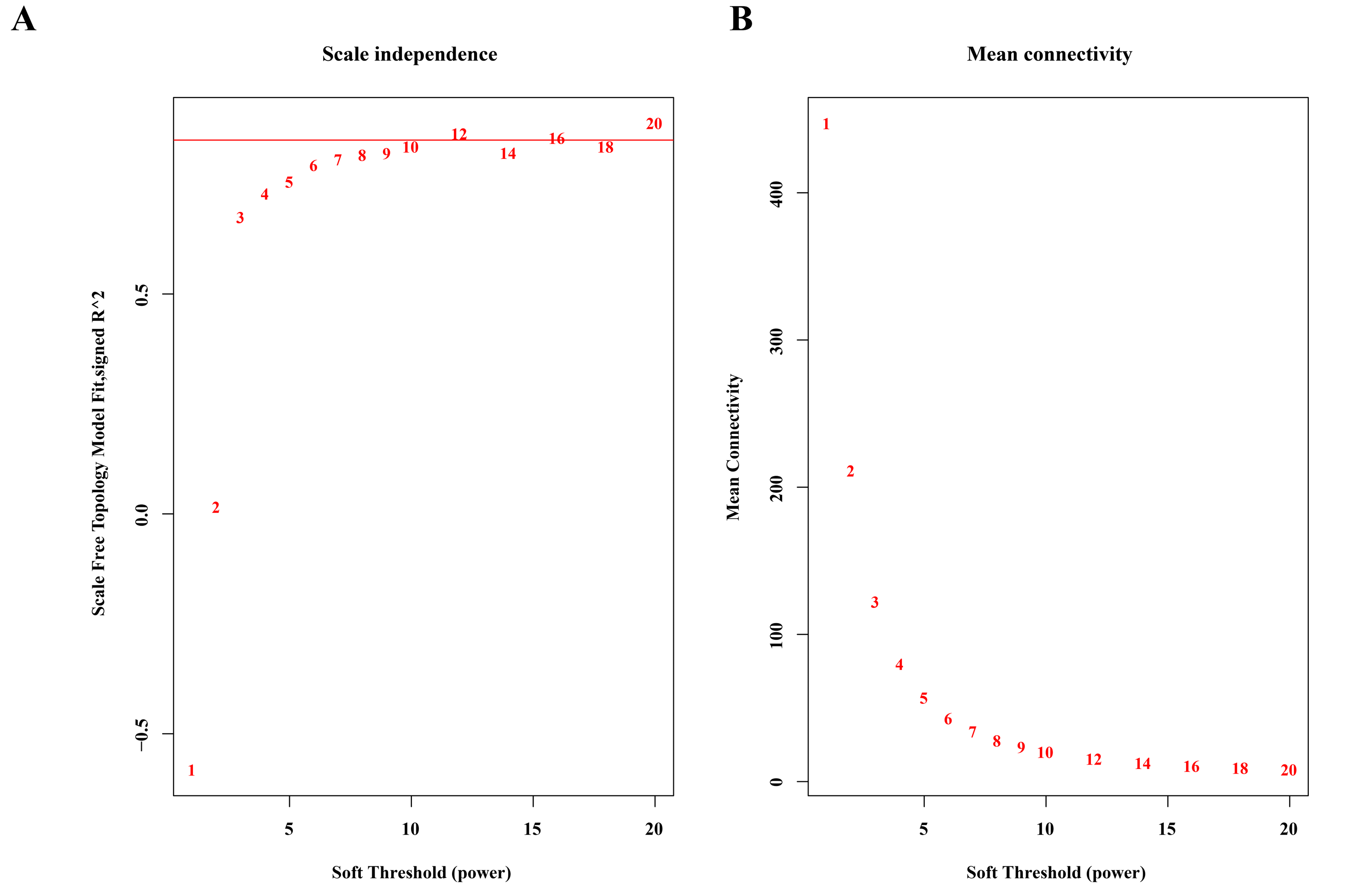

Supplement: Web_Material_uhae167 [file web_material_uhae167.zip › Figure S13.tif]

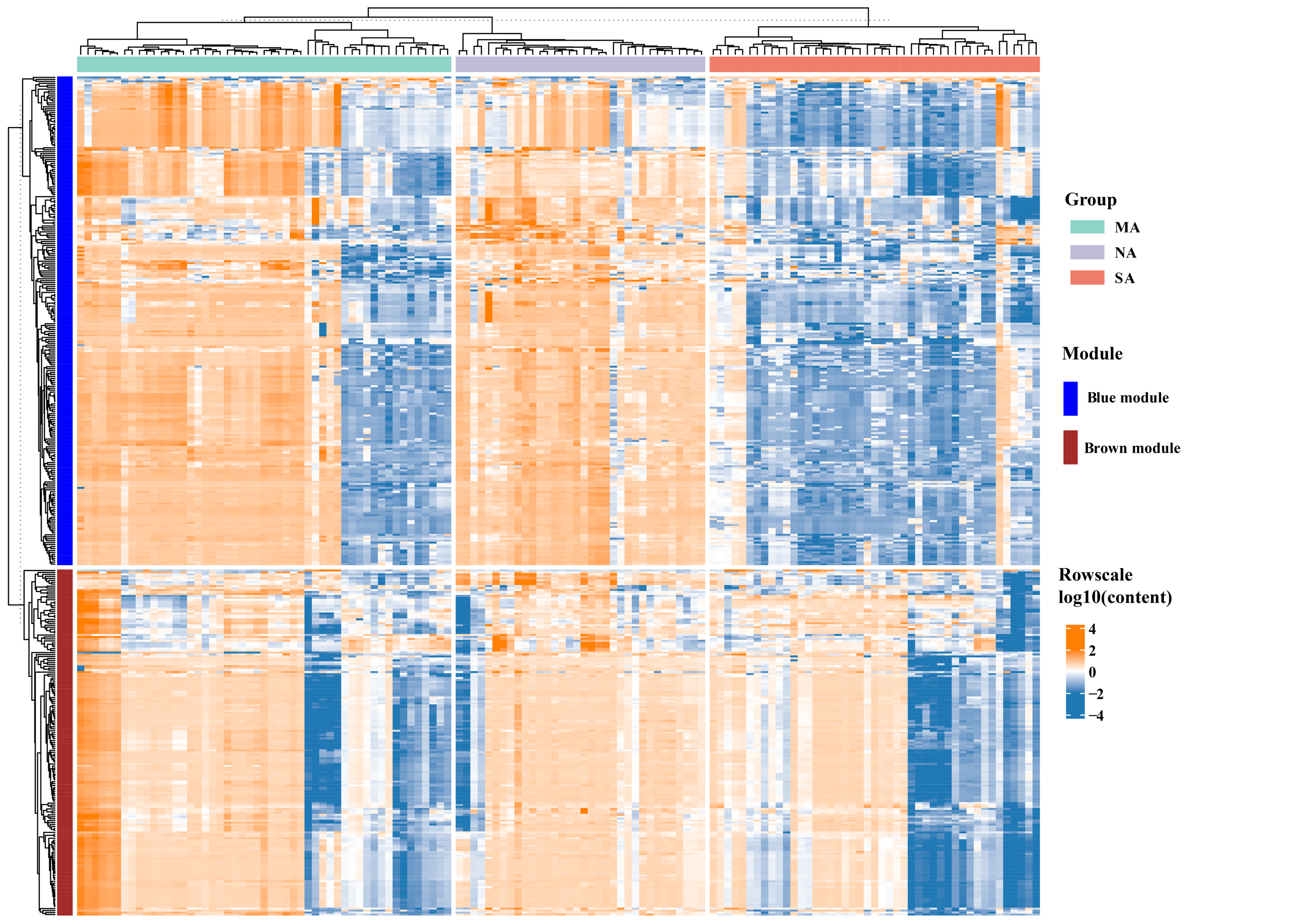

Supplement: Web_Material_uhae167 [file web_material_uhae167.zip › Figure S14.tif]

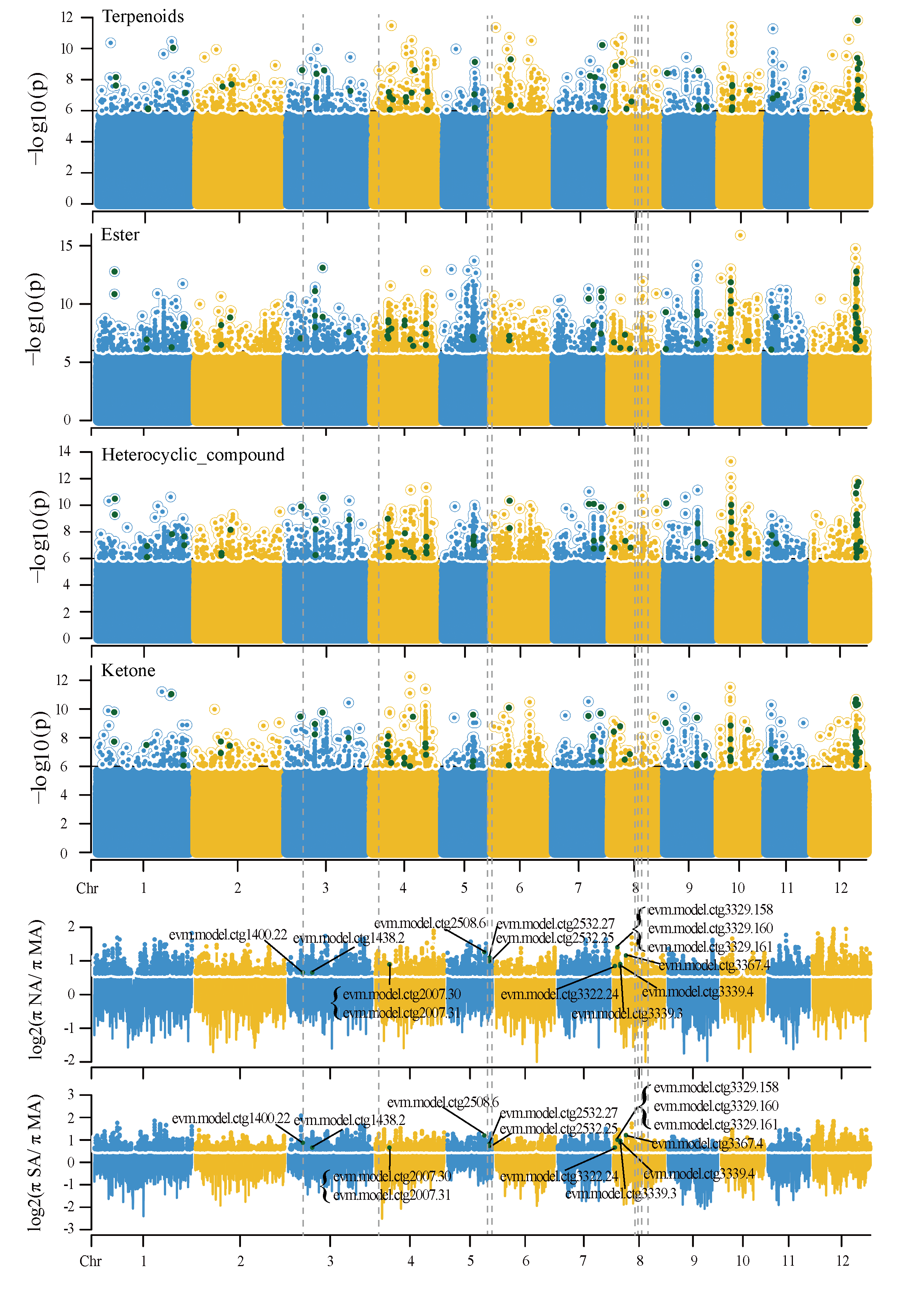

Supplement: Web_Material_uhae167 [file web_material_uhae167.zip › Figure S15.tif]

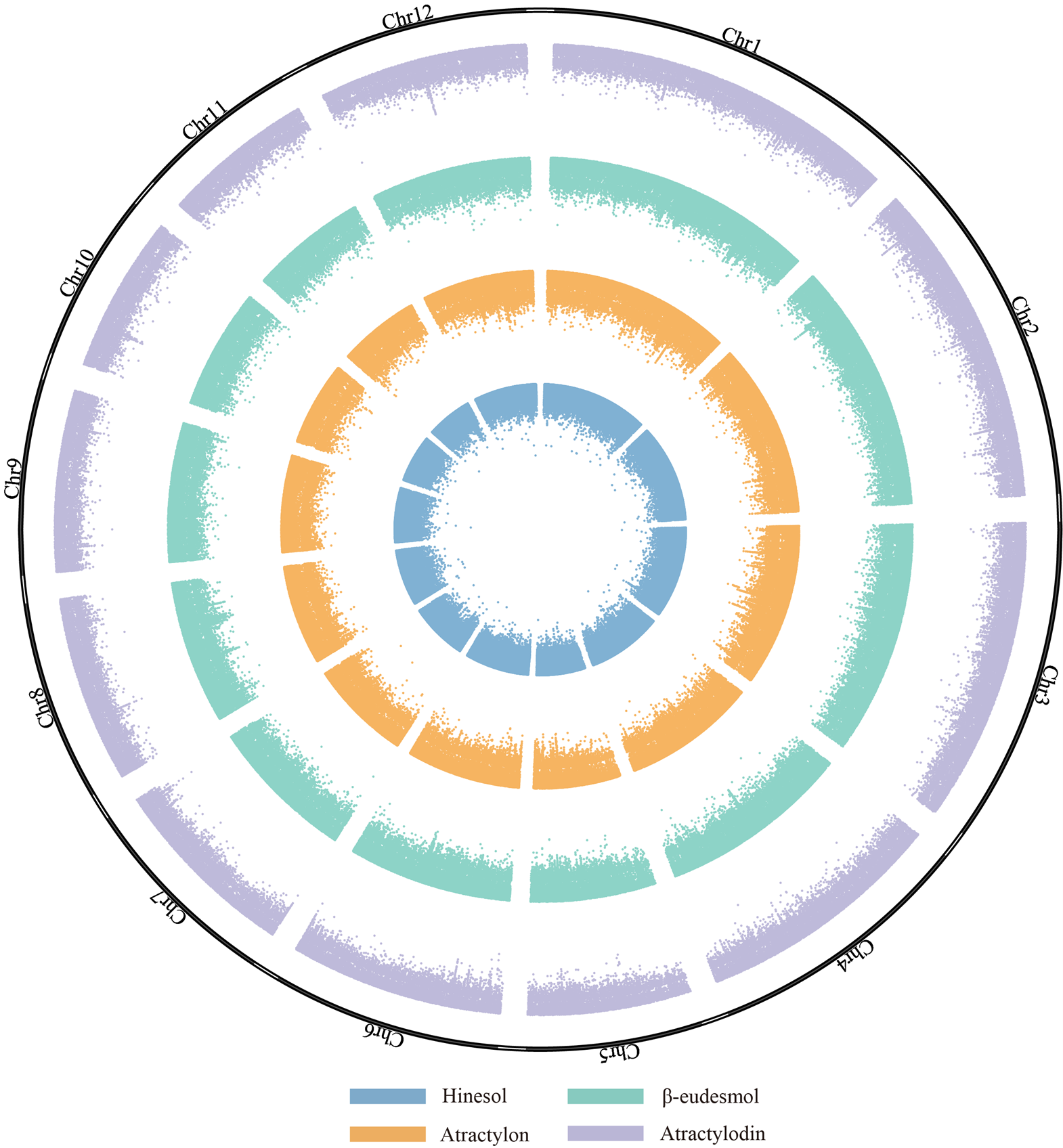

Supplement: Web_Material_uhae167 [file web_material_uhae167.zip › Figure S16.tif]

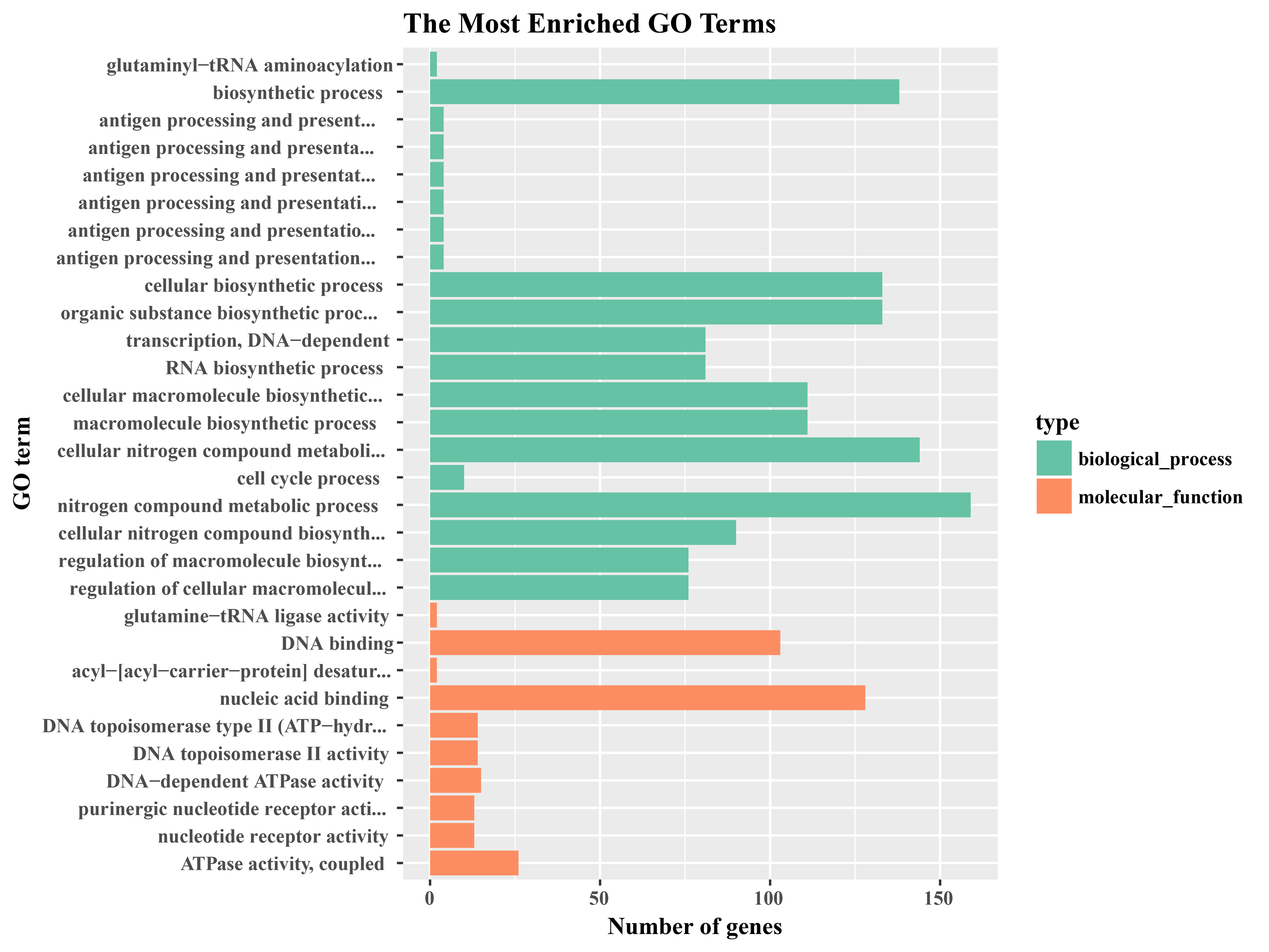

Supplement: Web_Material_uhae167 [file web_material_uhae167.zip › Figure S17.tif]

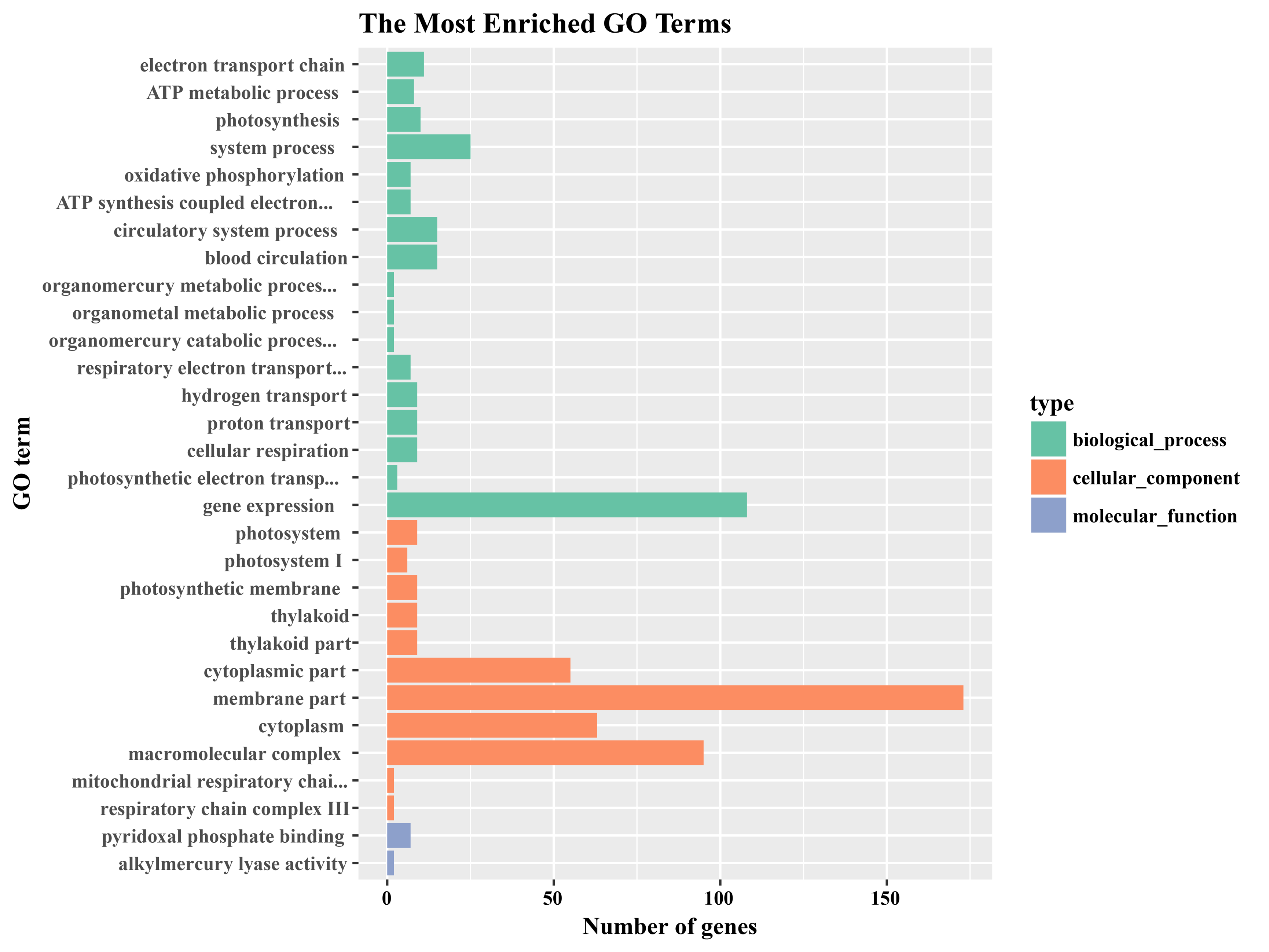

Supplement: Web_Material_uhae167 [file web_material_uhae167.zip › Figure S18.tif]

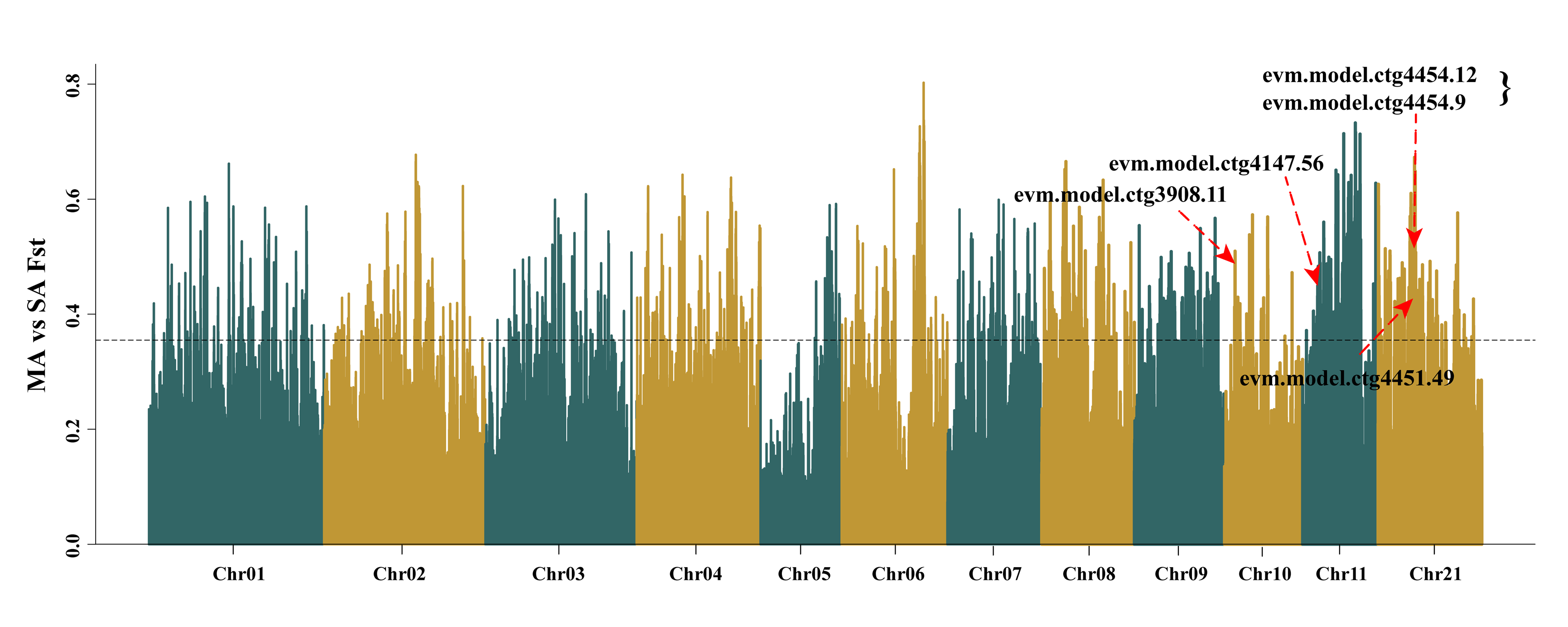

Supplement: Web_Material_uhae167 [file web_material_uhae167.zip › Figure S19.tif]

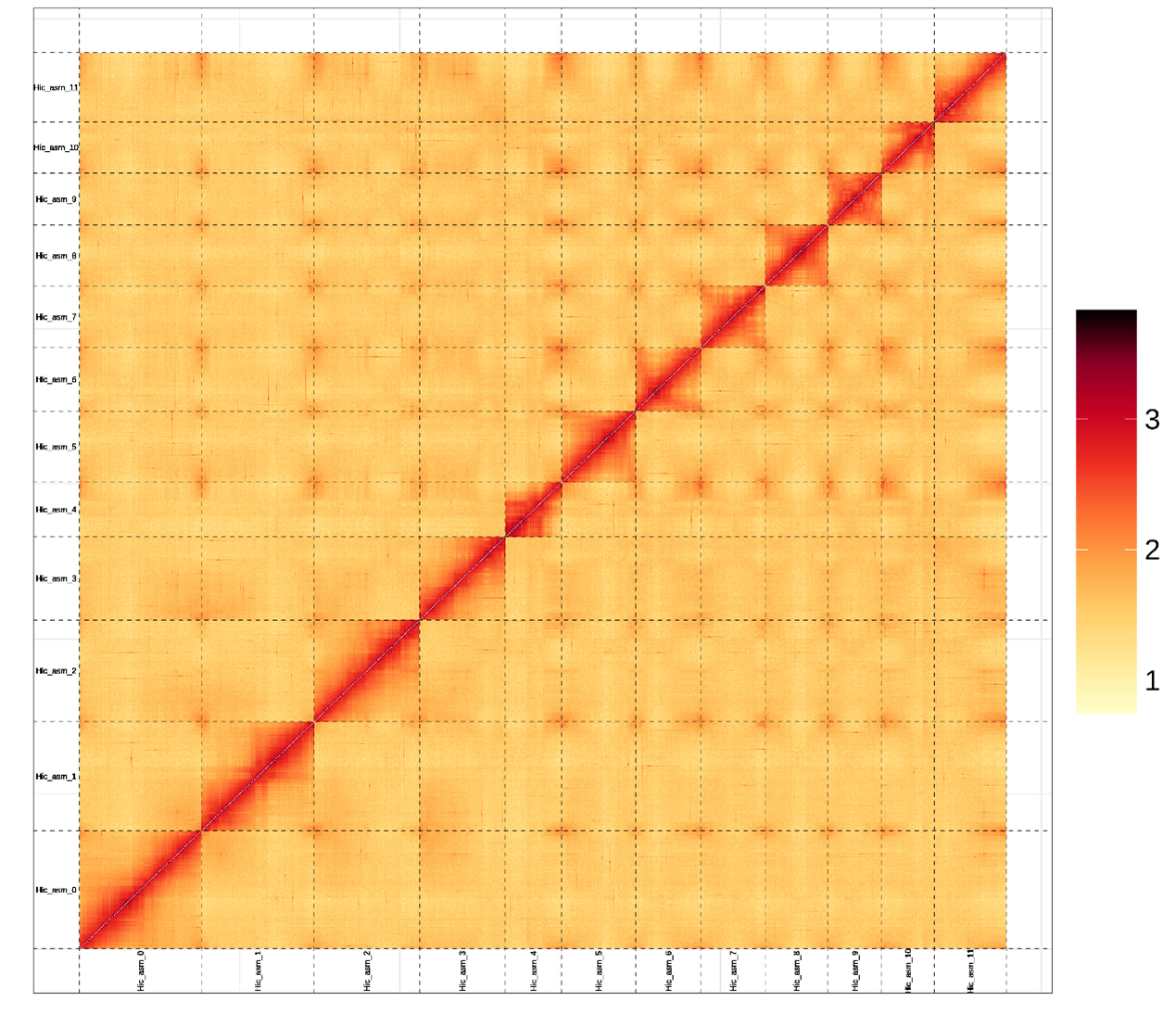

Supplement: Web_Material_uhae167 [file web_material_uhae167.zip › Figure S2.tif]

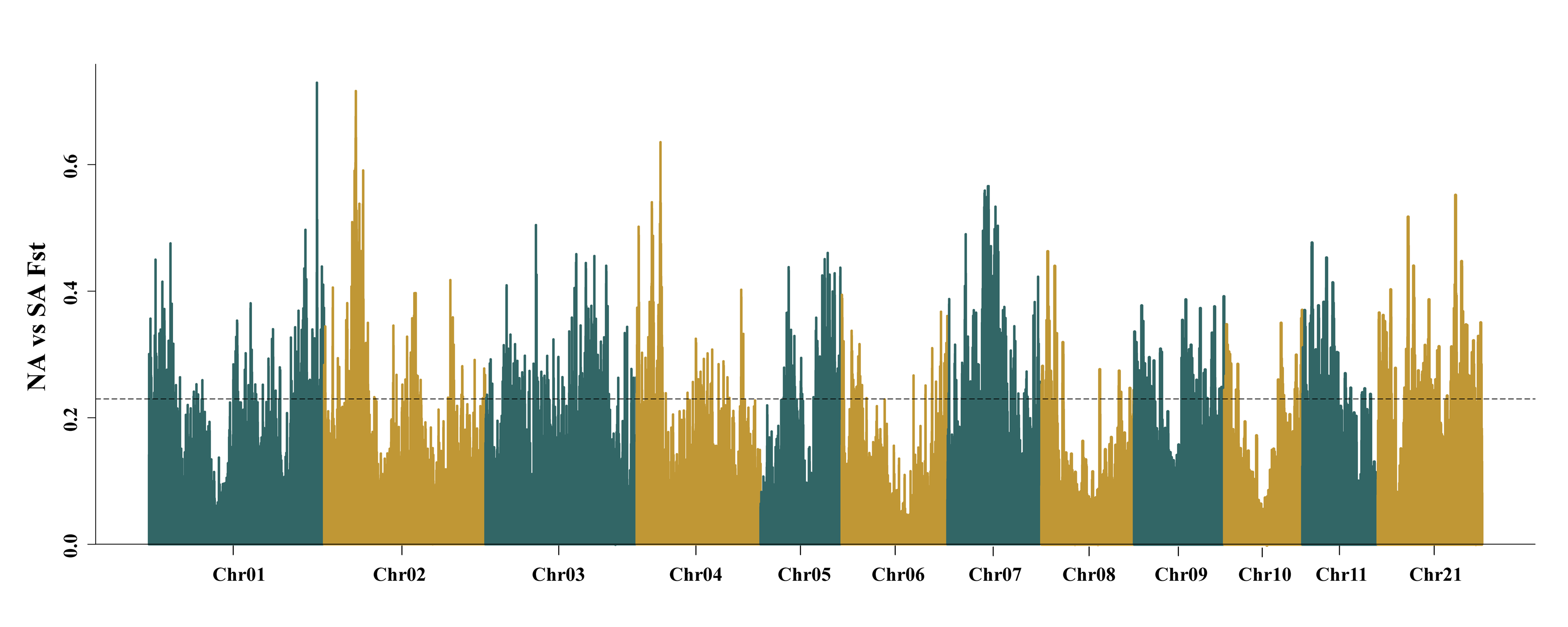

Supplement: Web_Material_uhae167 [file web_material_uhae167.zip › Figure S20.tif]

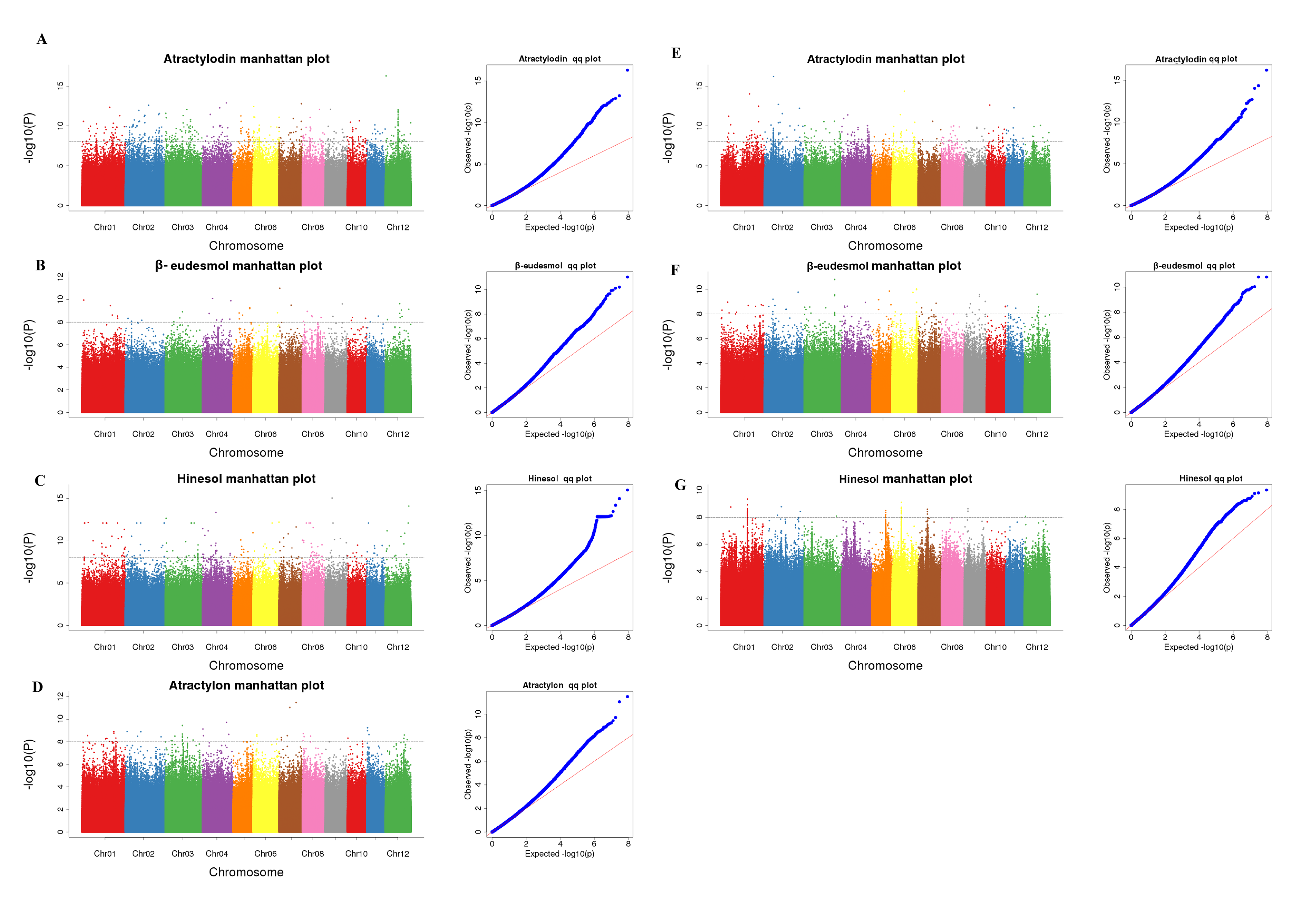

Supplement: Web_Material_uhae167 [file web_material_uhae167.zip › Figure S21.tif]

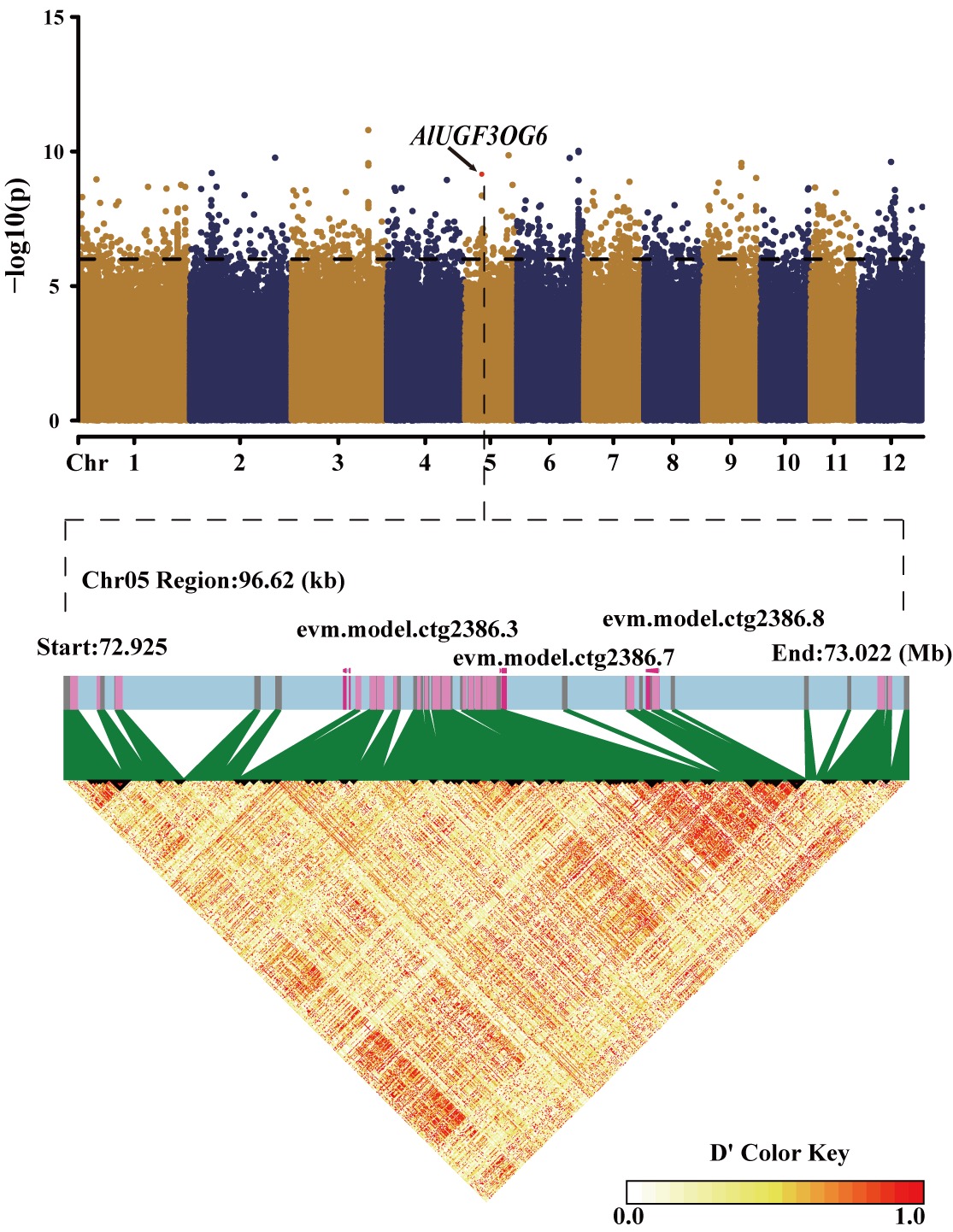

Supplement: Web_Material_uhae167 [file web_material_uhae167.zip › Figure S22.jpg]

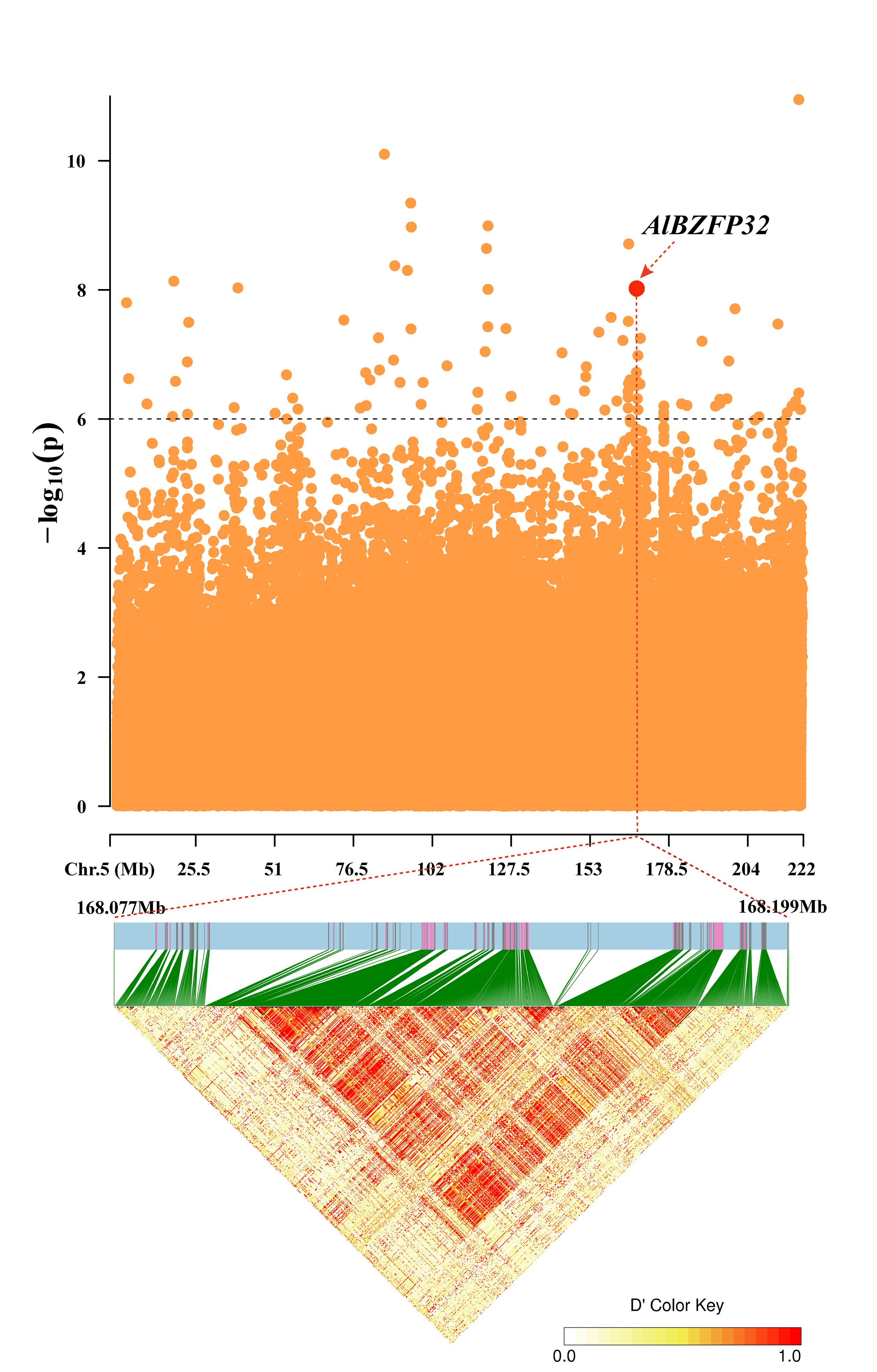

Supplement: Web_Material_uhae167 [file web_material_uhae167.zip › Figure S23.tif]

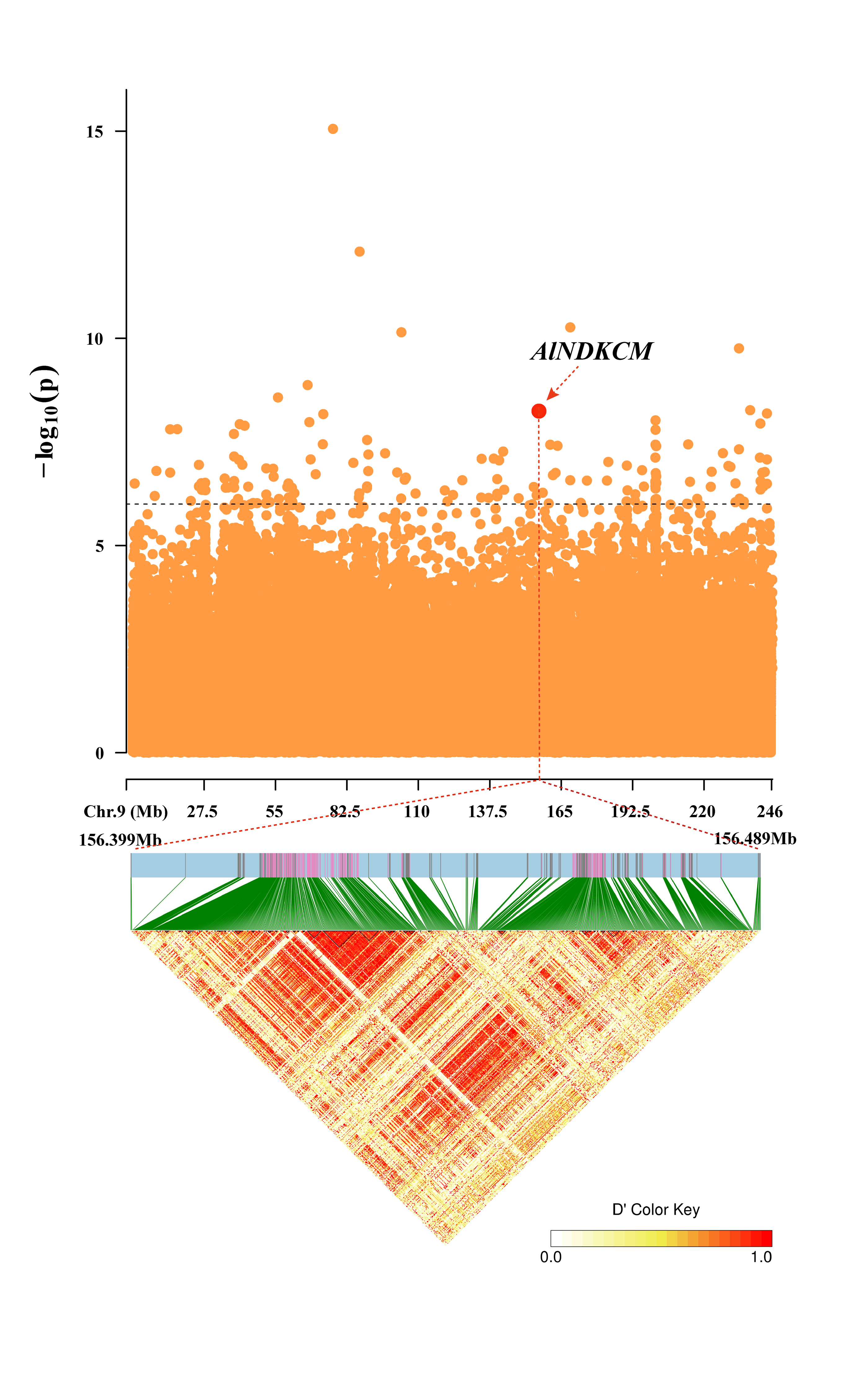

Supplement: Web_Material_uhae167 [file web_material_uhae167.zip › Figure S24.tif]

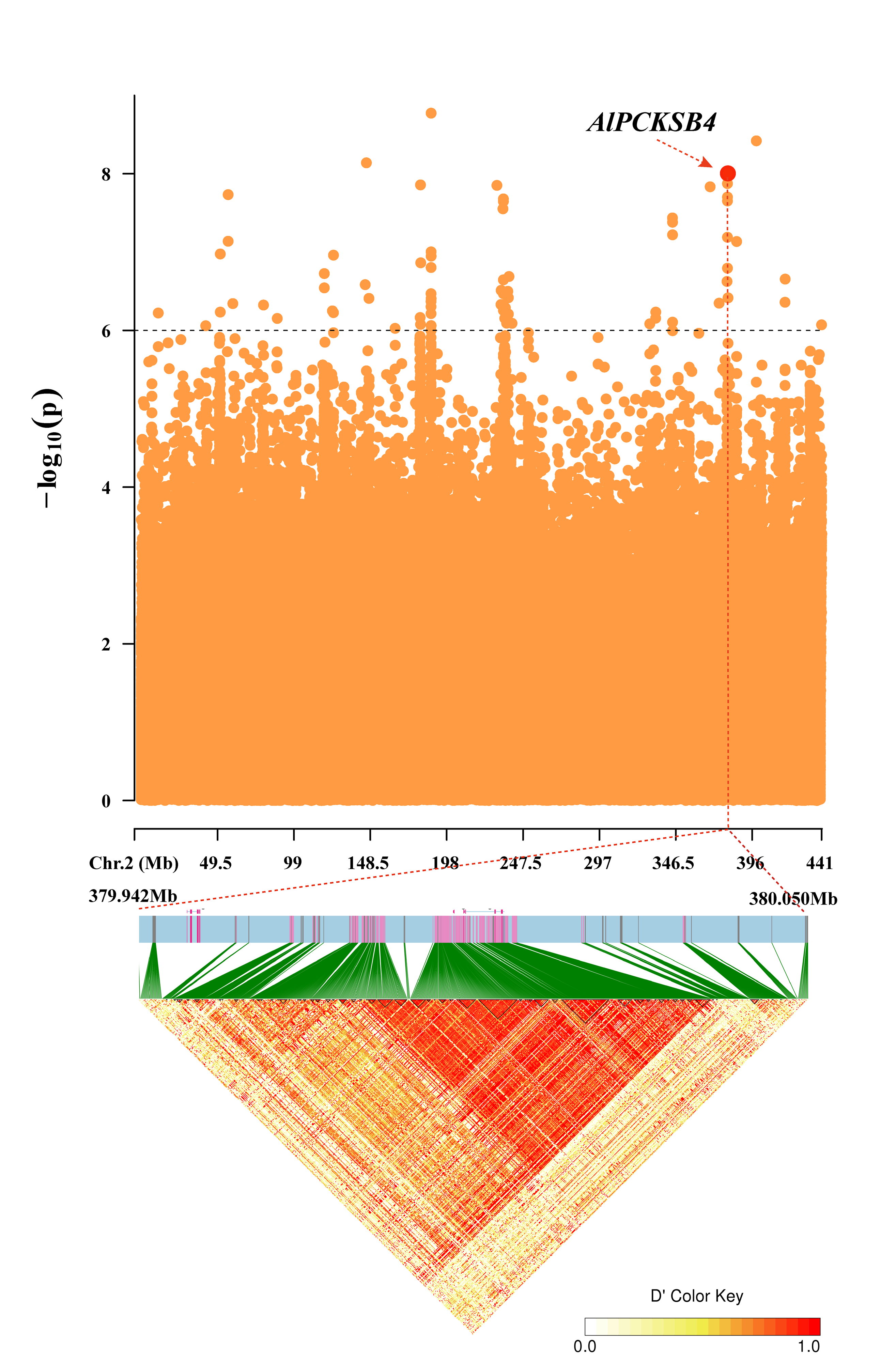

Supplement: Web_Material_uhae167 [file web_material_uhae167.zip › Figure S25.tif]

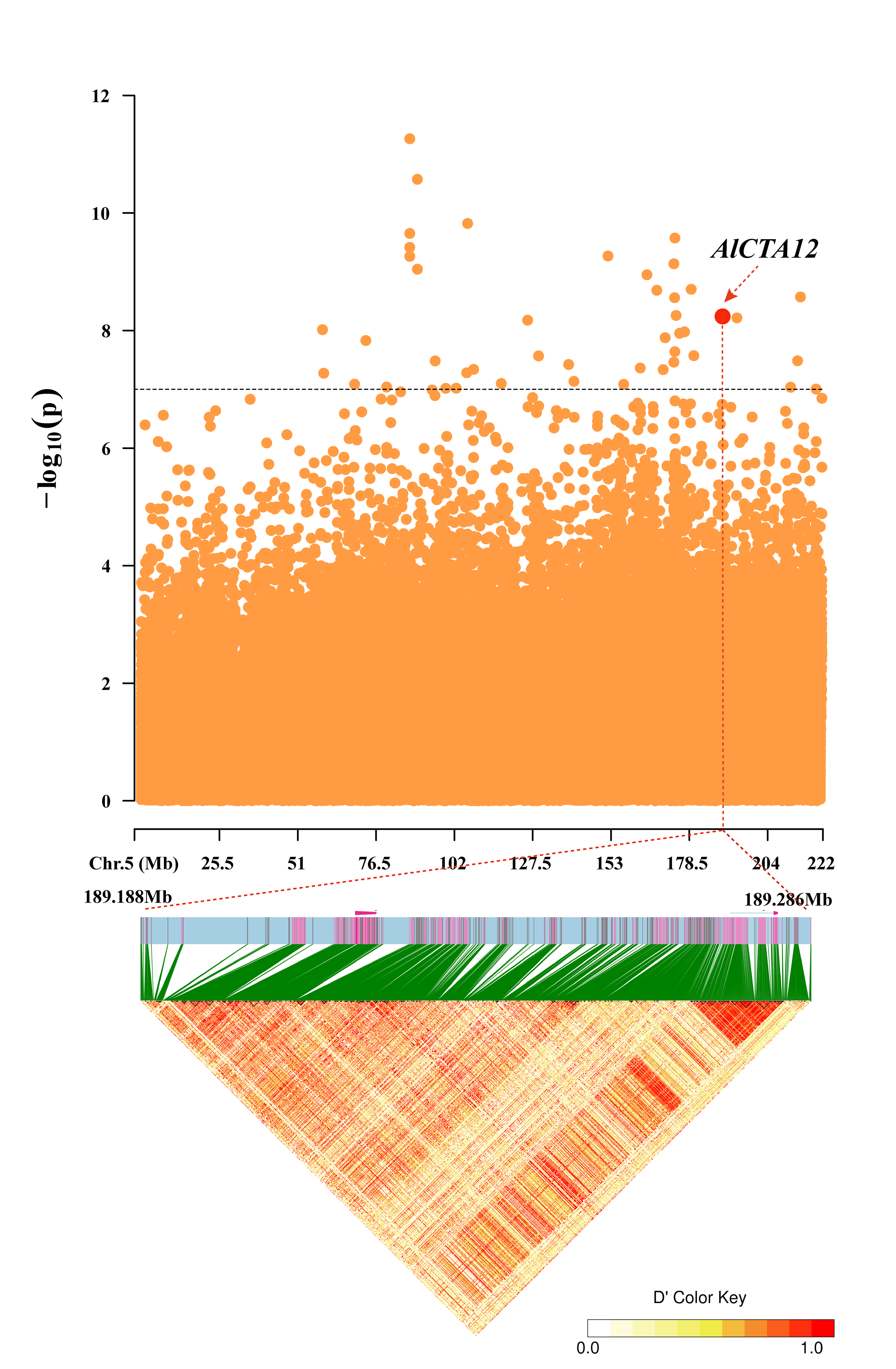

Supplement: Web_Material_uhae167 [file web_material_uhae167.zip › Figure S26.tif]

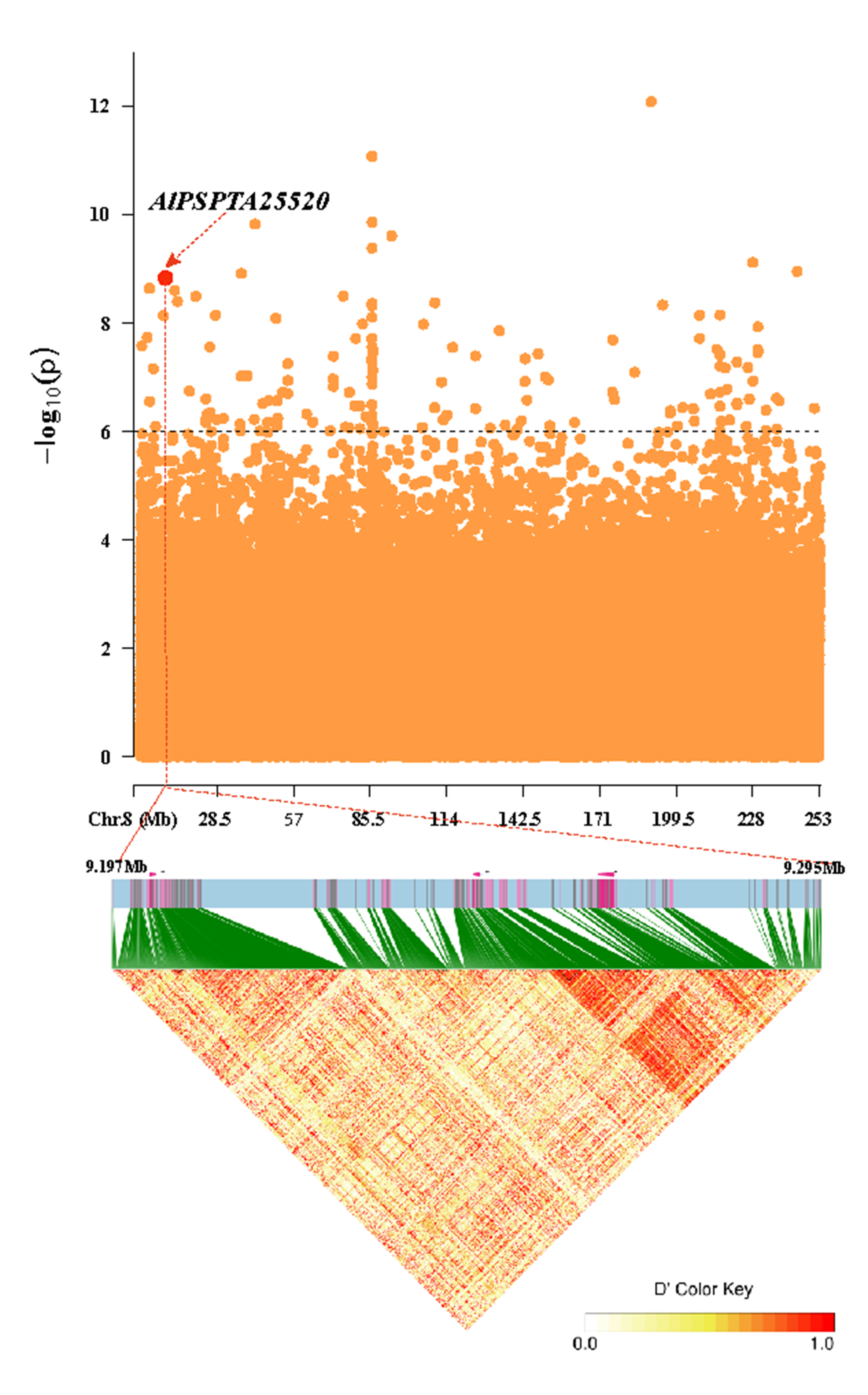

Supplement: Web_Material_uhae167 [file web_material_uhae167.zip › Figure S27.tif]

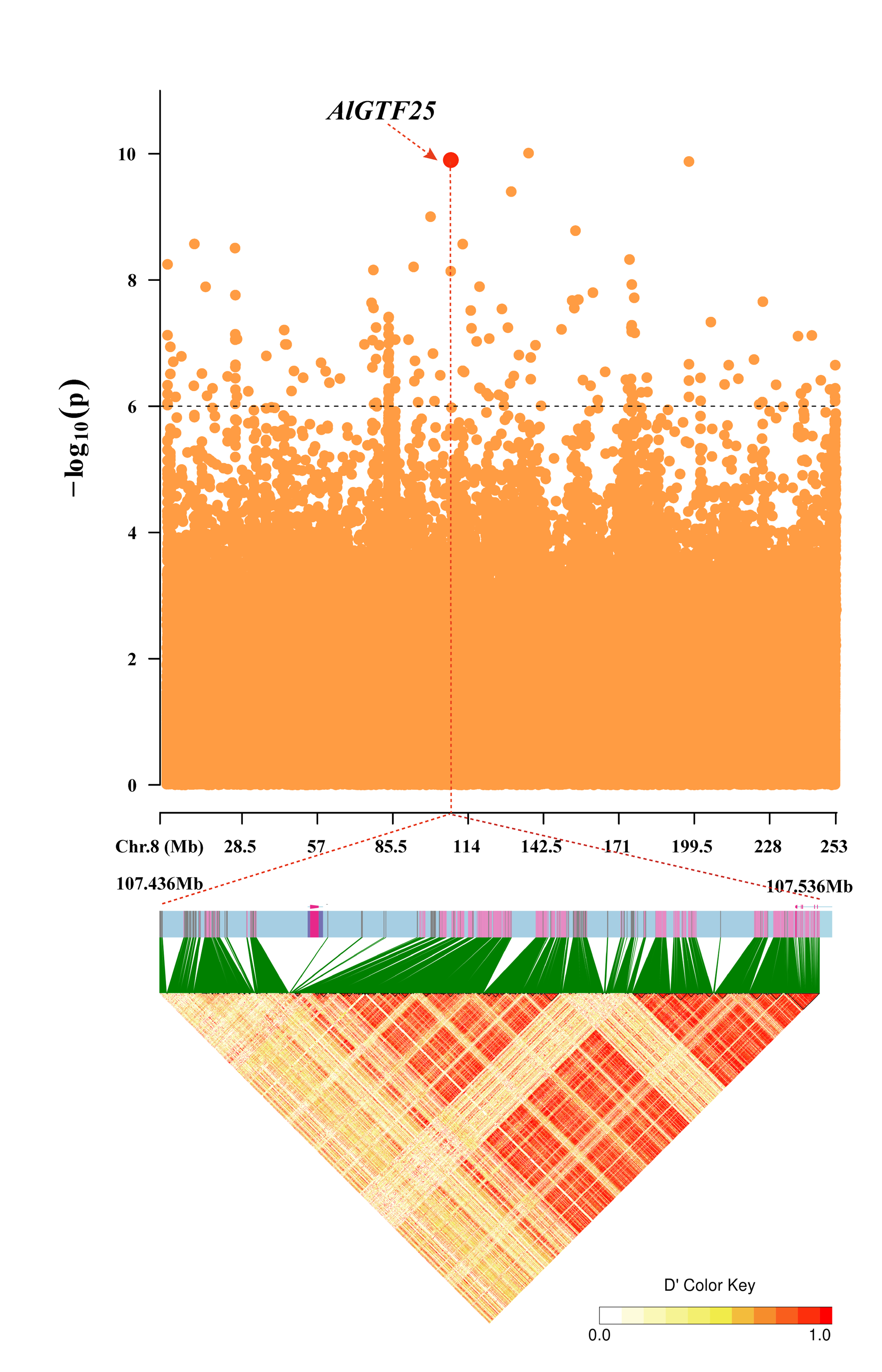

Supplement: Web_Material_uhae167 [file web_material_uhae167.zip › Figure S28.tif]

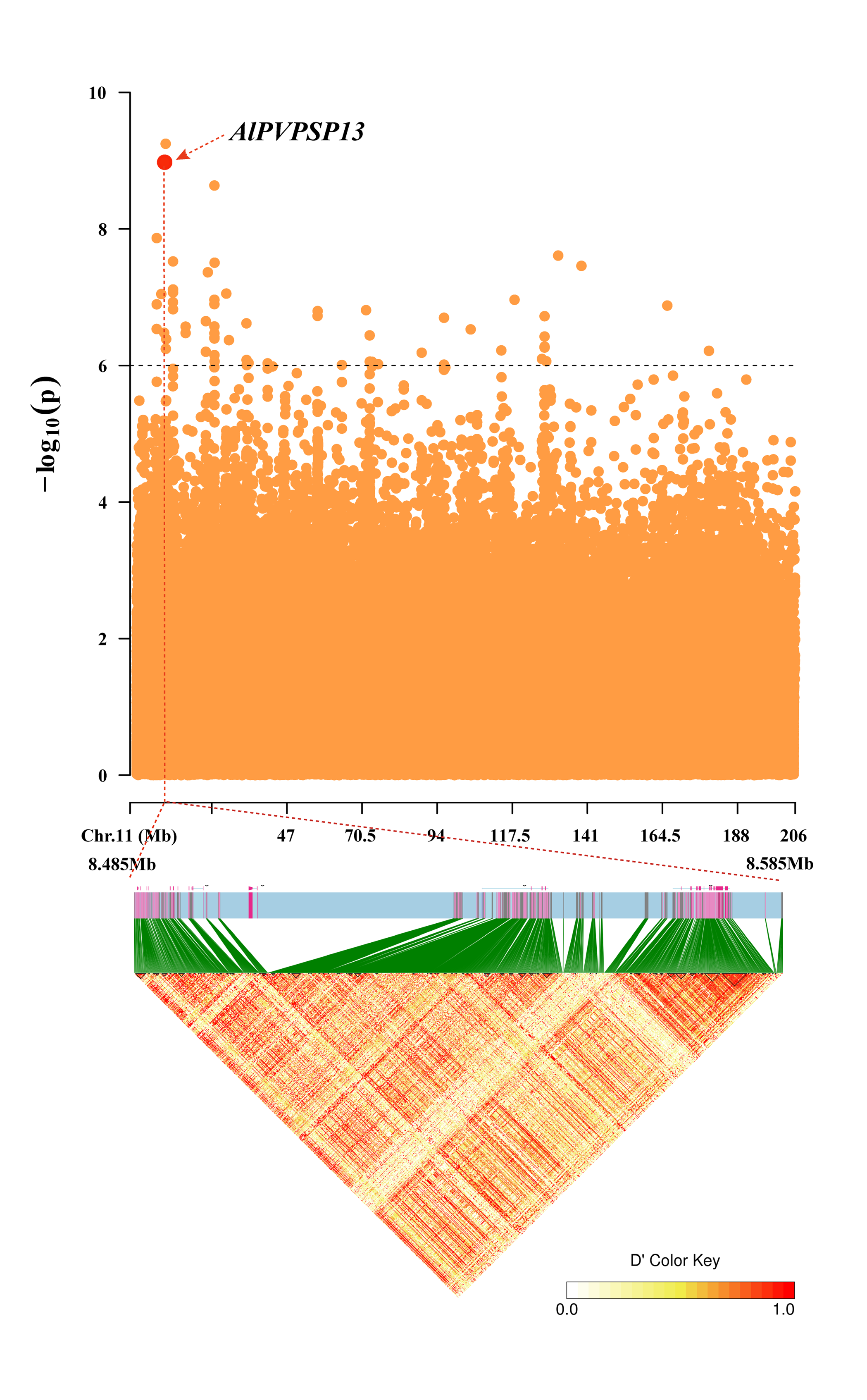

Supplement: Web_Material_uhae167 [file web_material_uhae167.zip › Figure S29.tif]

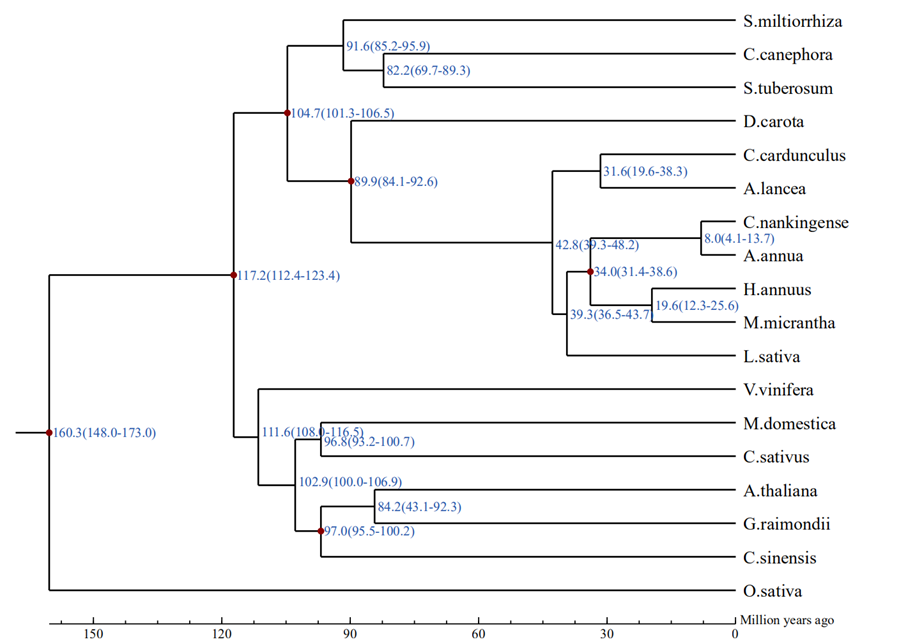

Supplement: Web_Material_uhae167 [file web_material_uhae167.zip › Figure S3.png]

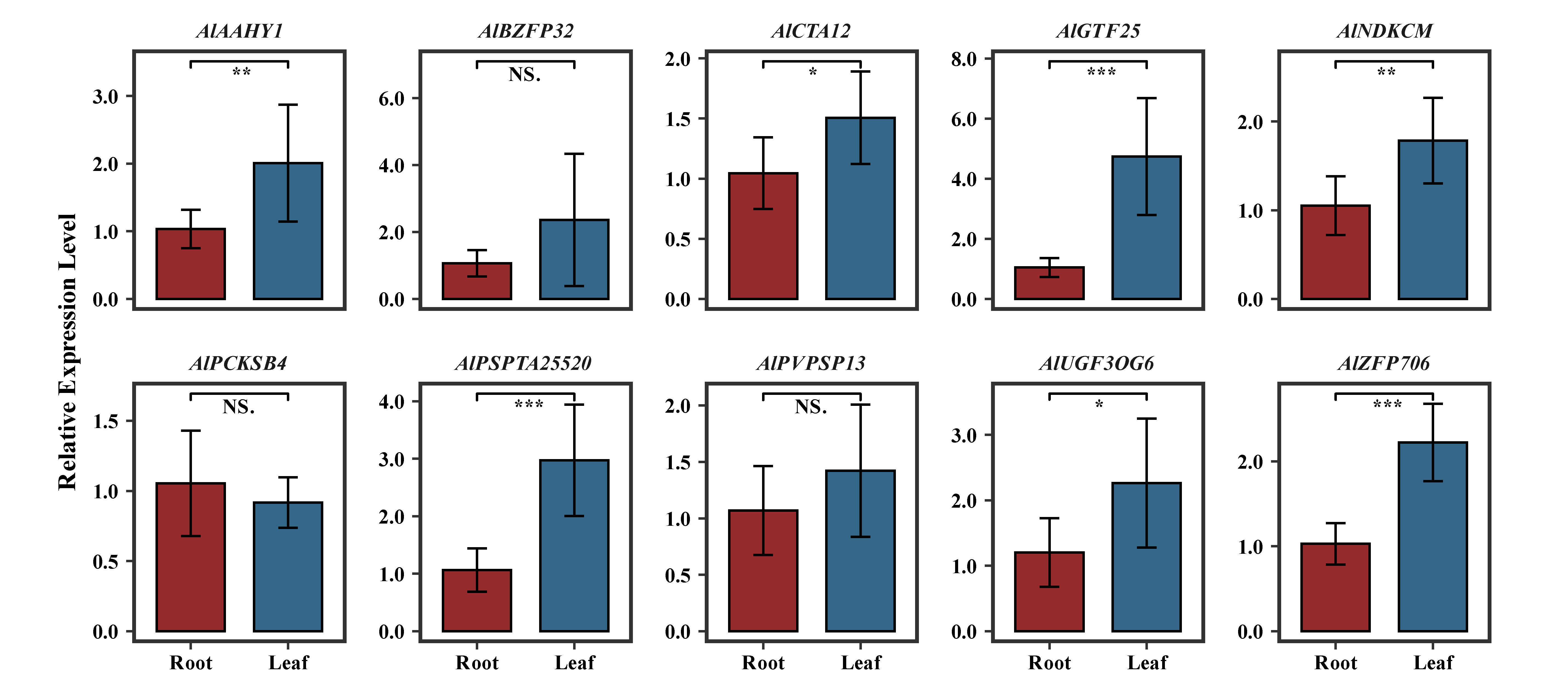

Supplement: Web_Material_uhae167 [file web_material_uhae167.zip › Figure S30.tif]

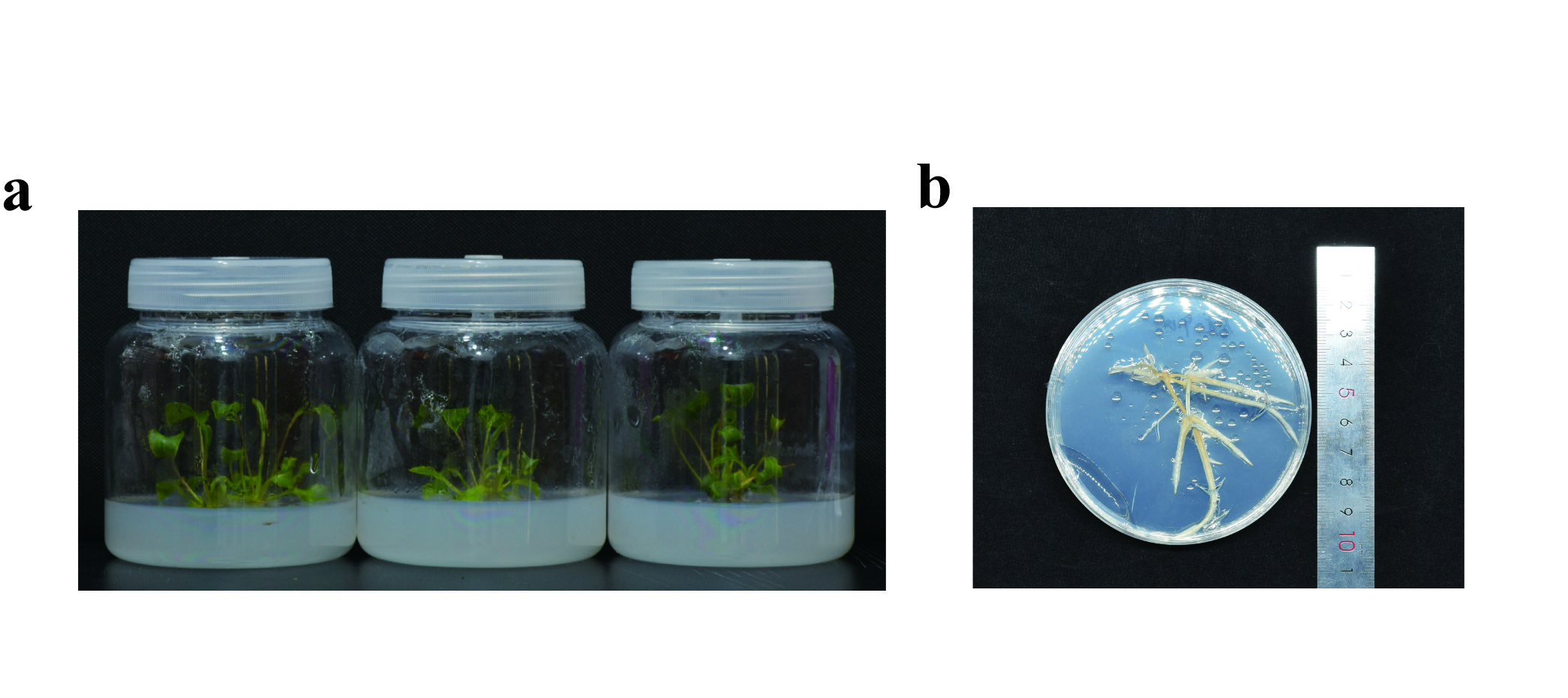

Supplement: Web_Material_uhae167 [file web_material_uhae167.zip › Figure S31.jpg]

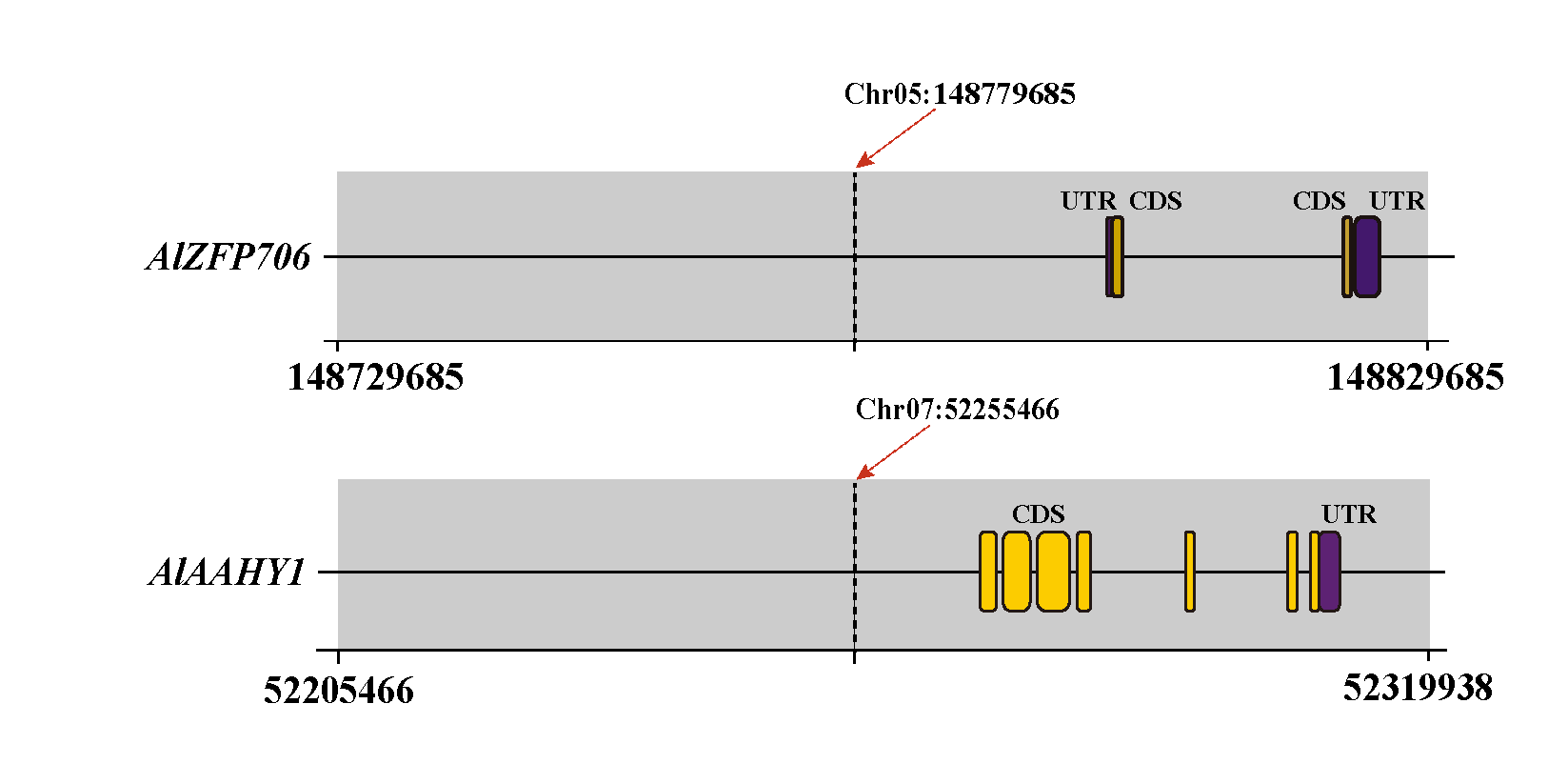

Supplement: Web_Material_uhae167 [file web_material_uhae167.zip › Figure S32.tif]

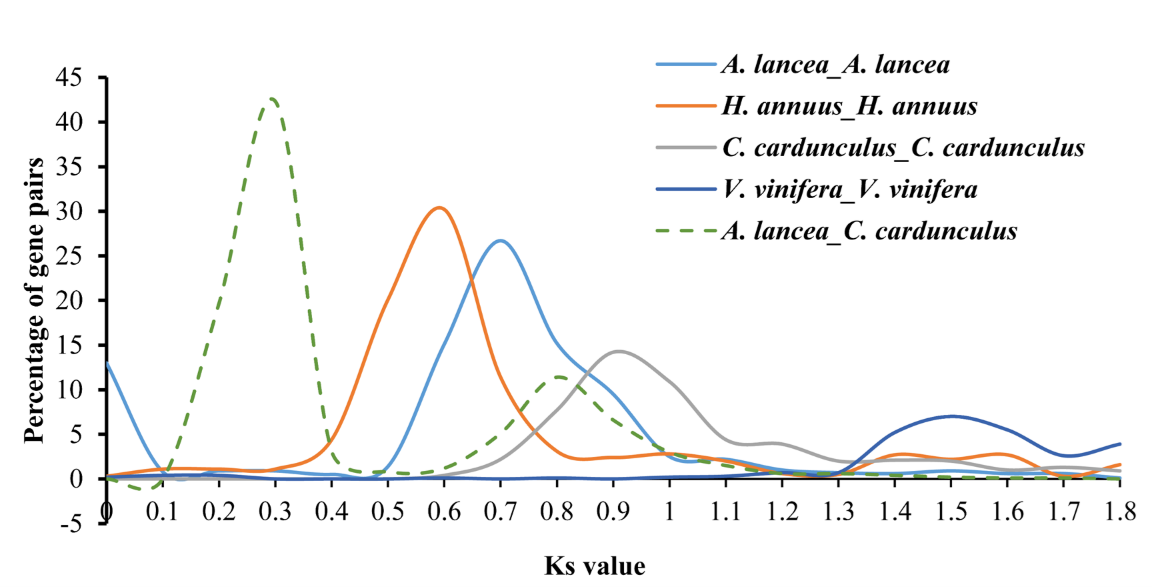

Supplement: Web_Material_uhae167 [file web_material_uhae167.zip › Figure S4.tif]

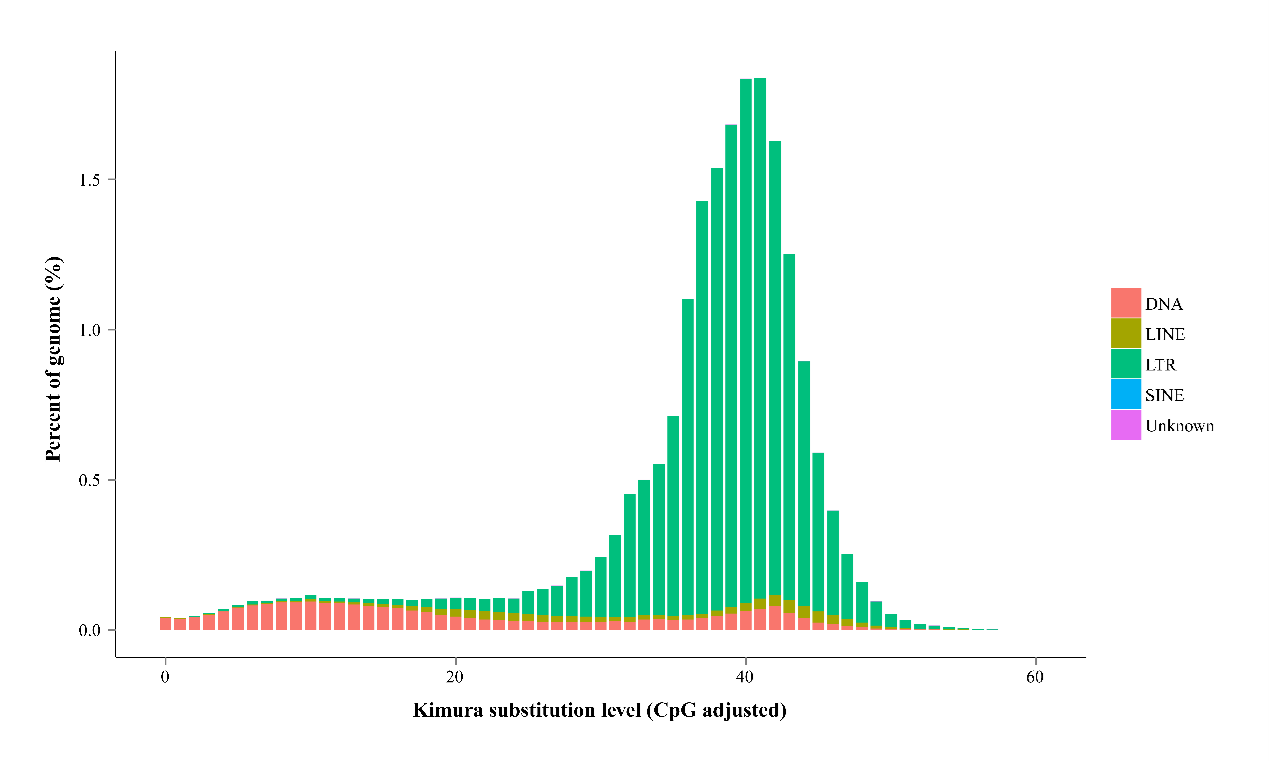

Supplement: Web_Material_uhae167 [file web_material_uhae167.zip › Figure S5.tif]

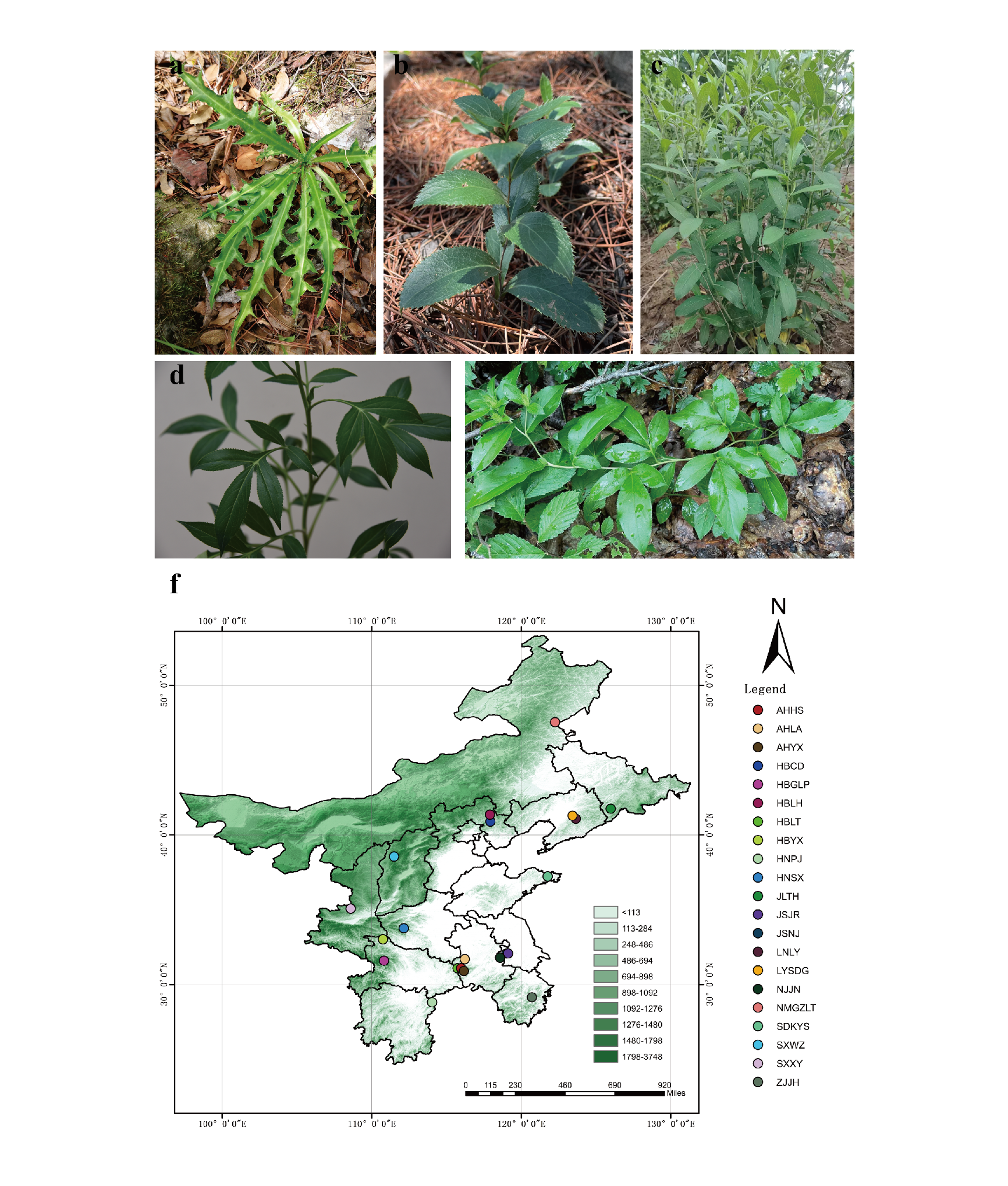

Supplement: Web_Material_uhae167 [file web_material_uhae167.zip › Figure S6.tif]

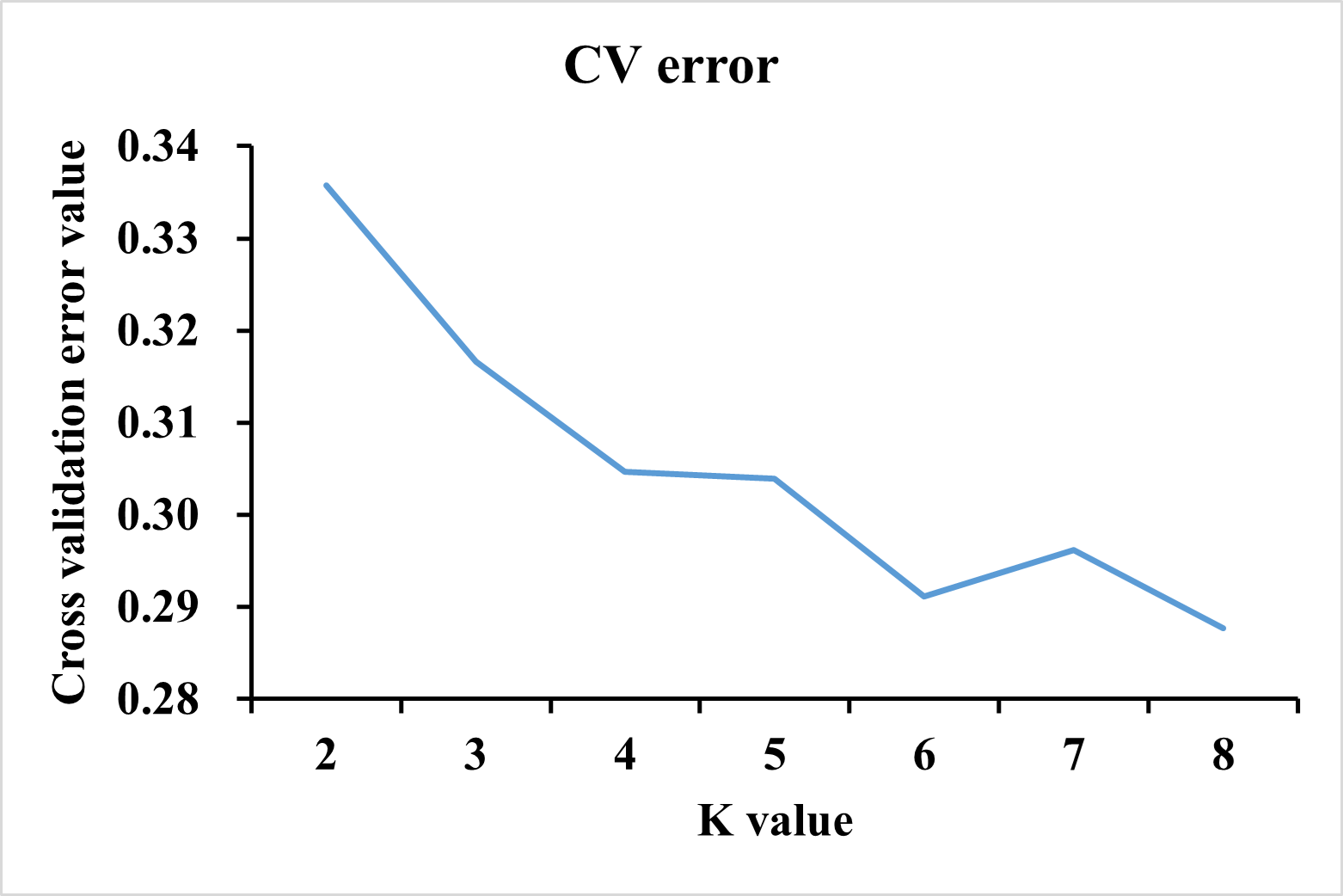

Supplement: Web_Material_uhae167 [file web_material_uhae167.zip › Figure S7.tif]

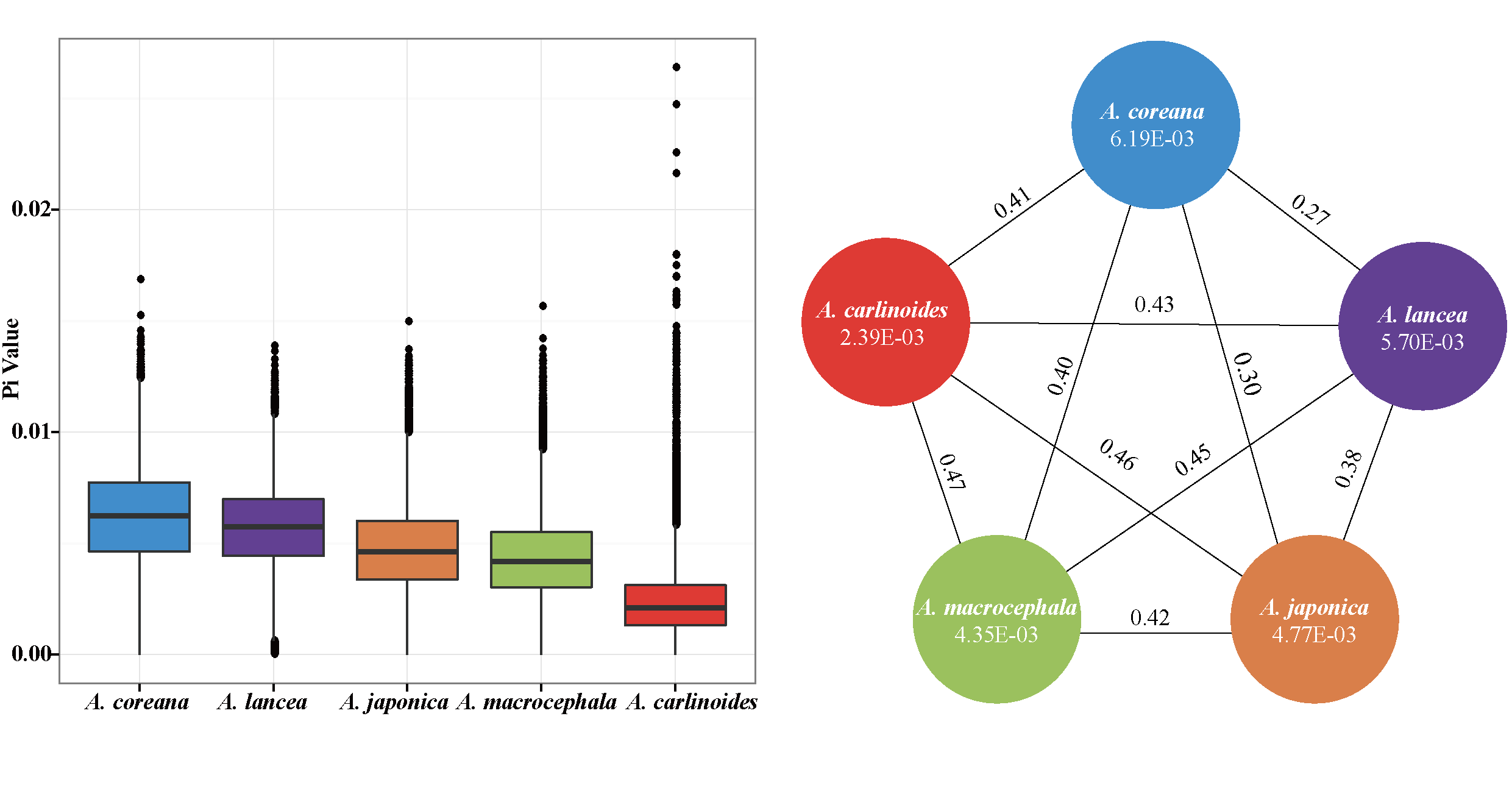

Supplement: Web_Material_uhae167 [file web_material_uhae167.zip › Figure S8.tif]

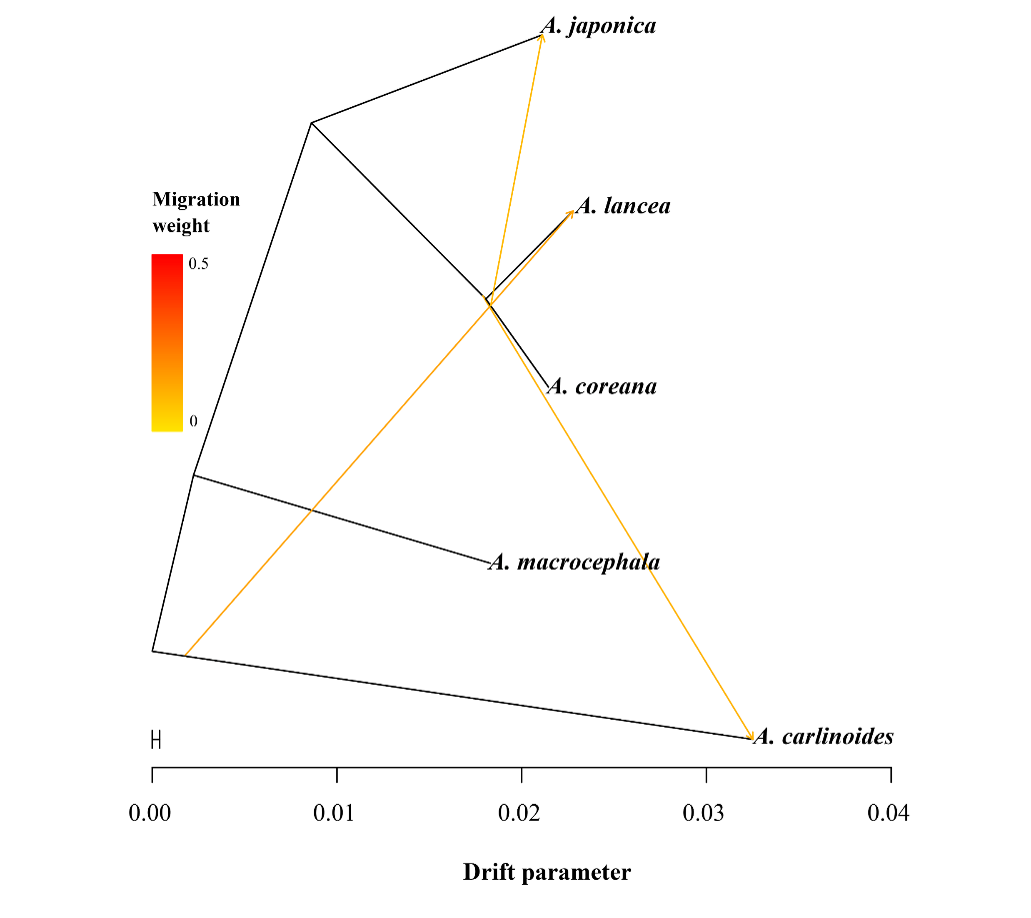

Supplement: Web_Material_uhae167 [file web_material_uhae167.zip › Figure S9.tif]
